# Carfilzomib Improves Bone Metabolism in Patients with Advanced Relapsed/Refractory Multiple Myeloma: Results of the CarMMa Study

**DOI:** 10.3390/cancers13061257

**Published:** 2021-03-12

**Authors:** Evangelos Terpos, Ioannis Ntanasis-Stathopoulos, Eirini Katodritou, Marie-Christine Kyrtsonis, Vassiliki Douka, Emmanouil Spanoudakis, Athanasios Papatheodorou, Evangelos Eleutherakis-Papaiakovou, Nikolaos Kanellias, Maria Gavriatopoulou, Polyzois Makras, Efstathios Kastritis, Meletios A Dimopoulos

**Affiliations:** 1 Department of Clinical Therapeutics, Alexandra General Hospital, School of Medicine, National and Kapodistrian University of Athens, PS 11528 Athens, Greece; johnntanasis@med.uoa.gr (I.N.-S.); mdeleutherakis@gmail.com (E.E.-P.); nick.kanellias@gmail.com (N.K.); mariagabria@gmail.com (M.G.); ekastritis@gmail.com (E.K.); mdimop@med.uoa.gr (M.A.D.); 2 Department of Hematology, Theagenio Cancer Hospital, PS 54639 Thessaloniki, Greece; eirinikatodritou@gmail.com; 3 First Department of Propedeutic Internal Medicine, School of Medicine, National and Kapodistrian University of Athens, PS 11528 Athens, Greece; kyrtsoni@med.uoa.gr; 4 Department of Hematology and Bone Marrow Transplantation Unit, General Hospital “G.Papanikolaou”, PS 57010 Thessaloniki, Greece; vassiliki.douka@gmail.com; 5 Department of Hematology, Faculty of Medicine, Democritus University of Thrace, PS 68131 Alexandroupolis, Greece; emmanouilspanoudakis@yahoo.com; 6 Department of Medical Research, 251 General Air-Force Hospital, PS 11525 Athens, Greece; atpapath@med.uoa.gr (A.P.); makras@internet.gr (P.M.)

**Keywords:** multiple myeloma, carfilzomib, skeletal-related events, bone disease, bone metabolism, osteocalcin, procollagen type I N propeptide (PINP), C-telopeptide of collagen type 1 (CTX), tartrate-resistant acid phosphatase-5b (TRACP-5B), nuclear factor kappa-B ligand (RANKL)

## Abstract

**Simple Summary:**

Carfilzomib with dexamethasone is an important therapeutic option for patients with relapsed/refractory multiple myeloma. We sought to evaluate the effect of this regimen on the bone-related outcomes, which are associated with both quality of life and survival. Among 25 patients, less than one third experienced a new skeletal-related event during treatment, even in the absence of any bone-targeted agent. Interestingly, there was a significant decrease in serum biomarkers of bone resorption, which was at least partially due to the sRANKL/OPG ratio reduction. Furthermore, Kd produced an increase in markers of bone formation. Importantly, these changes were independent of myeloma response to treatment. Therefore, the combination of carfilzomib and dexamethasone improves bone metabolism and bone health in patients with advanced multiple myeloma.

**Abstract:**

Carfilzomib with dexamethasone (Kd) is a well-established regimen for the treatment of relapsed/refractory multiple myeloma (RRMM). There is limited information for the effects of Kd on myeloma-related bone disease. This non-interventional study aimed to assess skeletal-related events (SREs) and bone metabolism in patients with RRMM receiving Kd, in the absence of any bone-targeted agent. Twenty-five patients were enrolled with a median of three prior lines of therapy; 72% of them had evidence of osteolytic bone disease at study entry. During Kd treatment, the rate of new SREs was 28%. Kd produced a clinically relevant (≥30%) decrease in C-telopeptide of collagen type-1 (*p* = 0.048) and of tartrate-resistant acid phosphatase-5b (*p* = 0.002) at 2 months. This reduction was at least partially due to the reduction in the osteoclast regulator RANKL/osteoprotegerin ratio, at 2 months (*p* = 0.026). Regarding bone formation, there was a clinically relevant increase in osteocalcin at 6 months (*p* = 0.03) and in procollagen type I N-propeptide at 8 months post-Kd initiation. Importantly, these bone metabolism changes were independent of myeloma response to treatment. In conclusion, Kd resulted in a low rate of SREs among RRMM patients, along with an early, sustained and clinically relevant decrease in bone resorption, which was accompanied by an increase in bone formation, independently of myeloma response and in the absence of any bone-targeted agent use.

## 1. Introduction

Bone disease is a cardinal feature of multiple myeloma (MM) and it is attributed to the deregulation of the fine tuning between bone formation and bone resorption [1]. Patients with myeloma bone disease are at high risk of skeletal-related events (SREs) such as pathological fractures, compression of the spinal canal and need for surgery or radiotherapy due to bone-related complications. SREs add significantly to the disease burden by increasing morbidity, mortality and treatment costs [2,3].

Although treatment of myeloma bone disease is primarily based on bone-targeting agents such as bisphosphonates and denosumab [4], anti-myeloma regimens including proteasome inhibitors seem to exert a beneficial effect on bone metabolism as well [5,6,7,8,9,10,11,12]. In particular, bortezomib has inhibitory effects on osteoclastogenesis, but it also enhances bone formation [13,14,15,16]. Carfilzomib with dexamethasone (Kd) is a well-established regimen for patients with relapsed/refractory multiple myeloma (RRMM) in the clinical practice [17,18,19]. However, clinical data on the net effects of carfilzomib on indices of bone metabolism are limited [5,20].

In this context, we performed a prospective study in order to determine the role of Kd in bone-specific outcomes by evaluating SREs and serum markers of bone metabolism in patients with RRMM.

## 2. Materials and Methods

This was an open-label, prospective, non-interventional, multicenter study aiming to evaluate the effect of Kd combinations in SREs and bone indices in patients with RRMM, in the absence of any bone-targeted agent, who were treated in five myeloma centers in Greece. The study was approved by the institutional review board in accordance with the Declaration of Helsinki and the International Conference on Harmonization for Good Clinical Practice. All patients provided written informed consent before entering into the study. The study was sponsored by the Hellenic Society of Hematology (identifying number 20167750).

### 2.1. Study Objectives

The primary objective of this study was to evaluate the incidence of SREs, including pathological fractures, need for radiotherapy or surgery to the bones, and spinal cord compression during Kd therapy.

Secondary outcomes included the evaluation of serum markers of bone resorption and formation at 4, 8 and 12 months from the initiation of Kd, the 12-month overall survival (OS) and progression-free survival (PFS) rates, the time to next treatment (TtNT) and the safety profile of Kd during the study period.

### 2.2. Eligibility Criteria and Treatment Schedule

Carfilzomib in combination with dexamethasone is indicated for the treatment of adult patients with multiple myeloma who have received at least one prior therapy. Adult patients with RRMM who received Kd in the real-world practice according to the approved indication were included in this study.

Carfilzomib was administered twice weekly at days 1, 2, 8, 9, 15 and 16 at 20 mg/m^2^ on days 1 and 2 of cycle 1 and at 56 mg/m^2^ thereafter, along with weekly dexamethasone at 40 mg orally or intravenously, in each 28-day cycle. Kd was administered continuously until disease progression, unacceptable toxicity, death or study withdrawal. Supportive medication for infection and thrombosis prophylaxis were administered according to standard clinical practice. In order to better evaluate the effects of Kd on bone metabolism, no bone-targeted agent (bisphosphonate or denosumab) was given throughout the study period. Dose modifications were applied, as appropriate. Response assessment was based on the International Myeloma Working Group criteria [21].

Patients were treated as per routine medical practice in terms of frequency of visits and clinical and laboratory assessments. Baseline data of the included patients were collected at an enrolment visit up to 7 days prior to initiating treatment with Kd. Subsequently, an observational period started, during which data were collected in 4-weekly intervals (day 1 of each treatment cycle and then every 4 weeks after the end of treatment) for up to 30 months. Patients who discontinued the study treatment were followed up for vital status every 4 weeks, unless informed consent was withdrawn. The end of the study was the last data collection point within the study for the last participating patient, who had a maximum observation period of 12 months.

### 2.3. Evaluation of SREs and Bone Metabolism

Patients were assessed for SREs throughout the study period. SREs included pathological fractures, compression of the spinal canal and need for surgery or radiotherapy due to bone-related complications. Imaging studies (CT or MRI of the respective area of interest) were performed according to the discretion of each treating physician, as per clinical practice.

Bone metabolism was evaluated by the serial measurement of circulating markers of bone resorption (C-terminal cross-linking telopeptide of collagen type I (CTX) and tartrate-resistant acid phosphatase 5b (TRACP-5b)), markers of bone formation (bone-specific alkaline phosphatase (bALP), osteocalcin (OC) and procollagen type I N-terminal propeptide (P1NP)), osteoclast regulators (RANKL, osteoprotegerin (OPG), CC-motif ligand-3 (CCL-3) and activin-A) and osteoblast inhibitors (dickkopf-1 (Dkk-1) and sclerostin) at baseline and every two months post-treatment initiation for a maximum of 12 months (months 2, 4, 6, 8, 10, 12) or until disease progression, whichever occurred first. Baseline biomarker values of patients with RRMM were compared with age- and sex-matched controls (1 patient: 2 controls).

After venipuncture, serum was separated within 4 h and stored at −0 °C until the day of measurement. An enzyme-linked immunosorbent assay (ELISA), according to the manufacturer’s instructions, was used for the detection of serum: CTX (Serum Crosslaps, Immunodiagnostic Systems) with intra- and inter-assay coefficients of variability (CVs) of <3% and <10.9%, respectively; TRACP-5b (BoneTRAP, Immunodiagnostic Systems, Boldon, Tyne & Wear, UK) with intra- and inter-assay CVs of <13.9% and <9.2%, respectively; bALP (Ostase BAP, Immunodiagnostic Systems) with intra- and inter-assay CVs of <6.5% and <6.4%, respectively; OC (N-MID Osteocalcin, Immunodiagnostic Systems Nordic A/S, Herlev, Denmark), with intra- and inter-assay CVs of <2.2% and <5.1%, respectively; P1NP (Abbexa Ltd., Cambridge, UK) with intra- and inter-assay CVs of <10%; sRANKL (Biomedica Medizinprodukte, Gesellschaft GmbH & Co KG, Wien, Austria) with intra- and inter-assay coefficients of variability (CVs) of <5% and <9%, respectively; OPG (Biomedica Medizinprodukte) with intra- and inter-assay CVs of <10% and <8%, respectively; CCL-3 (Quantikine, R&D systems, Minneapolis, MN, USA) with intra- and inter-assay CVs of <3% and <7%, respectively; activin-A (Quantikine, R&D Systems, Minneapolis, MN, USA) with intra- and inter-assay CVs of <4.5% and <8%, respectively; Dkk-1 (Biomedica Medizinprodukte) with intra- and inter-assay CVs of <8% and <12%, respectively. Serum sclerostin was measured using a sandwich-type ELISA by Biomedica Laboratory (Wien, Austria); the detection limit was 0.2 ng/mL (8.9 pmol/L); the standard range was set from 0.33 to 5.4 ng/mL (15–240 pmol/L); and the CV for intra-assay was 4–6%, while for inter-assay, it was 5–7%. All samples from the same patient were measured on the same ELISA plate, according to manufacturers’ respective instructions.

Bone markers were also evaluated in age- and gender-matched controls at a ratio of 1:2 for patients and controls, respectively. Each control was examined to ensure that there was no evidence of bone disease such as osteoporosis or osteoarthritis (patients with BMD of > −2.0 were excluded), no receipt of medication that could alter the normal bone turnover during the last 6 months (this cut-off is a potential limitation as bisphosphonates have a longer skeletal half-life) and no evidence of infection or autoimmune disease on the day of sampling. For the assessment of osteoporosis, all controls had bone mineral density measurements using DXA in both lumbar spine and femoral neck.

### 2.4. Statistical Analysis

All patients who received at least one dose of the study treatment were considered for data analysis. All statistical analyses and generation of tables and patient data listings were performed using SAS^®^ statistical analysis software (v. 9.4). Summary statistics based on frequency tables were used for categorical variables. For continuous variables, descriptive statistics (mean, median, standard deviation, Q1, Q3, minimum and maximum values) were applied. The incidence of SREs was summarized in terms of number (and percentage) of patients with events and number of events per patient. To evaluate the statistical significance of the changes in biomarkers over time, linear repeated measures models were fitted, and the respective *p*-values are presented. The level of statistical significance was set at <0.05. In order to evaluate absolute changes, the log-transformed absolute biomarker values at each time point were used as dependent variables. The variable “visit” (i.e., cycle) was included in the models as a fixed effect. Furthermore, a cut-off of ≥30% change from baseline was considered as clinically relevant regarding the values of biomarkers of bone metabolism. As there are no data for myeloma patients, we used the ≥30% change of a marker as clinically significant, based on osteoporosis studies [22,23]. For the comparison of baseline biomarker levels in patients versus controls, the Mann–Whitney U test was used. Similarly, the Mann–Whitney U test and Kruskal–Wallis test were used, as applicable, for comparing the distribution of quantitative variables among subgroups of interest. Fisher’s exact test was used for examining the associations between two categorical variables. PFS was defined as the time, in months, from the treatment start to the date of the first documented tumor progression or death due to any cause, whichever came first. TtNT was defined as the time, in months, from treatment start to the date of next anti-neoplastic therapy or death from any cause, whichever came first. OS was defined as the time, in months, from treatment start to the date of death from any cause. Survival functions were estimated using the Kaplan–Meier method, and the values of the median, 12-month and two-sided confidence intervals (CI) for PFS, TtNT and OS were computed. All *p*-values were two-sided and confidence intervals refer to 95% boundaries, unless otherwise indicated.

## 3. Results

### 3.1. Patient and Disease Characteristics

**Figure 1:**
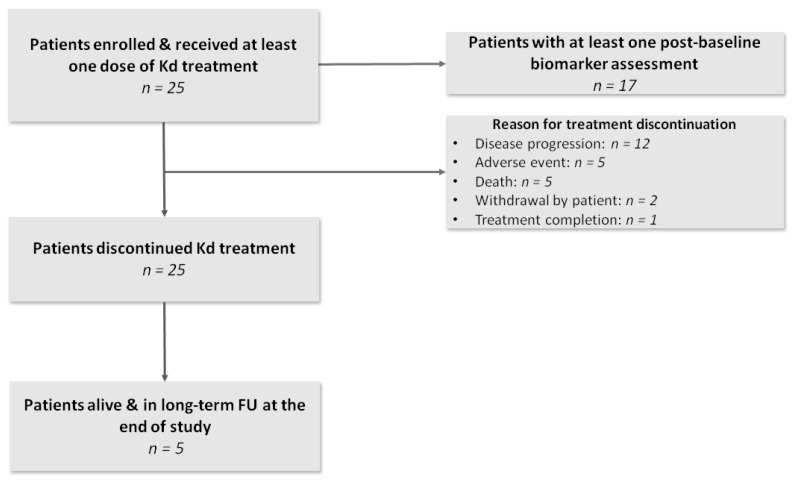
Study flowchart. Patients who discontinued study treatment were followed up for vital status every 4 weeks (long-term follow up phase) unless informed consent was withdrawn.

A total of 25 patients were enrolled between 14 December 2017 and 29 May 2019. All patients received at least one dose of Kd. A total of 17 patients had at least one biomarker assessment post-baseline evaluation (Figure 1).

Table 1 summarizes the characteristics of included patients, overall and according to the emergence of a new SRE during the study period. Approximately half of them were males (48.0%); their median age was 67.5 years, the median time since MM diagnosis was 4.3 years and the median number of previous lines of therapy was 3 (range: 1–8). A total of 14 patients (56%) were refractory to their last line of therapy before Kd initiation and 19 patients (76%) had previously received bisphosphonates. In order to evaluate Kd effects on SREs and bone metabolism, no patient received bisphosphonates or denosumab during the study period.

At baseline, ECOG performance status was 0 for more than half of the patients (*n* = 13, 52%). The vast majority of patients had new osteolytic bone lesions at study entry (time of progression): 21/25 (84%). The number of new lytic bone lesions at baseline was 1–3, 4–10 and >10 in 24%, 28% and 32% of patients, respectively (Table 1). In the majority of patients, the assessment of bone disease was performed with low-dose whole-body computed tomography (LDWBCT) (*n* = 18, 72%), whereas five patients (20%) underwent conventional CT scans, one MRI and one PET/CT scan.

The patients received a median of four (range: 1–18) cycles of treatment with Kd. The median duration of exposure to study treatment was 3.5 (range 0.3–16.6) months. At the end of the study, all patients had discontinued treatment, mainly due to disease progression (*n* = 12, 48%), whereas five patients remained at long-term follow-up (Figure 1). Overall, 11 patients showed a partial response (PR) or better [overall response rate (ORR) = 44%]. Seven patients (28%) presented a deep response including six with very good partial response (VGPR) and one with stringent complete response (sCR). Interestingly, the depth of response was not associated with any of the observed alterations in serum markers of bone metabolism.

### 3.2. Incidence of SREs during Treatment with Kd

During Kd treatment, seven patients (28%) presented with a new SRE. More specifically, six patients (24%) developed pathological fractures (all of them in the spinal vertebrae), four patients (16%) were diagnosed with spinal cord compression and two patients (8%) received radiotherapy to bone. Among patients with at least one SRE, the median (range) number of SREs was 2 (1–3). No significant differences were observed among patients with new SREs during the study compared with those without SREs in terms of baseline characteristics (Table 1).

### 3.3. Effects of Kd on Bone Metabolism

#### 3.3.1. Indices of Bone Remodeling in RRMM Patients at Baseline Compared to Controls

Baseline biomarker levels of patients (*n* = 24) were compared with age- and sex-matched controls (*n* = 48). Patients with RRMM had significantly lower median levels of markers of bone formation bALP (10.9 versus 20.5 μg/L among controls, *p* < 0.001) and OC (9.2 versus 18.9 ng/mL, *p* < 0.001), along with significantly increased median levels of markers of bone resorption CTX (0.7 versus 0.3 ng/mL, *p* < 0.001) and TRACP-5b (3.4 versus 1.0 U/L, *p* < 0.001), as well as increased levels of osteoclast regulators including RANKL (0.3 versus 0.1 pmol/L, *p* = 0.001), activin-A (652 versus 388 pg/mL, *p* < 0.001) and CCL3 (77.8 versus 10.8 ng/mL, *p* < 0.001). Patients also had increased levels of the osteoblast inhibitors Dkk-1 (41.6 versus 22.3 pmol/L, *p* < 0.001) and sclerostin (47.6 versus 22.4 pmol/L, *p* < 0.001) compared to controls.

#### 3.3.2. Bone Resorption and Bone Formation

Regarding bone resorption, all patients experienced clinically relevant (≥30%) reduction in CTX and TRACP-5B in at least one of the studied time points. Significant decreases in both CTX and TRACP-5b values were observed as early as 2 months and sustained for at least 10 months post-treatment initiation with Kd (Table 2). A significant negative effect of Kd therapy over time was observed for both CTX and TRACP-5B values (*p*-value < 0.001 for both).

Regarding bone formation, almost all patients showed clinically relevant increases (≥30%) in OC at 6 and 12 months post-treatment initiation, whereas 63% of patients showed similar increases in P1NP at 4 and 8 months post-Kd initiation. A median percent change >30% from baseline was noted at 8 months for bALP. A statistically significant absolute change in the levels of biomarkers of bone formation was shown only for OC at 6 (*p* = 0.030) and 8 months (*p* = 0.033) of treatment with Kd (Table 2). Although the median values of bALP, OC and P1NP increased over time compared to baseline, a positive effect of Kd treatment over time was shown for OC (*p* = 0.011) and P1NP (*p* = 0.008), but not for bALP (*p* = 0.529).

#### 3.3.3. Osteoclast Regulators and Osteoblast Inhibitors

There was a significant reduction in the serum RANKL, RANKL/OPG ratio and activin-A post-treatment initiation, which occurred as early as at 2 months of treatment with Kd and remained evident for at least 10 months (Table 2, Figures S2 and S3). There was a significant negative effect of Kd over time for all these biomarkers (*p* < 0.001). At 8–12 months post-treatment, 85.7–100% of patients had clinically relevant reductions (≥30%) in both RANKL and the RANKL/OPG ratio. Although a clinically relevant reduction in CCL3 levels was evident at 8 and 10 months post-Kd initiation, the absolute changes in the biomarker levels did not reach statistical significance at any time point (Table 2, Figures S2 and S3). A marginal effect of time on Kd treatment on reducing CCL3 levels was observed (*p* = 0.059).

A clinically relevant reduction in Dkk-1 was observed at 6, 8 and 10 months post-treatment initiation (Table 2, Figures S2 and S3) and it was significant over accumulating time on treatment with Kd (*p* < 0.001). The greatest proportion of patients with a reduction in Dkk1 levels ≥30% was observed at 8 and 12 months post-treatment onset (87.5% and 100%, respectively). The greatest proportion of patients with a reduction in SOST levels ≥30% was observed at 8 months post-treatment onset (75.0%). However, the absolute changes in the biomarker levels did not reach statistical significance in any time point (Table 2, Figures S2 and S3).

#### 3.3.4. Subgroup Analyses

Detailed subgroup analyses evaluating the association between baseline clinical features and markers of bone metabolism in each examined time point are provided in Tables S1–S16. The low number of patients in each subgroup prevents the establishment of statistical rigor in the results. Furthermore, the markers of bone metabolism were not affected by myeloma disease progression, since no significant differences were observed at all time points (Table S17).

### 3.4. TtNT, PFS and OS

**Figure 2:**
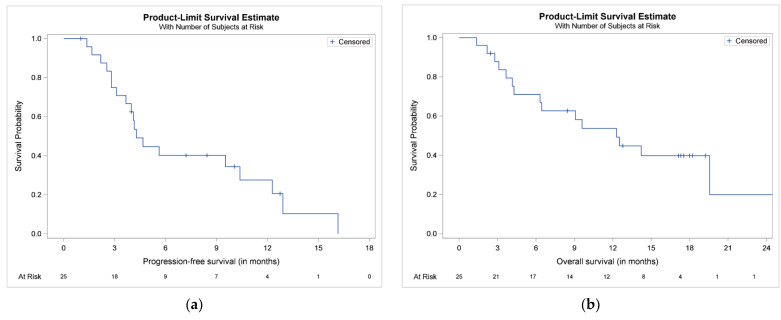
Kaplan–Meier plots of (**a**) progression-free survival (PFS) and (**b**) overall survival (OS) of the 25 patients included in the CarMMa study.

Overall, 12 patients (48%) progressed on Kd and 7 died without prior documented disease progression. The median (95% CI) TtNT was 5.69 (3.98–12.93) months. The median (95% CI) PFS was 4.28 (3.1–10.38) months (Figure 2a). The estimated 6- and 12-month PFS rates were 40.18% (95% CI: 20.7, 58.99) and 27.55% (95% CI: 10.33, 48.09), respectively. A total of 16 patients (64%) died during the study period. The median (95% CI) OS was 12.28 (4.28, 25.23) months (Figure 2b). The estimated 12- and 24-month OS rates were 53.77% (95% CI: 32.13, 71.24) and 19.91% (95% CI: 1.78, 52.38), respectively.

### 3.5. Safety Evaluation

Adverse events of any grade were reported in all patients (100%). Among 21 (84.0%) patients who experienced at least one non-serious adverse event, the most commonly reported were anemia (*n* = 8, 32.0%), pyrexia (*n* = 6, 24.0%) and lower respiratory tract infection (*n* = 6, 24.0%). In total, 11 (44.0%) patients experienced at least one serious adverse event including sepsis, anemia, thrombocytopenia, thrombotic thrombocytopenic purpura, deep vein thrombosis, atrial fibrillation, cardiac failure, pulmonary oedema, respiratory tract infection, pyelonephritis and second primary malignancy.

## 4. Discussion

This prospective study is the first study in the literature which evaluated, in depth, the effects of Kd combination on bone health in patients with advanced RRMM in a real-world setting. Myeloma bone disease is a common feature for patients with MM; more than 70% present with osteolytic bone disease at diagnosis, whereas bone pain constitutes a very frequent presenting symptom leading to MM diagnosis [2]. In our study, 36% of the patients had a previous history of SREs at the time of Kd initiation. This is in line with a real-world retrospective study of 343 patients with MM, which showed that 34% of them presented with SREs during a median follow-up of 25.7 months since diagnosis [24]. Interestingly, most SREs are reported during the first year from diagnosis both in clinical trials and real-world reports [24,25]. The majority of patients (72%) in our study, who had advanced myeloma, did not experience any new SRE during our study period, whereas a beneficial effect on markers of bone metabolism became evident. Although proteasome inhibitors exert a favorable effect on bone health, the inclusion of proteasome inhibitors in the anti-myeloma treatment did not alter the incidence of SREs in a retrospective study of the Mayo Clinic. More specifically, for patients receiving third-line therapy, the incidence rate of SREs within 1 year of third-line treatment initiation was 265.2 per 100 person-years for proteasome inhibitor + immunomodulatory regimens, 93.1 per 100 person-years for proteasome inhibitor without immunomodulatory regimens and 81.6 per 100 person-years for non-proteasome inhibitor regimens [24]. To our knowledge, there is no information in the literature for MM patients who have received a median of three previous lines of therapy (as in our study) regarding the effect of any given regimen on SREs and bone metabolism. In our study, the improvement in the values of bone indices became more pronounced over time for most biomarkers. Interestingly, a reduction in markers of bone resorption and osteoclast activity may predict for a reduced risk for SREs in patients with MM [26,27,28].

Kd significantly reduced bone resorption and favored bone formation. This was at least partially attributed to a reduction in both osteoclast regulators and osteoblast inhibitors. Interestingly, changes in markers of bone remodeling were not associated with the depth of myeloma response, which has been previously reported with regimens that do not contain proteasome inhibitors [29]. Therefore, it seems that Kd has an important anabolic effect on the bones, apart from the anti-myeloma cytotoxic activity [30].

Our results are in line with the preliminary results of a phase 2 study evaluating bone metabolism in patients with RRMM who received single-agent carfilzomib. In that study, only 4 markers of bone turnover were evaluated (CTX, TRACP-5b, P1NP and OC) and 10 patients with a median of 2 prior lines of therapy were included in the primary analysis. Similar to our results, carfilzomib resulted in a significant decrease in serum markers of bone resorption (CTX, TRACP-5b). However, no significant changes were observed for markers of bone formation (P1NP, OC), although patients who achieved a PR or better had an interesting increase of more than 25% [31]. Indeed, we also showed that markers of bone resorption (CTX, TRACP-5b) are sensitive to changes as early as at the first 2 months post-treatment initiation with Kd. On the contrary, clinically relevant changes from baseline values became evident only after several months of treatment for markers of bone formation (4 months for P1NP, 6 months for OC, 8 months for bALP). In another retrospective analysis of 67 patients enrolled in clinical trials evaluating biweekly carfilzomib at 20 mg/m^2^, an increase in total ALP was associated with subsequent disease response [20]. In this case, the reduction in myeloma burden may enable the homeostasis of the bone microenvironment and the formation of new bone [32].

Carfilzomib has a multifaceted anabolic role on bone. In vitro studies have previously shown that carfilzomib favors bone formation over resorption by inhibiting osteoclast generation and promoting osteoblast formation and mineralization of the bone matrix [33]. A more detailed in vitro study showed that carfilzomib mainly inhibits the differentiation of immature osteoclasts to mature osteoclasts, whereas it does not interfere with the formation of the osteoclast sealing zone. The addition of a novel inhibitor of Bruton’s tyrosine kinase CC-292 to carfilzomib had a synergistic effect and resulted in the inhibition of sealing zone formation and osteoclast maturation [34]. The osteoclast sealing zone is a specialized osteoclast–matrix adhesion structure, which delineates the resorption area of the bone and is essential for osteoclast function [35].

Furthermore, we found that carfilzomib induced an early reduction in the RANKL/OPG ratio, which downregulates osteoclast formation. Patients with MM present with an increased RANKL/OPG ratio, which is associated with both an increased burden of myeloma bone disease and poor patient survival [36]. Carfilzomib has been shown to prevent proteasomal degradation of histone deacetylase 4 (HDAC4), which inhibits the RANKL expression mediated by the parathyroid hormone (PTH). OPG expression is not significantly affected by carfilzomib and, therefore, the RANKL/OPG ratio in osteoblasts is reduced and osteoclastogenesis is suppressed [37].

In our study, Kd resulted also in an early reduction in activin-A levels. This effect may be mainly attributed to carfilzomib, since treatment with lenalidomide and dexamethasone did not result in a reduction in activin-A levels in a previous study [38]. MM cells induce the activin-A secretion from stromal cells, and increased activin-A levels have been associated with extensive myeloma bone disease and worse patient survival [38,39].

In addition to the above, we also found clinically relevant decreases in the levels of the pro-inflammatory chemokine CCL3 (MIP-1a) at 8 months from Kd initiation. Patients with MM who present with increased levels of CCL3 have more extensive bone disease due to an upregulation of osteoclast differentiation and worse survival [40,41]. CCL3 may impair the function of osteoblasts by downregulating the osteogenic transcription factor osterix, which leads to decreased levels of OC, as well [42,43]. The regulation of osteoclasts and osteoblasts is interrelated in the bone marrow milieu [32,44].

Importantly, carfilzomib induces the differentiation of osteoprogenitor cells and mesenchymal stem cells into mature osteoblasts [45,46]. More specifically, carfilzomib stabilizes β-catenin and induces β-catenin/TCF transcriptional activity independently of the Wnt signaling cascade. This in turn upregulates alkaline phosphatase activity and induces mineralization of the bone matrix and deposition of calcium crystals [45]. Furthermore, carfilzomib restores physiological deactivation of Notch1, which favors the differentiation of mesenchymal stem cells and osteogenesis [47].

Carfilzomib may also target the osteoblast proteasome and promote osteoblast survival and bone formation [46,48]. Proteasome inhibitors, and especially the first-in-class bortezomib, inhibit osteoclast function but also enhance osteoblast activity. In myeloma patients, bortezomib increases bone mineral density [13] and bone volume [49] and leads to the healing of bone lesions [12], which happens very rarely in myeloma. It seems that proteasome inhibition induces endoplasmic reticulum stress-related signaling pathways. The inositol-requiring protein 1α- X box binding protein 1 (IRE1α- XBP1s) signaling cascade is considered as a key effector of osteoblast differentiation which is mediated by proteasome inhibition. XBP1s along with activating transcription factor 4 (ATF4), which is also activated during the endoplasmic reticulum stress, upregulates the transcription of osteogenic differentiation-related genes and promotes osteoblast formation and function [46].

In our study, we showed clinically relevant reductions in the osteoblast inhibitors Dkk-1 and SOST, which were more pronounced at 8 months post-treatment initiation with Kd. Significant decreases in the levels of Dkk-1 mRNA with carfilzomib have been also demonstrated in cell cultures of MG63 cells [45]. Dkk-1 is a soluble, extracellular antagonist of the Wnt signaling pathway, which is implicated in the regulation of bone formation [50]. Patients with MM have increased levels of Dkk-1, which ultimately suppress osteoblast differentiation and favor the development of osteolytic bone disease [51,52]. Regarding SOST, it is expressed by osteocytes and myeloma cells and it is a negative regulator of the Wnt pathway and bone formation [53,54,55]. Increased SOST levels in patients with MM have been associated with deregulated bone turnover and advanced disease stage [56]. Increased Dkk-1 levels upregulate SOST expression and they synergistically prevent bone formation [57]. Interestingly, treatment with an antiresorptive agent (zoledronic acid) and an anabolic drug (anti-SOST antibody) increased bone strength in preclinical myeloma models and may provide a rationale for relevant clinical studies [58,59].

The main limitation of our study lies in the relative, small number of recruited patients. Although the study had an adequate statistical power for the primary outcome, sub-analyses may have been underpowered due to the small number of patients in each subgroup. Although all patients were followed for their vital status until the end of the study, bone indices were evaluated only during treatment with Kd. Therefore, this is an additional reason why the number of patients is low in the subgroup analyses of markers of bone metabolism, especially in the later time points. Furthermore, the time and duration of previous bisphosphonate therapy before the initiation of Kd might impact bone-related outcomes. Last but not least, it would be interesting to perform serial bone mineral density studies during Kd and evaluate their findings taking into consideration the respective changes in markers of bone metabolism.

## 5. Conclusions

In summary, the majority of patients with advanced myeloma did not experience SREs during treatment with Kd. Kd reduced bone resorption and increased bone formation in these patients who were treated outside of a clinical trial. These changes were, at least partially, due to a reduction in the RANKL/OPG ratio, CCL-3, Dkk-1 and activin-A. They were clinically relevant in the majority of patients and appeared to be independent of treatment response. Our results suggest a beneficial effect of carfilzomib on bone metabolism of patients with RRMM, which deepens over time, even in the absence of bone-targeted agents (zoledronic acid or any other bisphosphonate or denosumab). Restoring bone health by assisting the bone microenvironment to return to homeostasis in patients with MM is essential in order to improve the quality of life. The combination of carfilzomib with antiresorptive and targeted agents will further enhance bone strength and ameliorate patient outcomes.

## Figures and Tables

**Table 1:** Characteristics of the included patients, overall and according to the emergence of skeletal-related events (SREs) during treatment with Kd.

Variables	Overall (*n* = 25)	SRE during the Study Interval (*n* = 7)	No SRE during the Study Interval (*n* = 18)	*p*-Value ^a^
Age at enrollment (years),	67.5 (53.2–76.8)	67.5 (56.1–76.8)	67.7 (53.2–76.2)	0.739
Age at diagnosis (years)	64.0 (41.1–73.9)	66.4 (45.5–73.3)	63.2 (41.1–73.9)	0.785
Time from diagnosis (years)	4.3 (0.4–19.4)	2.0 (0.4–10.6)	4.4 (0.9–19.4)	0.138
Male sex	12 (48.0%)	4 (57.1%)	8 (44.4%)	0.673
Greek ethnicity	25 (100%)	7 (100%)	18 (100%)	
Women, postmenopausal	13 (152.0%)	3 (42.9%)	10 (55.6%)	0.236
BMI (kg/m^2^)	26.4 (17.7–34.3)	29.8 (23.1–34.3)	25.6 (17.7–33.3)	0.127
ECOG PS at Kd initiation				
0	13 (52.0%)	3 (42.9%)	10 (55.6%)	0.252
1	7 (28.0%)	1 (14.3%)	6 (33.3%)	
2 or higher	5 (20.0%)	3 (42.9%)	2 (11.1%)	
ISS at diagnosis				
I	8 (32.0%)	0 (0%)	8 (44.4%)	0.092
II	9 (36.0%)	3 (42.9%)	6 (33.3%)	
III	8 (32.0%)	4 (57.1%)	4 (22.2%)	
R-ISS at diagnosis				
I	7 (28.0%)	0 (0%)	7 (38.9%)	0.159
II	12 (48.0%)	5 (71.4%)	7 (38.9%)	
III	6 (24.0%)	2 (28.6%)	4 (22.2%)	
ISS at Kd initiation				
I	9 (36.0%)	2 (28.6%)	7 (38.9%)	0.295
II	8 (32.0%)	1 (14.3%)	7 (38.9%)	
III	8 (32.0%)	4 (57.1%)	4 (22.2%)	
R-ISS at Kd initiation				
I	6 (24.0%)	1 (14.3%)	5 (27.8%)	0.188
II	12 (48.0%)	2 (28.6%)	10 (55.6%)	
III	7 (28.0%)	4 (57.1%)	3 (16.7%)	
Prior ASCT	14 (56.0%)	3 (42.9%)	11 (61.1%)	0.656
Prior radiotherapy	7 (28.0%)	4 (57.1%)	3 (16.7%)	0.066
Prior lines of therapy	3.0 (1.0–8.0)	3.0 (1.0–5.0)	3.5 (1.0–8.0)	0.294
Refractoriness to:				
PI	11 (44.0%)	4 (57.1%)	7 (38.9%)	0.656
IMiD	16 (64.0%)	5 (71.4%)	11 (61.1%)	>0.999
PI and IMiD	10 (40.0%)	3 (42.9%)	7 (38.9%)	>0.999
Pomalidomide	5 (20.0%)	2 (28.6%)	3 (16.7%)	0.597
Daratumumab	5 (20.0%)	2 (28.6%)	3 (16.7%)	0.597
Last line of therapy	14 (56.0%)	6 (85.7%)	8 (44.4%)	0.090
Prior use of bisphosphonates (during the last prior therapy)	19 (76.0%)	6 (85.7%)	13 (72.2%)	0.637
Prior use of proteasome inhibitor	22 (88.0%)	7 (100.0%)	15 (83.3%)	0.534
No bone disease at diagnosis	9 (36.0%)	2 (28.6%)	7 (38.9%)	>0.999
Lytic bone lesions at Kd initiation				
None	4 (16.0%)	1 (14.3%)	3 (16.7%)	0.466
1–3	6 (24.0%)	1 (14.3%)	5 (27.8%)	
4–10	7 (28.0%)	1 (14.3%)	6 (33.3%)	
More than 10	8 (32.0%)	4 (57.1%)	4 (22.2%)	
Prior history of SREs	9 (36.0%)	3 (42.9%)	6 (33.3%)	0.673

BMI: bone mineral density; ECOG PS: Eastern Cooperative Oncology Group Performance Status; (R)ISS: (Revised) International Staging System; ASCT: autologous stem cell transplant; PI: proteasome inhibitor; IMiD: immunomodulatory drug; Kd: carfilzomib–dexamethasone; SREs: skeletal-related events. Quantitative variables are presented as median (range) and qualitative variables are presented as *n* (%).^a^ Mann–Whitney U test or Fisher’s exact test, as applicable.

**Table 2:** Median (Q1-Q3) levels of biomarkers of bone metabolism at baseline and at 2, 4, 6, 8, 10 and 12 months post-treatment initiation and respective percent changes from baseline.

Variables	Baseline	2 Months	4 Months	6 Months	8 Months	10 Months	12 Months
**bALP (μg/L)**							
*n*	25	17	11	9	8	7	2
Median biomarker value (Q1, Q3)	10.9 (9.1, 11.7)	12.1 (9.1, 15.4)	11.6 (9.1, 14.1)	13.6 (8.1, 14.8)	16.0 (6.2, 17.4)	15.0 (7.0, 18.1)	17.1 (14.5, 19.7)
Median percent change from baseline (Q1, Q3)		12.1 (−9.4, 29.5)	3.5 (−19.7, 37.8)	16.1 (−36.3, 30.6)	37.7 (−45.1, 67.0)	27.8 (−38.4, 58.0)	56.6 (23.7, 89.5)
*p*-value for absolute change ^a^		0.487	0.597	0.825	0.963	0.696	NA
**OC (ng/mL)**							
*n*	25	17	11	9	8	7	2
Median biomarker value (Q1, Q3)	9.2 (5.5, 11.3)	10.5 (8.8, 14.1)	12.4 (9.9, 19.2)	13.9 (11.1, 18.9)	15.9 (7.3, 23.8)	16.8 (3.8, 19.7)	17.1 (13.3, 20.8)
Median percent change from baseline (Q1, Q3)		23.4 (19.0, 65.2)	64.4 (35.5, 242.2)	89.7 (39.2, 169.3)	61.2 (33.0, 216.9)	71.7 (49.6, 167.4)	65.8 (44.8, 86.7)
*p*-value for absolute change ^a^		0.257	0.099	0.030	0.033	0.203	NA
**P1NP (pg/mL)**							
*n*	25	17	11	9	8	7	2
Median biomarker value (Q1, Q3)	542.2 (294.8, 746.4)	384.9 (226.3, 775.3)	490.2 (411.6, 777.5)	442.8 (419.7, 789.0)	884.9 (461.1, 2072.1)	652.0 (447.6, 2567.2)	992.5 (701.3, 1283.7)
Median percent change from baseline (Q1, Q3)		7.9 (−30.8, 21.9)	38.4 (−34.1, 105.9)	20.6 (−41.5, 33.7)	42.4 (24.9, 110.4)	92.8 (11.8, 173.8)	58.2 (20.2, 96.2)
*p*-value for absolute change ^a^		0.918	0.437	0.469	0.059	0.061	NA
**CTX (ng/mL)**							
*n*	25	17	11	9	8	7	2
Median biomarker value (Q1, Q3)	0.7 (0.3, 0.9)	0.4 (0.2, 0.6)	0.3 (0.2, 0.5)	0.2 (0.2, 0.4)	0.1 (0.1, 0.4)	0.2 (0.1, 0.3)	0.3 (0.2, 0.4)
Median percent change from baseline (Q1, Q3)		−31.3 (−43.0, −15.0)	−48.4 (−63.5, 42.8)	−43.5 (−64.6, −31.4)	−59.9 (−86.1, −48.0)	−63.7 (−74.9, −31.2)	−74.2 (−79.7, −68.6)
*p*-value for absolute change ^a^		0.048	0.054	0.029	<0.001	0.001	NA
**TRACP-5B (U/L)**							
*n*	25	17	11	9	8	5	2
Median biomarker value (Q1, Q3)	3.4 (1.7, 4.0)	1.9 (1.0, 2.1)	1.2 (0.8, 2.0)	1.3 (1.1, 1.9)	1.0 (0.9, 1.1)	0.9 (0.9, 0.9)	1.3 (0.9, 1.8)
Median percent change from baseline (Q1, Q3)		−35.3 (−49.7, −9.5)	−48.6 (−66.0, −21.6)	−22.8 (−66.3, −17.9)	−64.0 (−70.2, −52.9)	−72.1 (−73.6, −59.1)	−58.3 (−58.8, −57.7)
*p*-value for absolute change ^a^		0.002	<0.001	0.043	<0.001	<0.001	NA
**RANKL (pmol/L)**							
*n*	25	17	11	9	8	7	2
Median biomarker value (Q1, Q3)	0.3 (0.2, 0.4)	0.2 (0.1, 0.2)	0.1 (0.1, 0.1)	0.1 (0.1, 0.1)	0.1 (0.1, 0.1)	0.1 (0.0, 0.2)	0.1 (0.1, 0.2)
Median percent change from baseline (Q1, Q3)		−47.5 (−52.9, −1.6)	−53.5 (−77.5, 44.9)	−63.2 (−77.0, 3.8)	−71.7 (−84.7, −55.0)	−73.0 (−92.9, −58.3)	−82.8 (−87.0, −78.6)
*p*-value for absolute change ^a^		0.032	0.001	0.001	<0.001	<0.001	NA
**RANKL/OPG ratio**							
*n*	25	17	11	9	8	7	2
Median biomarker value (Q1, Q3)	0.072 (0.000, 0.123)	0.036 (0.000, 0.101)	0.031 (0.000, 0.098)	0.017 (0.000, 0.077)	0.010 (0.011, 0.064)	0.009 (0.000, 0.033)	0.005 (0.00, 0.021)
Median percent change from baseline (Q1, Q3)		−52.2 (−69.2, 10.6)	−60.4 (−86.8, 44.5)	−77.0 (−85.1, 5.3)	−86.9 (−93.5, −48.8)	−84.9 (−94.7, −47.0)	−92.9 (−94.9, −91.0)
*p*-value for absolute change ^a^		0.026	<0.001	<0.001	<0.001	<0.001	NA
**SOST (pmol/L)**							
*n*	25	17	11	9	8	7	2
Median biomarker value (Q1, Q3)	47.6 (38.0, 65.1)	37.2 (29.4, 41.7)	33.2 (25.0, 45.8)	31.8 (25.5, 63.1)	28.0 (22.3, 53.9)	36.9 (20.2, 64.7)	27.8 (20.0, 35.7)
Median percent change from baseline (Q1, Q3)		−24.0 (−39.6, 6.6)	−31.0 (−44.5, −5.6)	−27.4 (−32.0, 25.5)	−36.7 (−48.4, −26.6)	−38.9 (−52.0, 0.5)	−50.8 (−55.3, −46.2)
*p*-value for absolute change ^a^		0.272	0.306	0.869	0.597	0.191	NA
**Dkk1 (pmol/L)**							
*n*	25	17	11	9	8	7	2
Median biomarker value (Q1, Q3)	41.6 (28.2, 63.7)	36.9 (26.9, 62.5)	33.7 (18.5, 58.4)	37.0 (32.0, 49.2)	29.0 (21.5, 32.7)	26.1 (9.1, 31.0)	14.4 (8.4, 20.4)
Median percent change from baseline (Q1, Q3)		−24.0 (−27.8, 3.7)	−21.0 (−58.3, −14.8)	−31.5 (−59.3, −23.8)	−61.4 (−68.6, −39.3)	−64.2 (−82.6, −29.2)	−78.0 (−84.0, −72.0)
*p*-value for absolute change ^a^		0.856	0.393	0.399	0.037	0.005	NA
**Activin-A (pg/mL)**							
*n*	25	17	11	9	8	7	2
Median biomarker value (Q1, Q3)	652.0 (498.6, 903.5)	462.2 (358.2, 538.3)	418.7 (334.5, 519.6)	378.7 (366.9, 504.5)	392.0 (275.4, 488.5)	357.5 (280.5, 422.7)	287.5 (256.8, 318.2)
Median percent change from baseline (Q1, Q3)		−22.7 (−39.9, −5.7)	−37.3 (−63.4, −16.2)	−48.5 (−59.0, −21.1)	−40.2 (−66.9, −30.0)	−58.0 (−61.7, −27.4)	−55.3 (−58.2, −52.4)
*p*-value for absolute change ^a^		0.015	0.007	0.008	0.008	<0.001	NA
**CCL3 (ng/mL)**							
*n*	25	17	11	9	8	7	2
Median biomarker value (Q1, Q3)	77.8 (61.8, 91.6)	70.5 (44.0, 89.4)	68.0 (47.0, 72.0)	62.1 (61.2, 71.1)	58.1 (37.7, 65.2)	50.7 (9.1, 57.9)	34.1 (3.9, 64.3)
Median percent change from baseline (Q1, Q3)		−3.9 (−36.4, 8.7)	−17.8 (−24.4, 43.8)	−17.4 (−29.5, −11.9)	−33.0 (−55.2, −11.7)	−44.5 (−87.5, −21.7)	−55.3 (−94.6, −16.1)
*p*-value for absolute change ^a^		0.849	0.577	0.958	0.668	0.063	NA

N, number of patients; NA, not applicable; Q1, first quartile; Q3, third quartile; ^a^ estimated using a linear repeated measures model with biomarker log-transformed values at each time point as the dependent variable and visit (i.e., cycle) as fixed effect.

## Data Availability

Data available upon request to the corresponding author.

## Supplementary Materials

The following are available online at https://www.mdpi.com/2072-6694/13/6/1257/s1, Figure S1, Biomarker distribution (boxplots) over time, Figure S2, Lineplots of biomarker values per patient to depict changes over time, for all patients with at least one post-baseline assessment, Figure S3, Lineplots of biomarker values per patient to depict changes over time, for patients with at least two post-baseline assessment, Tables S1–S17, Biomarker values at each timepoint.
