# Supplementary material for: Carfilzomib Improves Bone Metabolism in Patients with Advanced Relapsed/Refractory Multiple Myeloma: Results of the CarMMa Study
Source: Cancers (Basel). 2021 Mar 12;13(6):1257. doi: 10.3390/cancers13061257 (PMC7998249; doi:10.3390/cancers13061257)
Supplement: Supplementary file 1 [file cancers-13-01257-s001.zip › cancers-1126248-supplementary-1/Supplemental Figures.docx]

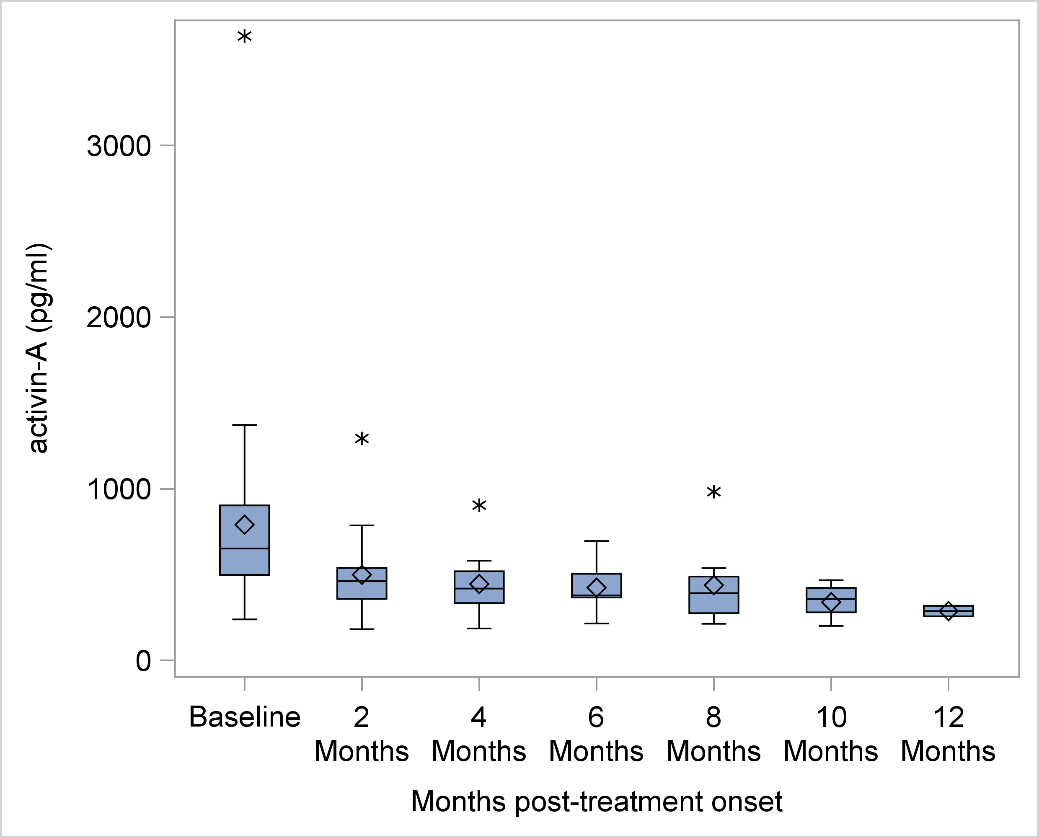

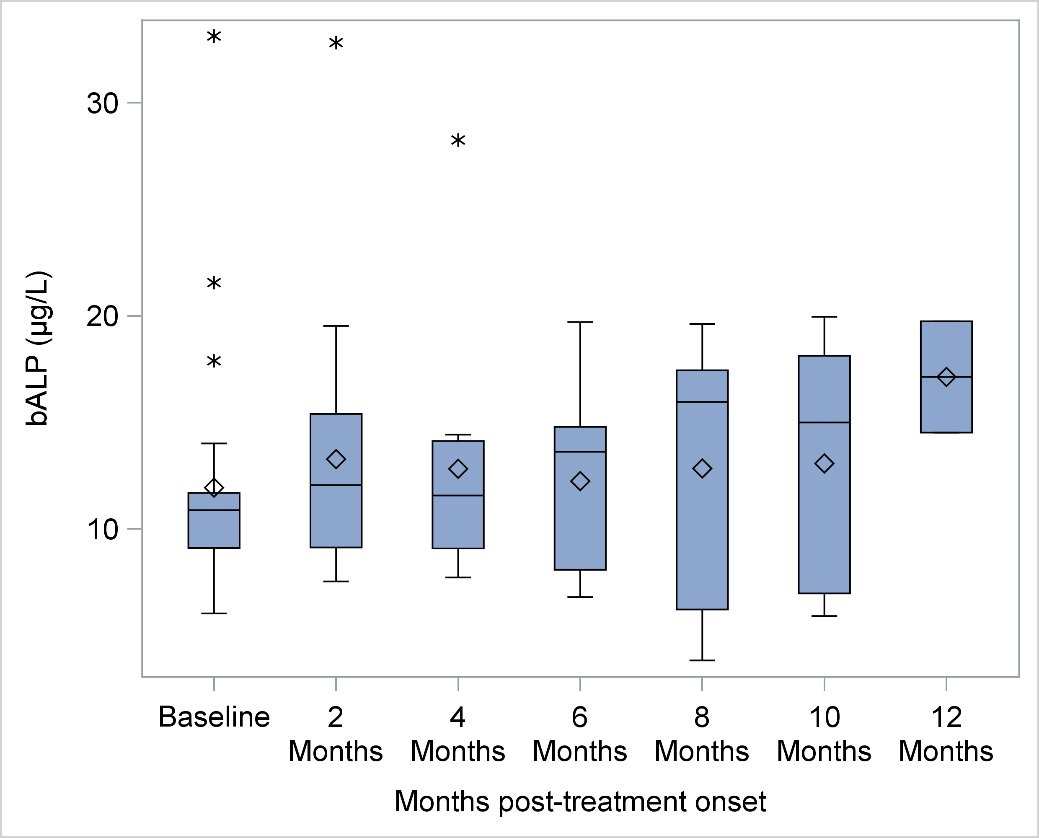

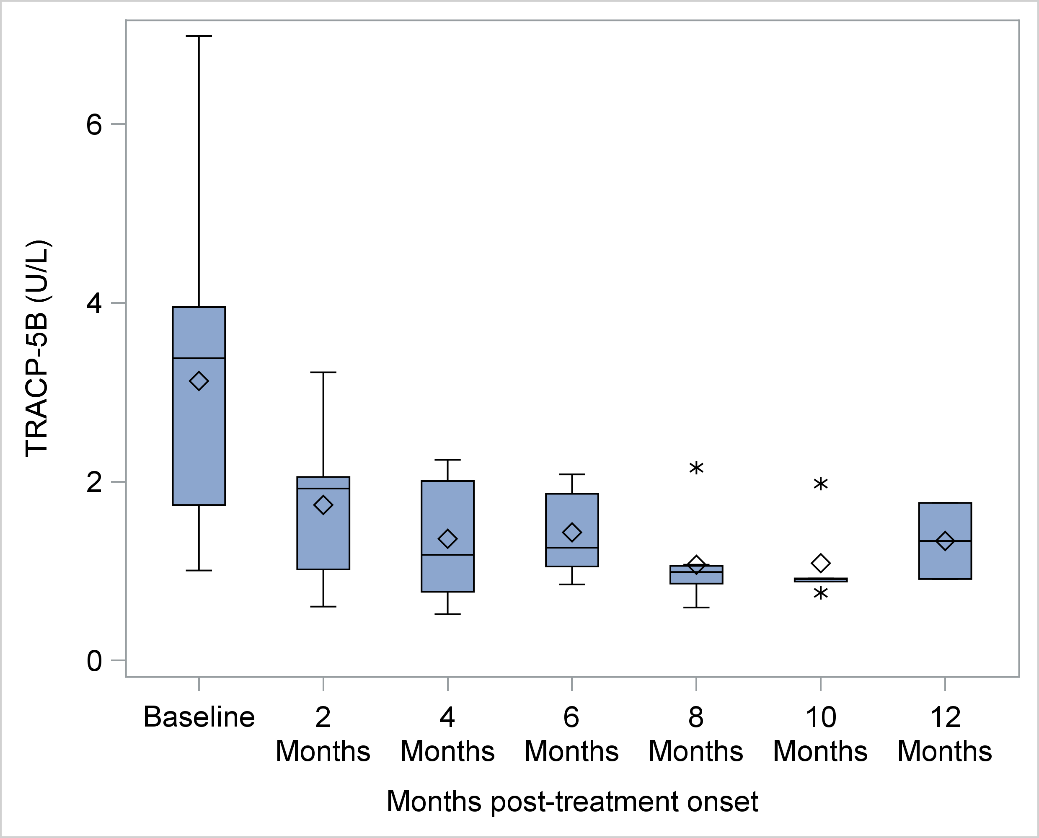

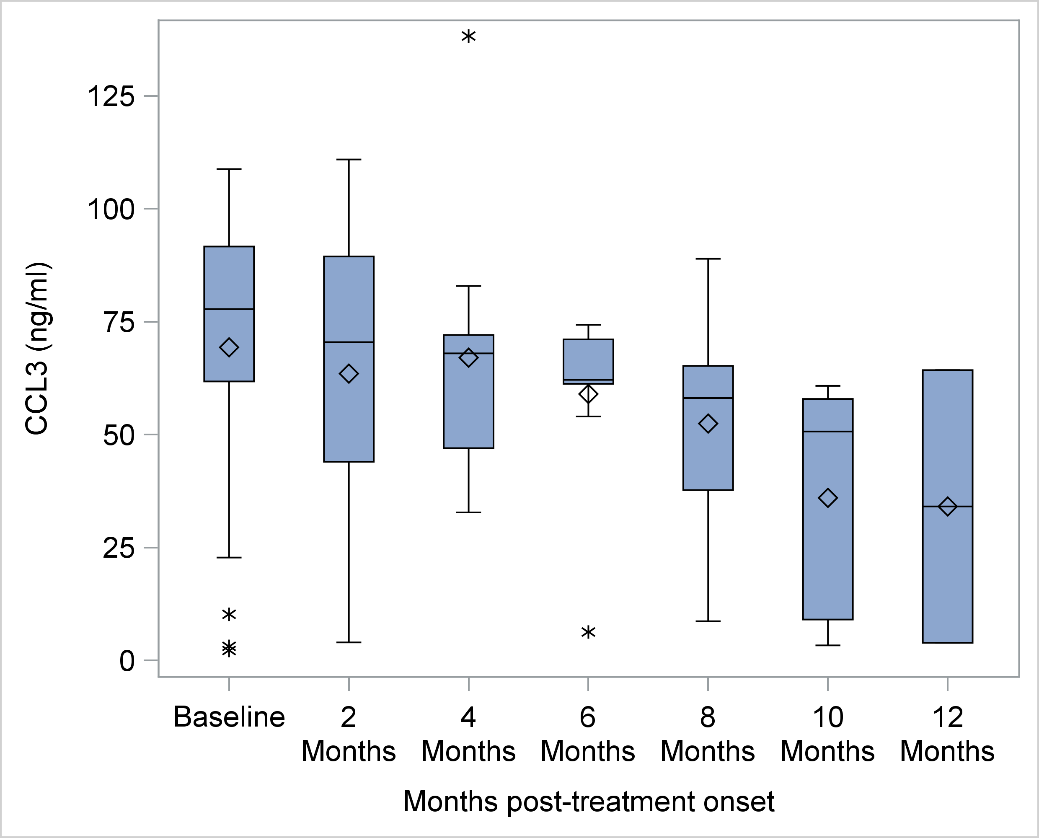

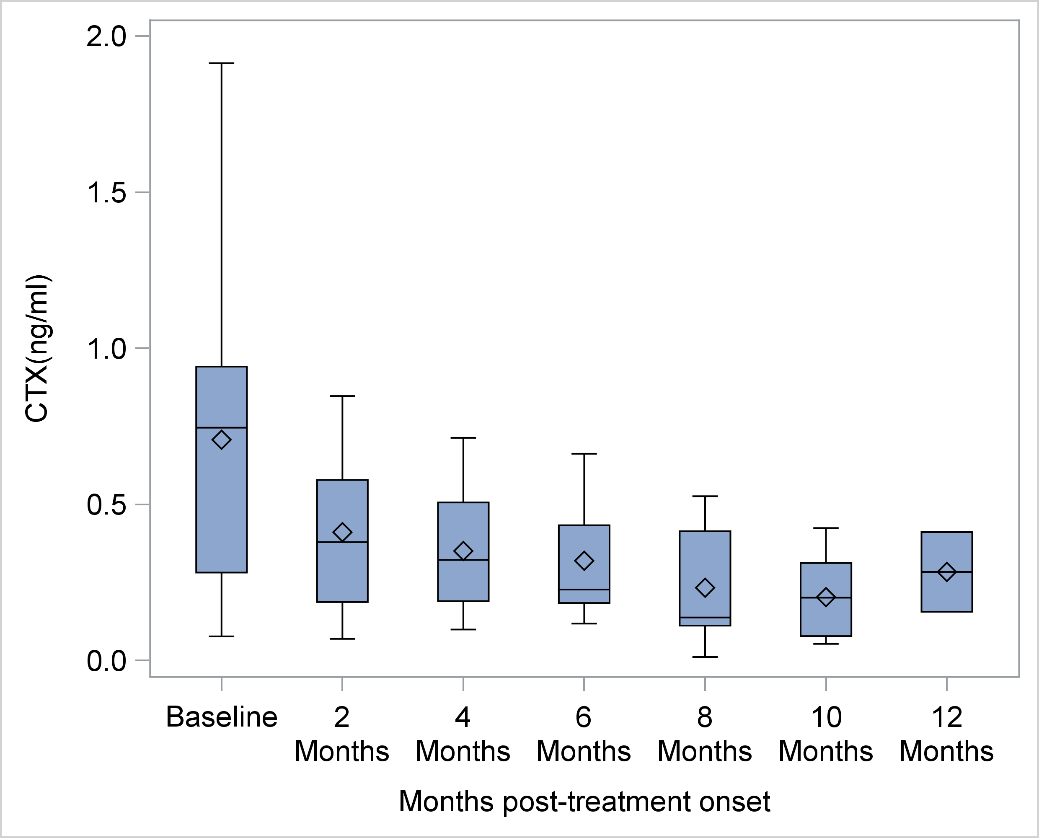

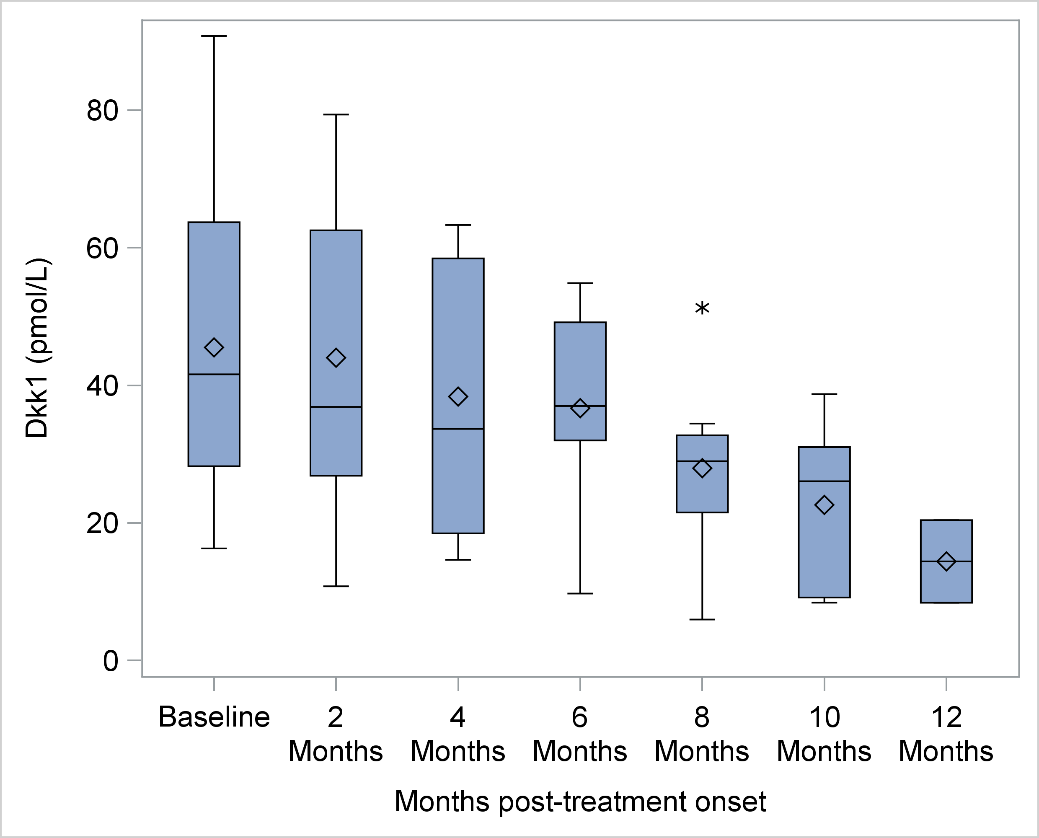

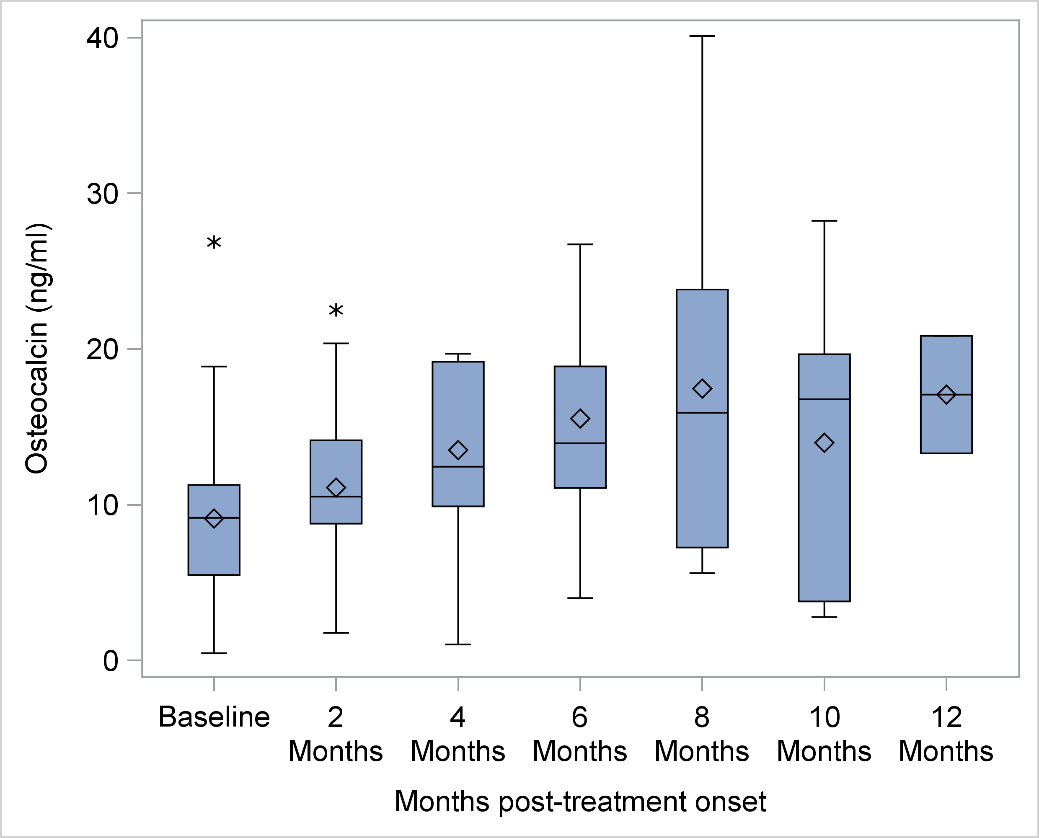

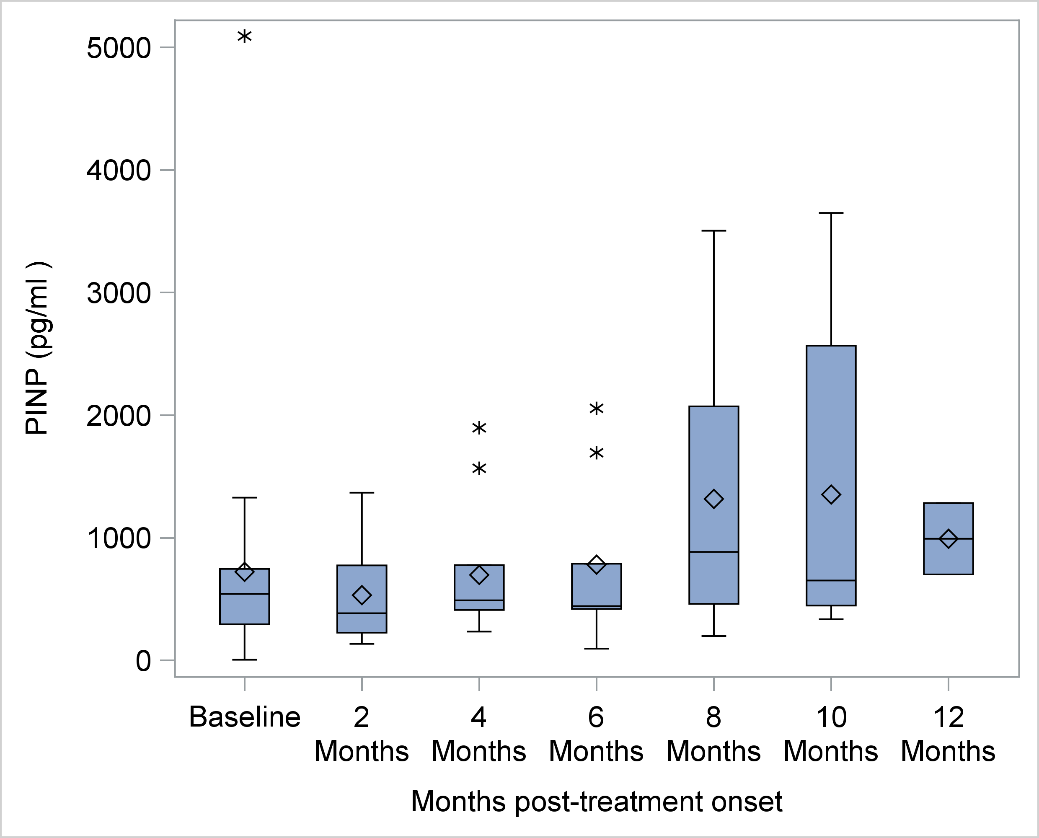

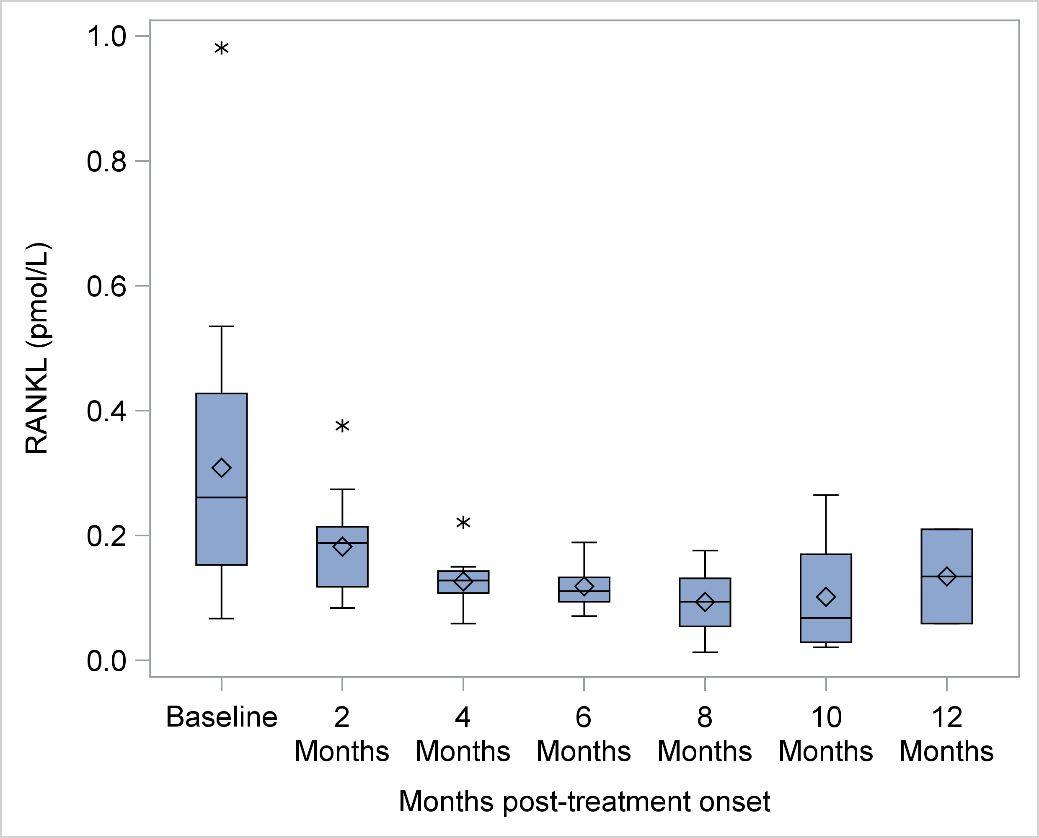

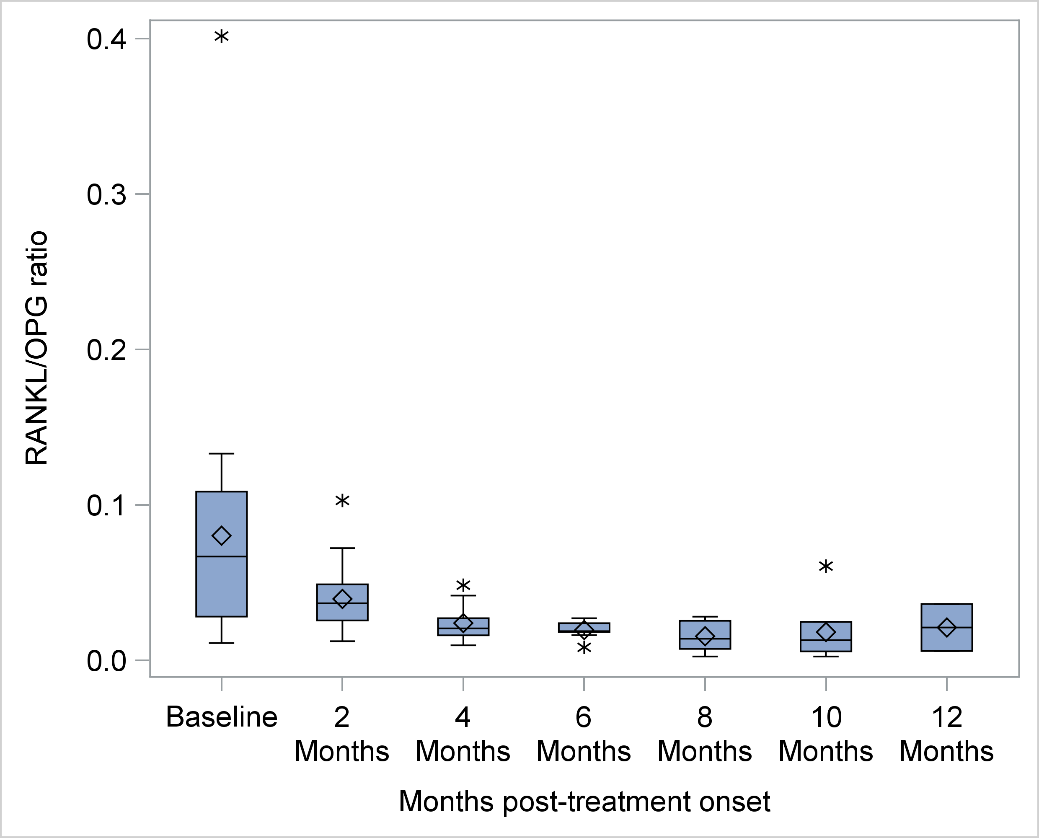

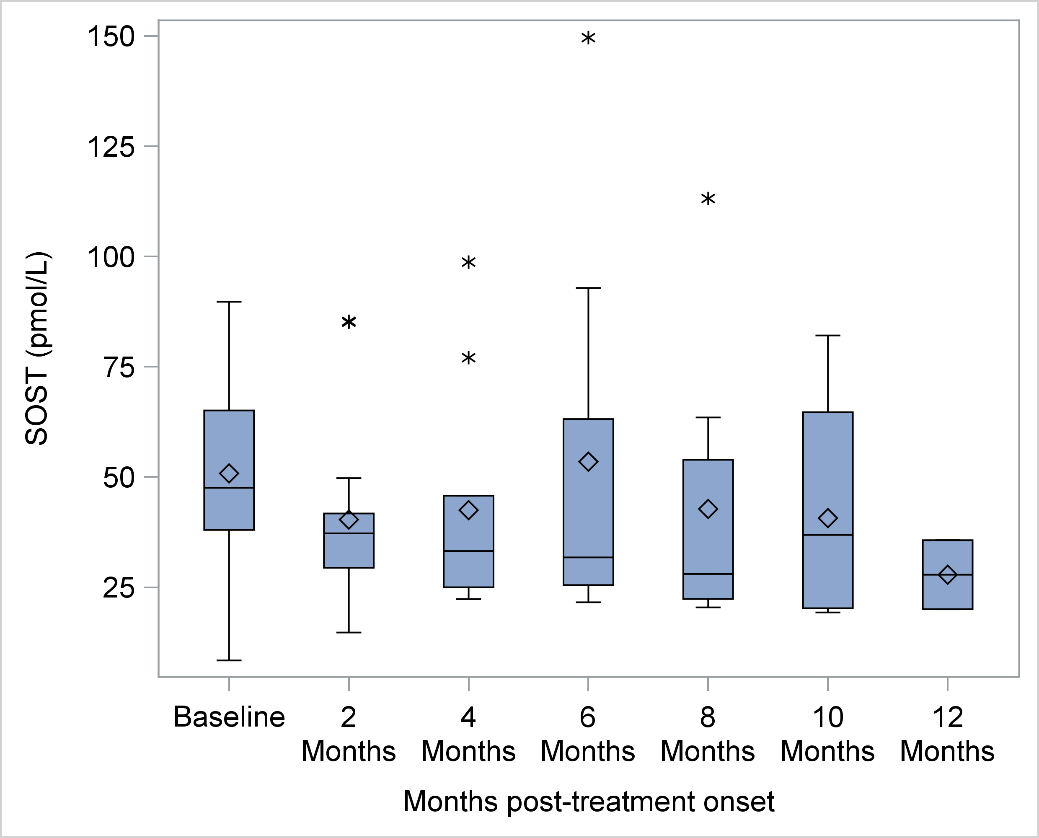


**Figure S1.** Biomarker distribution (boxplots) over time. The box limits represented the 1st and 3rd quartiles (Q1, Q3) while the horizontal line within each box represents the median value. The diamond symbol represents the mean value. The asterisk symbol represents ‘outliers’ (observations more than +/- 1.5 times the Interquartile range). “*” “**” denote extreme values.


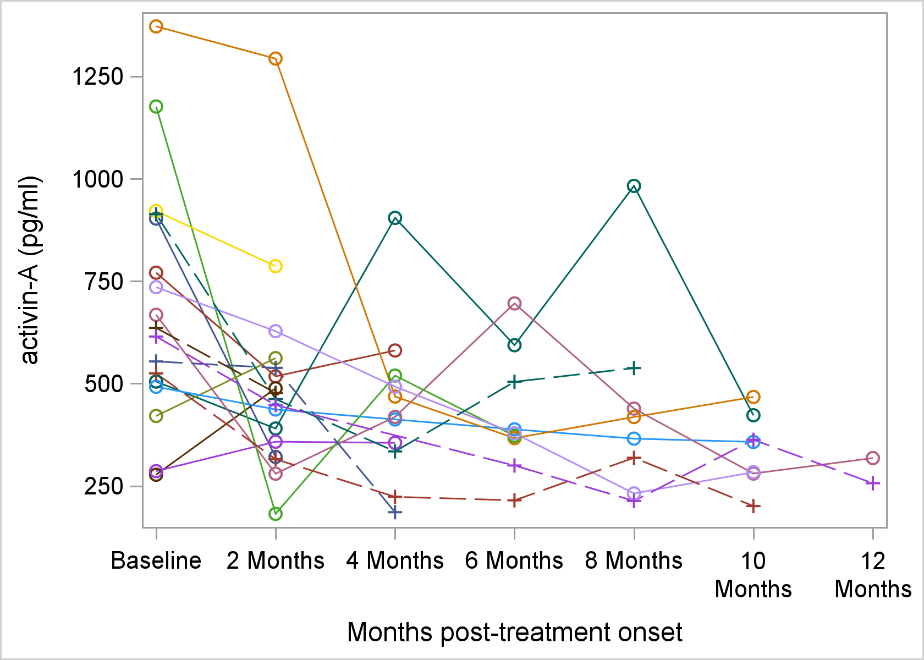

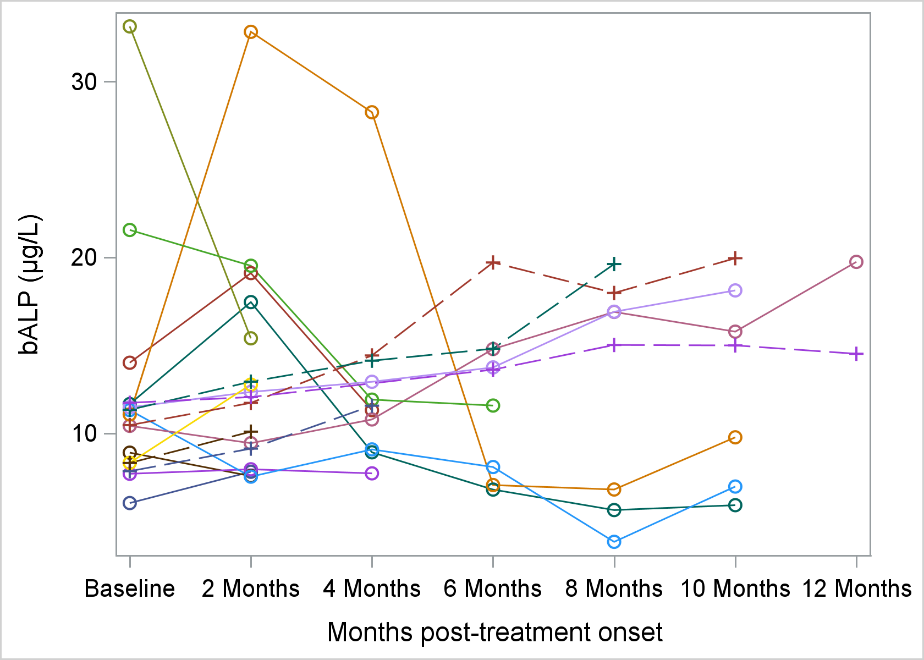

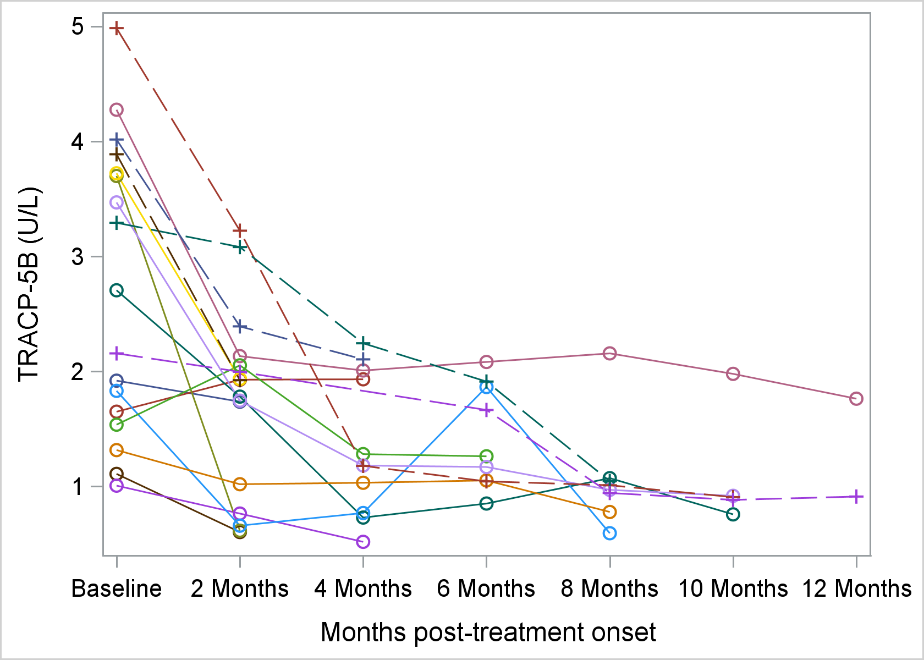

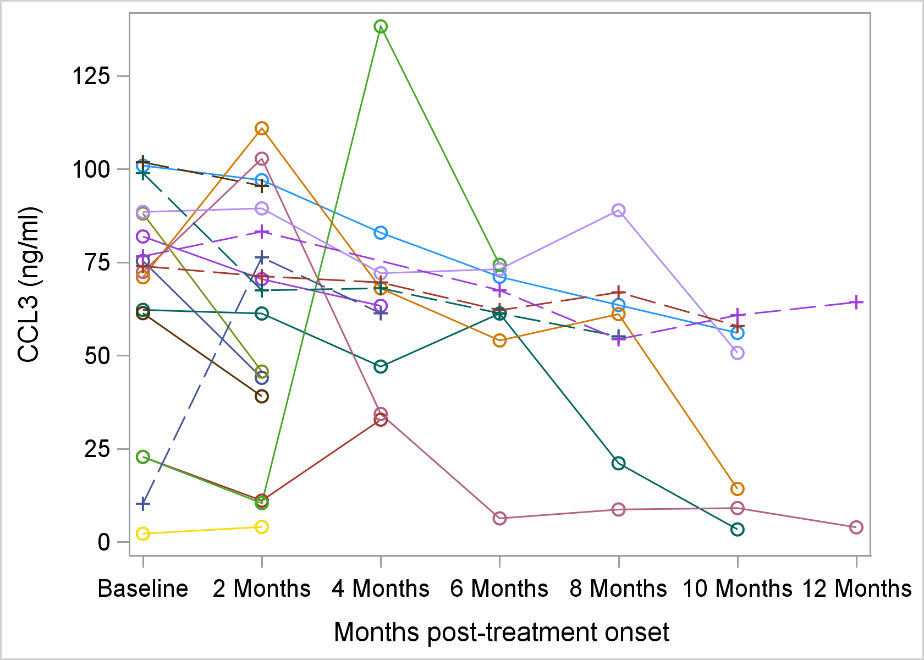

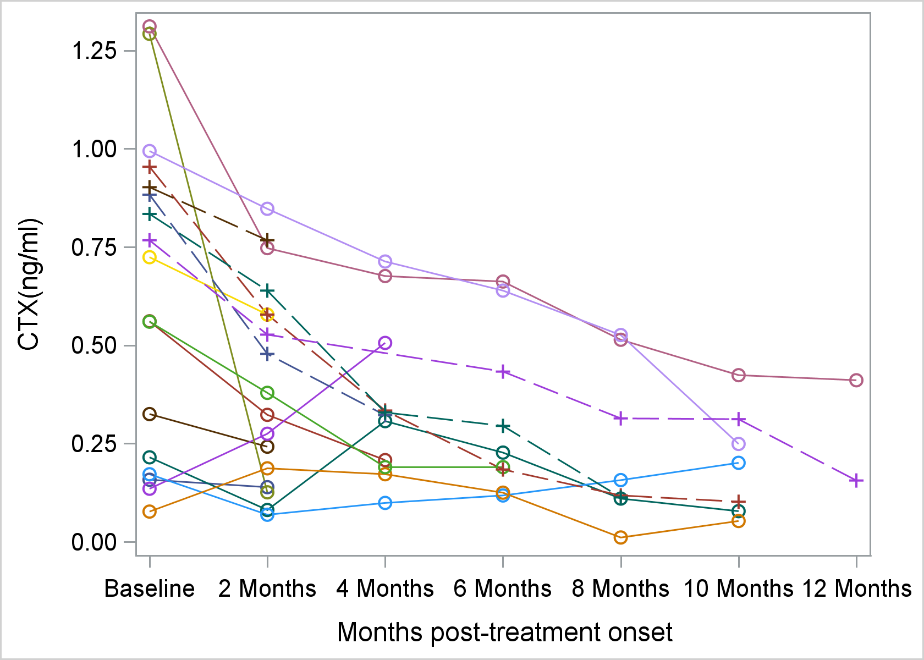

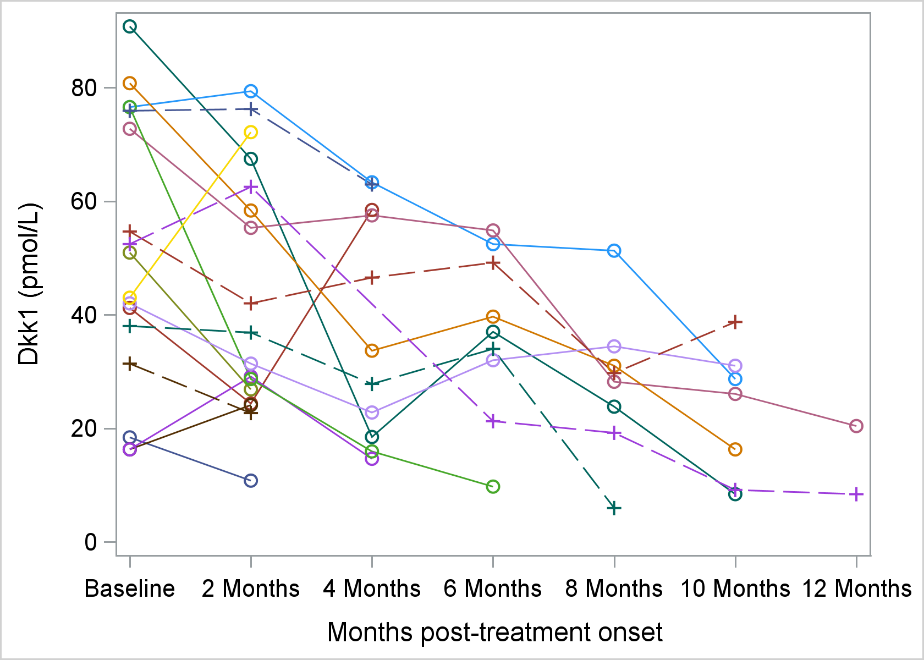

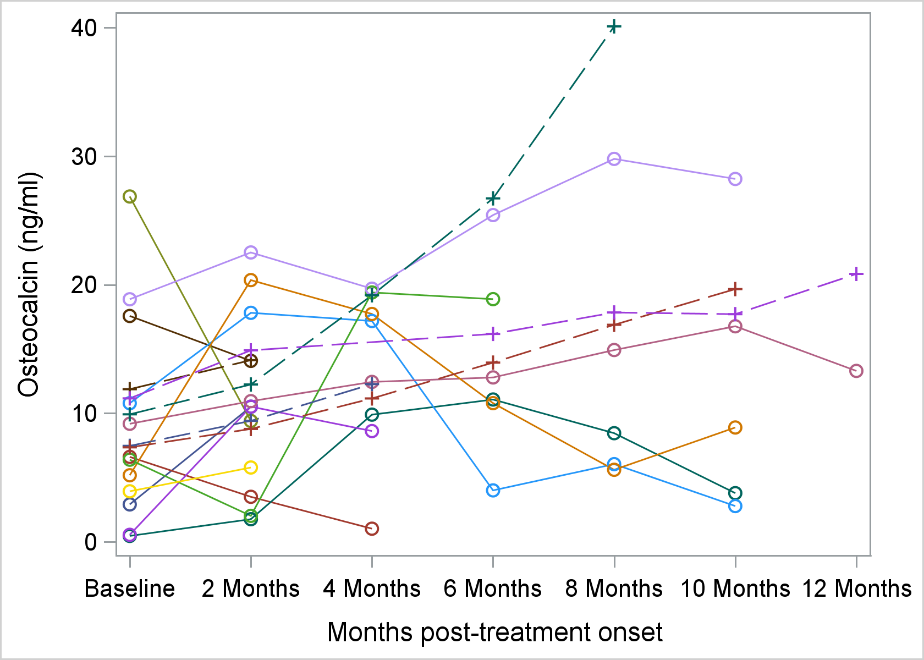

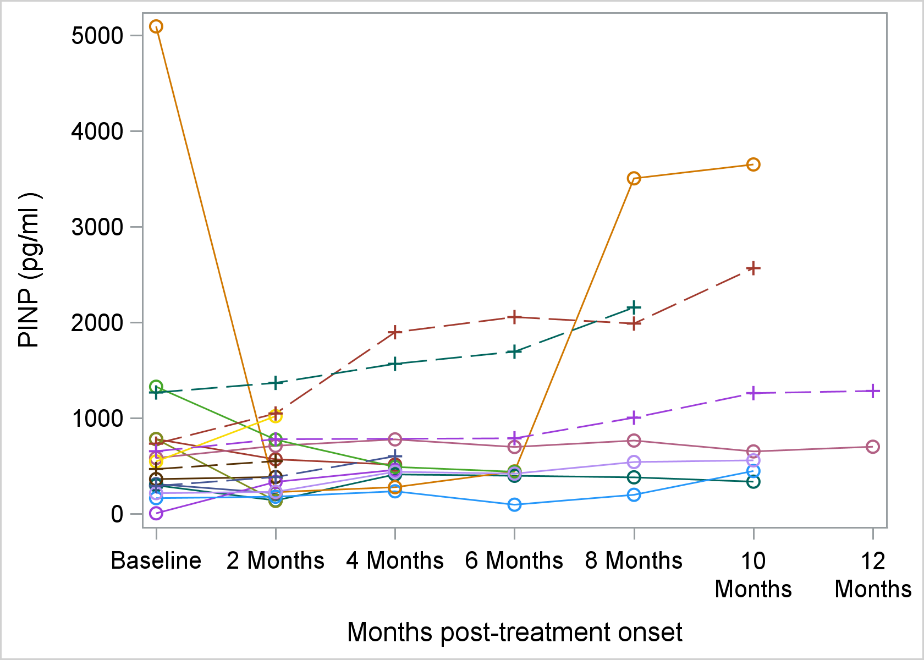

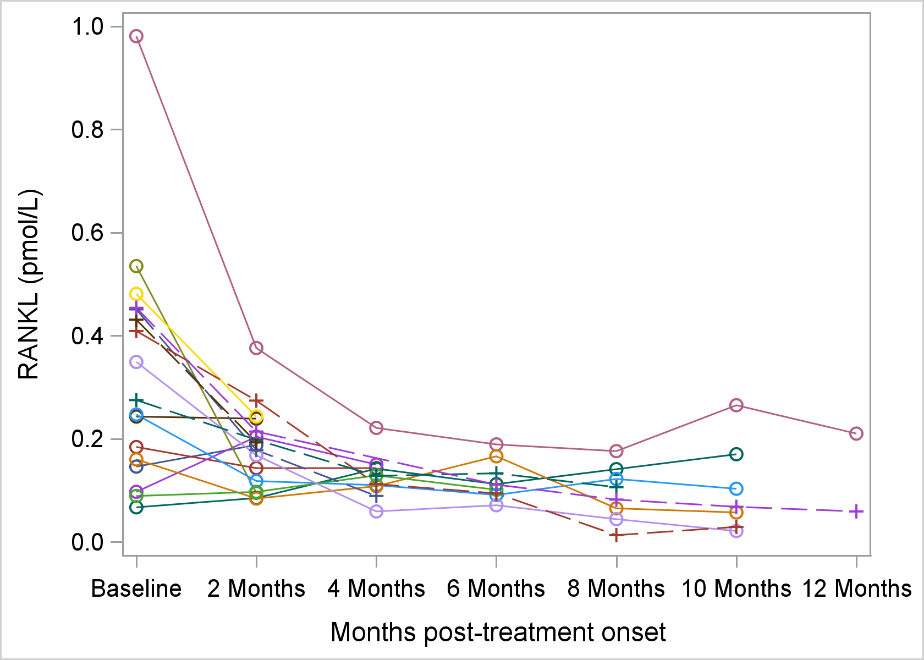

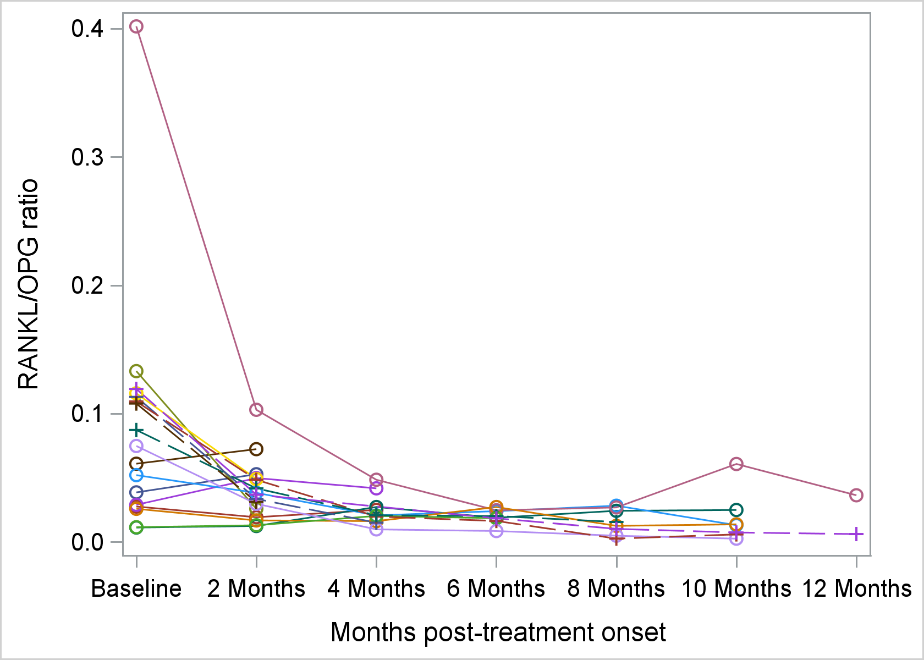

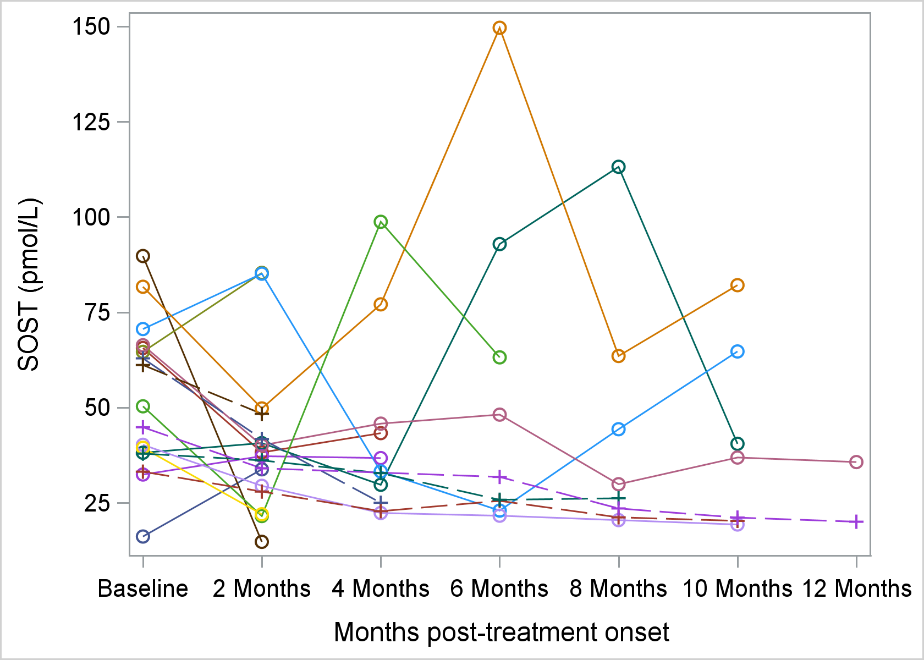


**Figure S2.** Lineplots of biomarker values per patient to depict changes over time, for all patients with at least one post-baseline assessment (*n* = 17).


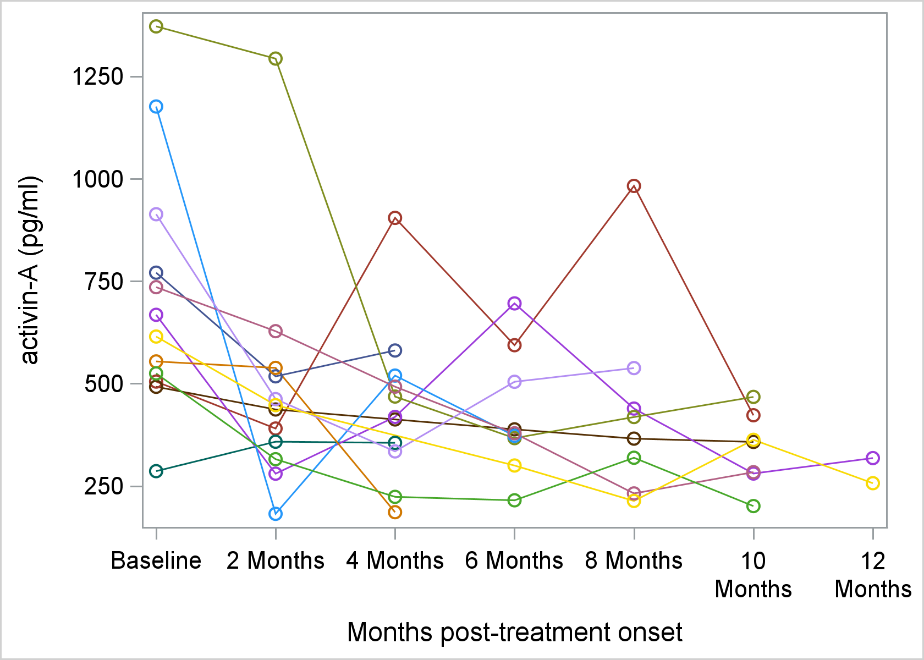

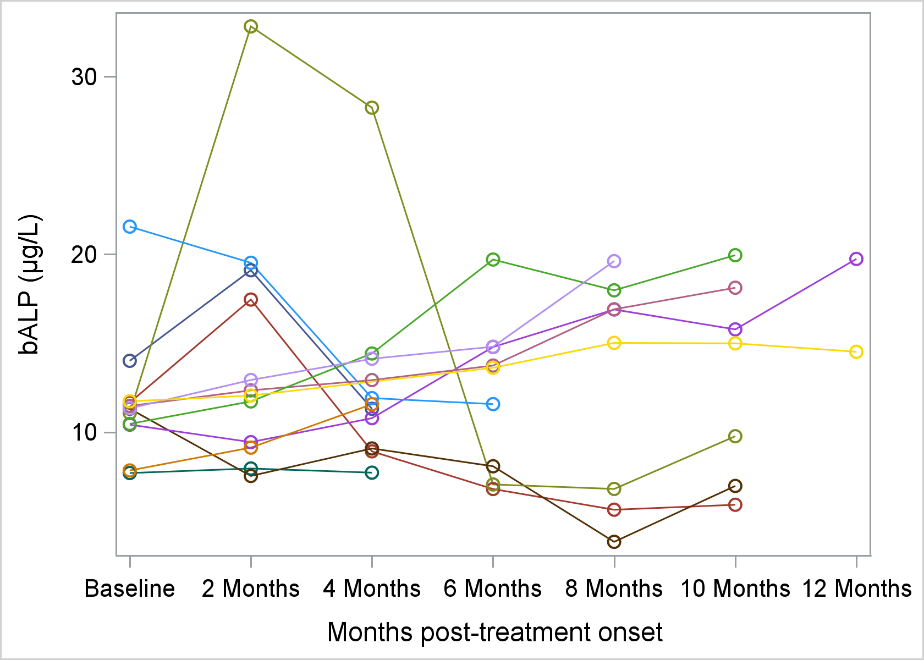

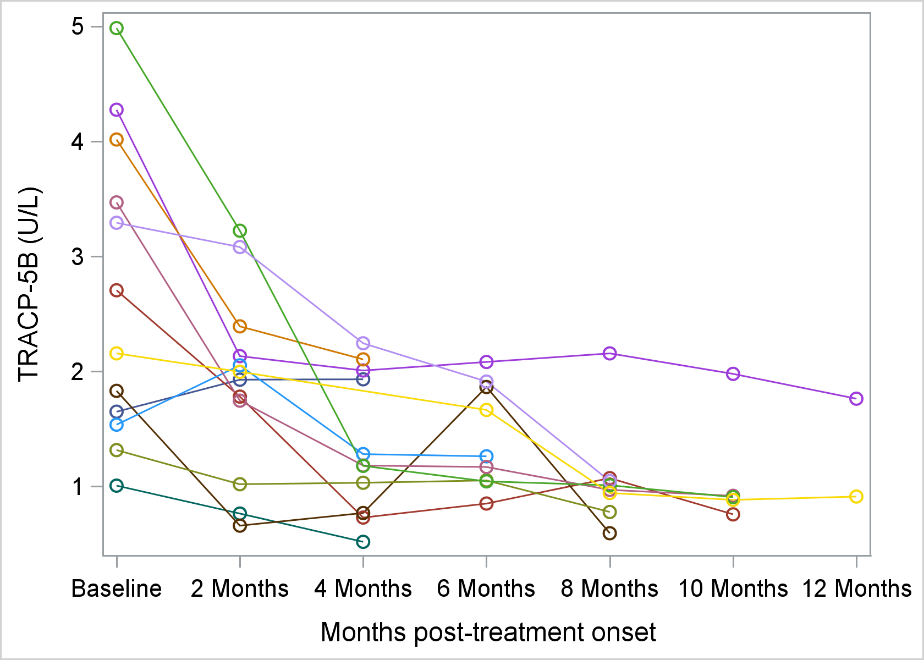

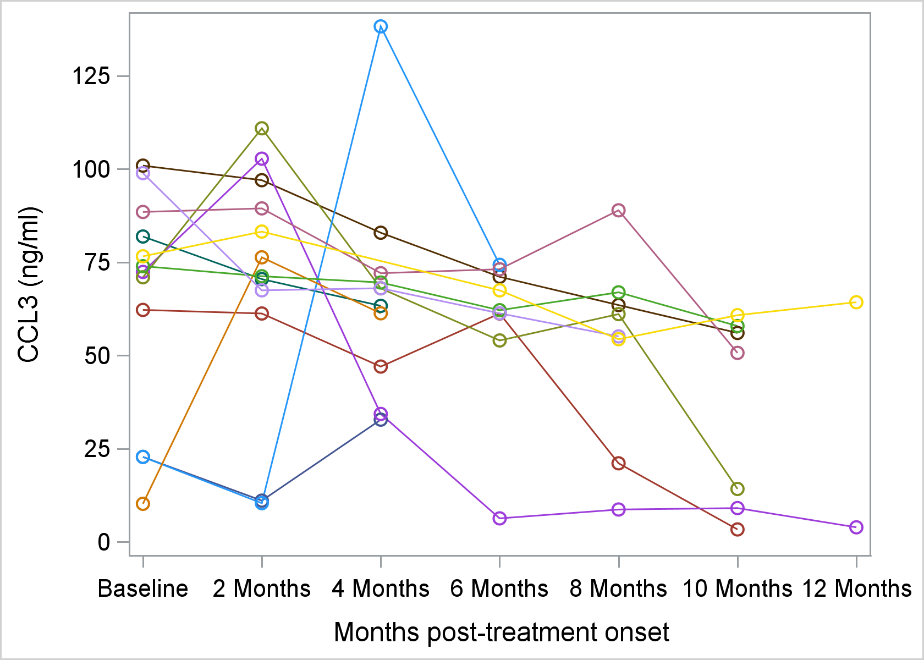

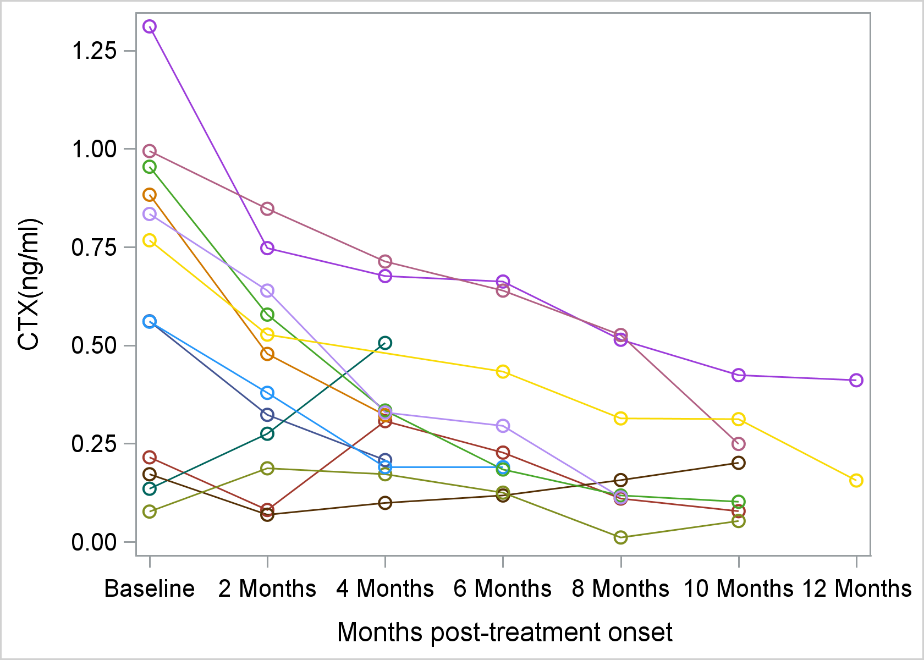

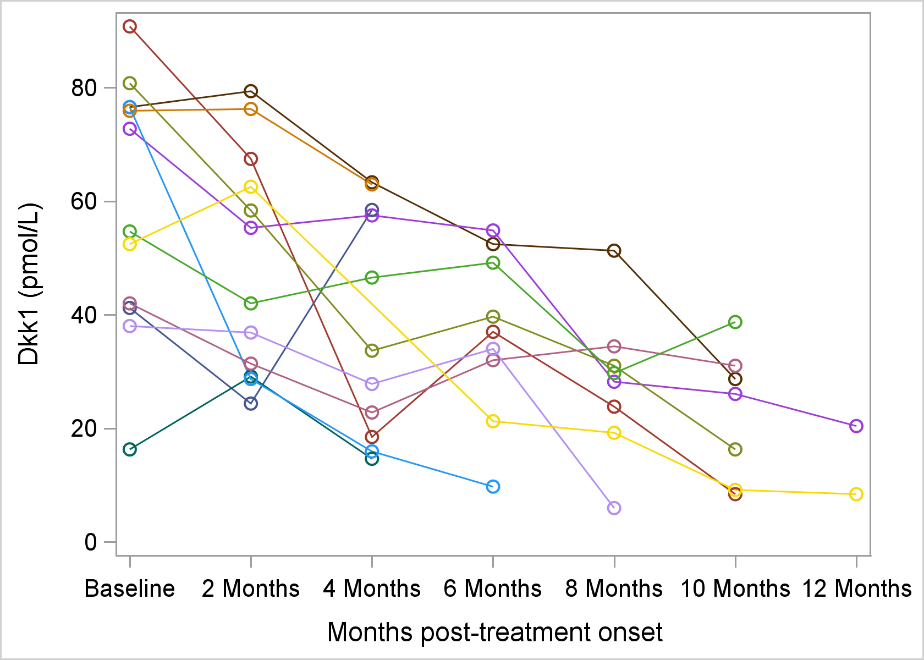

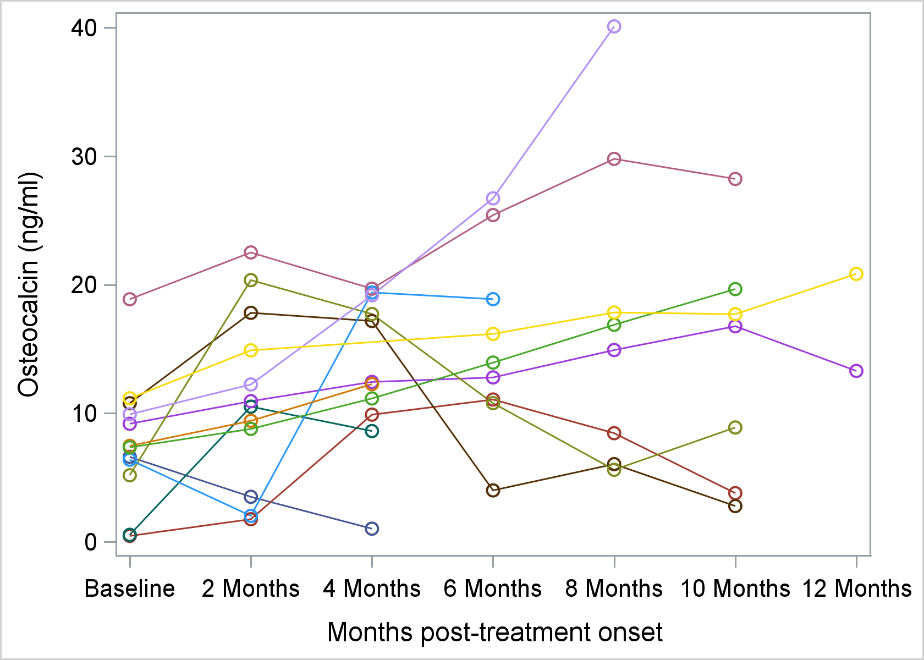

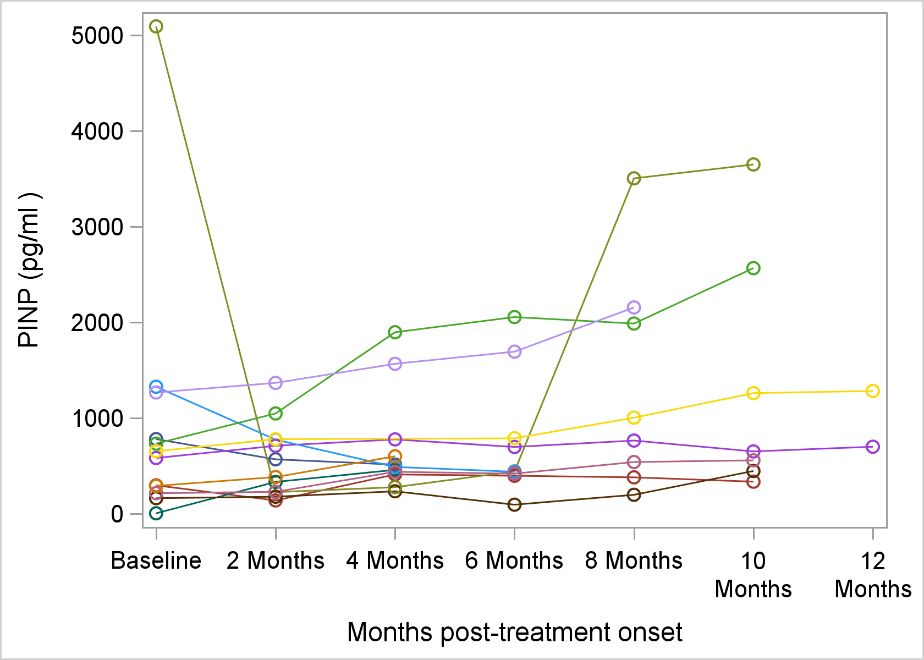

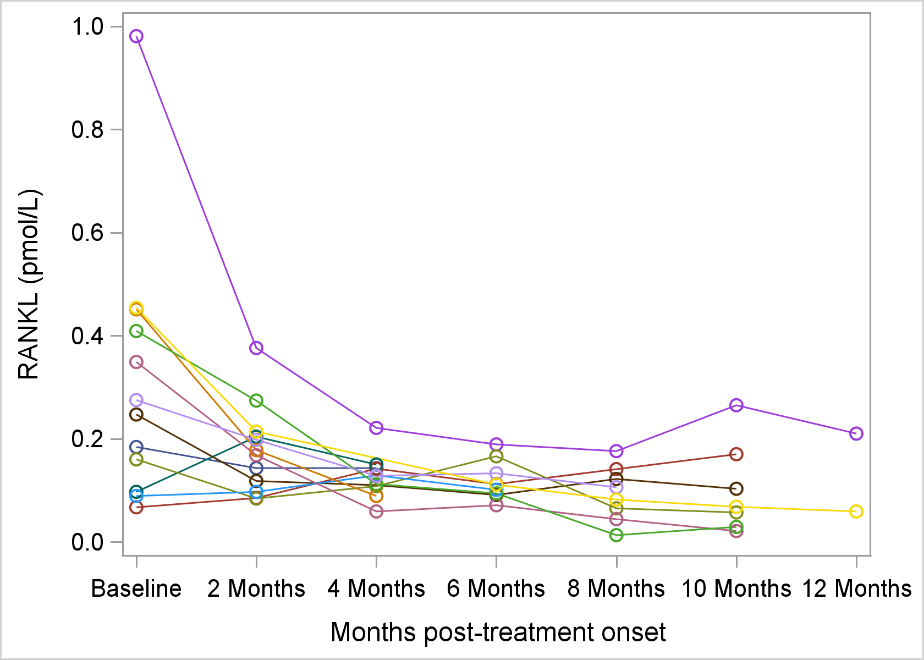

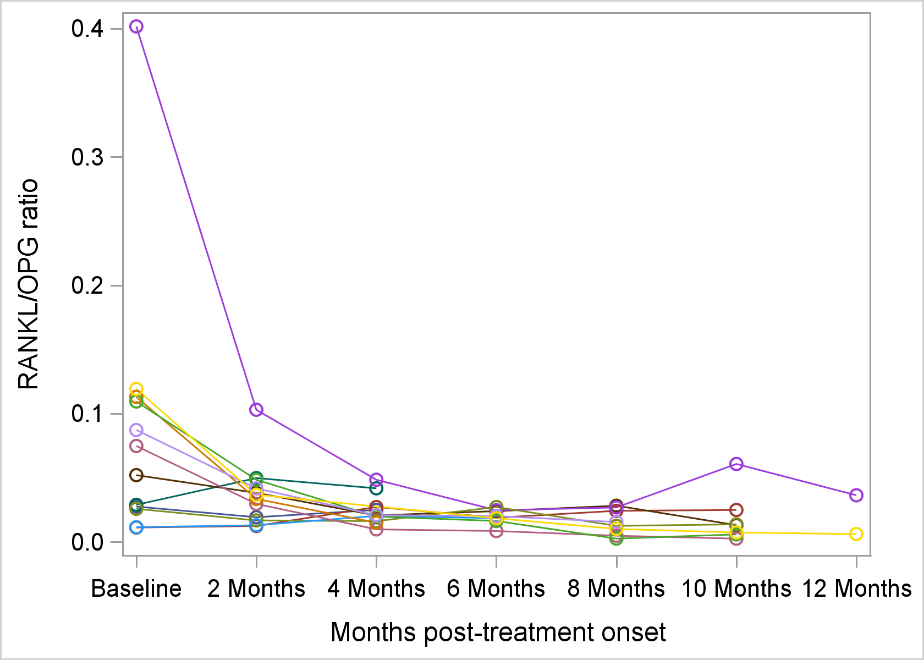

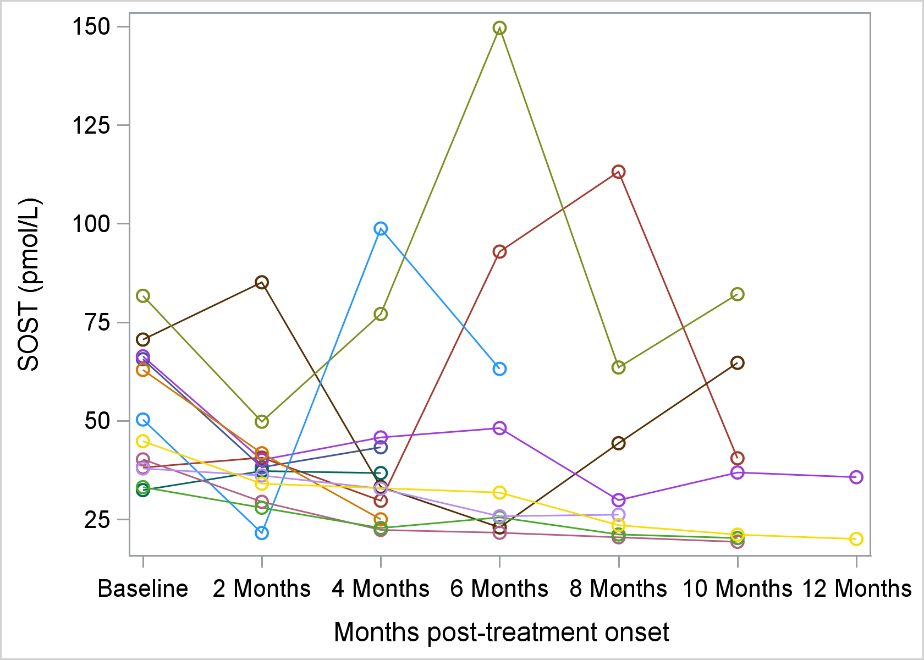


**Figure S3.** Lineplots of biomarker values per patient to depict changes over time, for patients with at least two post-baseline assessment (*n* = 12).
