# Supplementary material for: Carfilzomib Improves Bone Metabolism in Patients with Advanced Relapsed/Refractory Multiple Myeloma: Results of the CarMMa Study
Source: Cancers (Basel). 2021 Mar 12;13(6):1257. doi: 10.3390/cancers13061257 (PMC7998249; doi:10.3390/cancers13061257)
Supplement: Supplementary file 1 [file cancers-13-01257-s001.zip › cancers-1126248-supplementary-1/Supplemental Tables.pdf]

Table S1. Biomarker values at each timepoint, by sex.

		Male	Female	
		n (%)	n (%)	p-value[a]
	bALP (µg/L)			
bALP (µg/L) at baseline	n	11	13	>0.999
	Median	11,1	10,7	
	Q1-Q3	8.9-11.7	9.3-11.5	
bALP (µg/L) at 2 Months	n	9	8	0,810
	Median	11,7	12,6	
	Q1-Q3	8.0-17.5	9.6-14.2	
bALP (µg/L) at 4 Months	n	6	5	0,648
	Median	11	11,9	
	Q1-Q3	8.9-14.4	11.6-12.9	
bALP (µg/L) at 6 Months	n	5	4	0,903
	Median	13,6	12,7	
	Q1-Q3	7.1-14.8	9.8-14.3	
bALP (µg/L) at 8 Months	n	5	3	0,766
	Median	15	16,9	
	Q1-Q3	6.8-16.9	3.8-19.6	
bALP (µg/L) at 10 Months	n	5	2	>0.999
	Median	15	12,5	
	Q1-Q3	9.8-15.8	7.0-18.1	
bALP (µg/L) at 12 Months	n	2	0	
	Median	17,1	.	
	Q1-Q3	14.5-19.7	.-.	
	Osteocalcin (ng/ml)			
Osteocalcin (ng/ml) at baseline	n	11	13	0,082
	Median	6,6	10,1	
	Q1-Q3	2.5-11.2	7.5-11.9	
Osteocalcin (ng/ml) at 2 Months	n	9	8	0,810
	Median	10,5	10,8	
	Q1-Q3	8.8-14.1	7.6-16.0	
Osteocalcin (ng/ml) at 4 Months	n	6	5	**0,036**
	Median	10,5	19,2	
	Q1-Q3	8.6-12.4	17.2-19.4	
Osteocalcin (ng/ml) at 6 Months	n	5	4	0,270
	Median	12,8	22,2	
	Q1-Q3	11.1-13.9	11.4-26.1	
Osteocalcin (ng/ml) at 8 Months	n	5	3	0,371
	Median	14,9	29,8	
	Q1-Q3	8.5-16.9	6.0-40.1	
Osteocalcin (ng/ml) at 10 Months	n	5	2	>0.999
	Median	16,8	15,5	
	Q1-Q3	8.9-17.7	2.8-28.2	
Osteocalcin (ng/ml) at 12 Months	n	2	0	
	Median	17,1	.	
	Q1-Q3	13.3-20.8	.-.	
	PINP (pg/ml)			
PINP (pg/ml) at baseline	n	11	13	0,602
	Median	583,2	537	

	Q1-Q3	310.5-750.7	253.1-742.1	
PINP (pg/ml) at 2 Months	n	9	8	0,962
	Median	384,9	467,5	
	Q1-Q3	226.3-710.7	204.2-897.9	
PINP (pg/ml) at 4 Months	n	6	5	0,927
	Median	486,8	490,2	
	Q1-Q3	411.6-777.5	439.2-601.4	
PINP (pg/ml) at 6 Months	n	5	4	0,391
	Median	698,7	429,5	
	Q1-Q3	442.8-789.0	257.6-1067	
PINP (pg/ml) at 8 Months	n	5	3	0,551
	Median	1004	541,1	
	Q1-Q3	765.5-1988	198.9-2156	
PINP (pg/ml) at 10 Months	n	5	2	0,333
	Median	1261	503,3	
	Q1-Q3	652.0-2567	447.6-559.0	
PINP (pg/ml) at 12 Months	n	2	0	
	Median	992,5	.	
	Q1-Q3	701.3-1284	.-.	
	**CTX(ng/ml)**			
CTX(ng/ml) at baseline	n	11	13	0,164
	Median	0,3	0,8	
	Q1-Q3	0.2-1.0	0.7-0.9	
CTX(ng/ml) at 2 Months	n	9	8	0,336
	Median	0,3	0,5	
	Q1-Q3	0.2-0.5	0.3-0.7	
CTX(ng/ml) at 4 Months	n	6	5	0,784
	Median	0,3	0,3	
	Q1-Q3	0.2-0.5	0.2-0.3	
CTX(ng/ml) at 6 Months	n	5	4	0,903
	Median	0,2	0,2	
	Q1-Q3	0.2-0.4	0.2-0.5	
CTX(ng/ml) at 8 Months	n	5	3	0,551
	Median	0,1	0,2	
	Q1-Q3	0.1-0.3	0.1-0.5	
CTX(ng/ml) at 10 Months	n	5	2	0,846
	Median	0,1	0,2	
	Q1-Q3	0.1-0.3	0.2-0.2	
CTX(ng/ml) at 12 Months	n	2	0	
	Median	0,3	.	
	Q1-Q3	0.2-0.4	.-.	
	**TRACP-5B (U/L)**			
TRACP-5B (U/L) at baseline	n	11	13	0,247
	Median	1,9	3,6	
	Q1-Q3	1.3-4.3	3.3-3.9	
TRACP-5B (U/L) at 2 Months	n	9	8	0,736
	Median	1,8	1,9	
	Q1-Q3	1.0-2.0	1.2-2.2	
TRACP-5B (U/L) at 4 Months	n	6	5	0,235
	Median	1,1	1,3	

	Q1-Q3	0.7-1.9	1.2-2.1	
TRACP-5B (U/L) at 6 Months	n	5	4	0,391
	Median	1,1	1,6	
	Q1-Q3	1.0-1.7	1.2-1.9	
TRACP-5B (U/L) at 8 Months	n	5	3	0,551
	Median	1	1	
	Q1-Q3	0.9-1.1	0.6-1.0	
TRACP-5B (U/L) at 10 Months	n	4	1	0,724
	Median	0,9	0,9	
	Q1-Q3	0.8-1.4	0.9-0.9	
TRACP-5B (U/L) at 12 Months	n	2	0	
	Median	1,3	.	
	Q1-Q3	0.9-1.8	.-.	
	**RANKL (pmol/L)**			
RANKL (pmol/L) at baseline	n	11	13	0,224
	Median	0,2	0,3	
	Q1-Q3	0.1-0.4	0.2-0.4	
RANKL (pmol/L) at 2 Months	n	9	8	0,413
	Median	0,2	0,2	
	Q1-Q3	0.1-0.2	0.1-0.2	
RANKL (pmol/L) at 4 Months	n	6	5	0,083
	Median	0,1	0,1	
	Q1-Q3	0.1-0.2	0.1-0.1	
RANKL (pmol/L) at 6 Months	n	5	4	0,178
	Median	0,1	0,1	
	Q1-Q3	0.1-0.2	0.1-0.1	
RANKL (pmol/L) at 8 Months	n	5	3	>0.999
	Median	0,1	0,1	
	Q1-Q3	0.1-0.1	0.0-0.1	
RANKL (pmol/L) at 10 Months	n	5	2	0,561
	Median	0,1	0,1	
	Q1-Q3	0.1-0.2	0.0-0.1	
RANKL (pmol/L) at 12 Months	n	2	0	
	Median	0,1	.	
	Q1-Q3	0.1-0.2	.-.	
	**RANKL/OPG ratio**			
RANKL/OPG ratio at baseline	n	11	13	0,297
	Median	0	0,1	
	Q1-Q3	0.0-0.1	0.1-0.1	
RANKL/OPG ratio at 2 Months	n	9	8	0,361
	Median	0	0	
	Q1-Q3	0.0-0.1	0.0-0.0	
RANKL/OPG ratio at 4 Months	n	6	5	0,121
	Median	0	0	
	Q1-Q3	0.0-0.0	0.0-0.0	
RANKL/OPG ratio at 6 Months	n	5	4	0,713
	Median	0	0	
	Q1-Q3	0.0-0.0	0.0-0.0	
RANKL/OPG ratio at 8 Months	n	5	3	0,766
	Median	0	0	

	Q1-Q3	0.0-0.0	0.0-0.0	
RANKL/OPG ratio at 10 Months	n	5	2	0,333
	Median	0	0	
	Q1-Q3	0.0-0.0	0.0-0.0	
RANKL/OPG ratio at 12 Months	n	2	0	
	Median	0	.	
	Q1-Q3	0.0-0.0	.-.	
	**SOST (pmol/L)**			
SOST (pmol/L) at baseline	n	11	13	0,817
	Median	44,8	50,3	
	Q1-Q3	33.2-66.3	40.2-64.1	
SOST (pmol/L) at 2 Months	n	9	8	0,597
	Median	37,2	38,9	
	Q1-Q3	33.8-40.1	25.7-66.7	
SOST (pmol/L) at 4 Months	n	6	5	0,523
	Median	40	32,8	
	Q1-Q3	29.7-45.8	25.0-33.2	
SOST (pmol/L) at 6 Months	n	5	4	0,178
	Median	48,1	24,4	
	Q1-Q3	31.8-92.9	22.3-44.5	
SOST (pmol/L) at 8 Months	n	5	3	0,551
	Median	29,9	26,2	
	Q1-Q3	23.5-63.5	20.4-44.3	
SOST (pmol/L) at 10 Months	n	5	2	0,846
	Median	36,9	42	
	Q1-Q3	21.1-40.5	19.3-64.7	
SOST (pmol/L) at 12 Months	n	2	0	
	Median	27,8	.	
	Q1-Q3	20.0-35.7	.-.	
	**Dkk1 (pmol/L)**			
Dkk1 (pmol/L) at baseline	n	11	13	0,685
	Median	46,8	38	
	Q1-Q3	18.4-72.8	31.4-50.9	
Dkk1 (pmol/L) at 2 Months	n	9	8	0,597
	Median	42	34,1	
	Q1-Q3	24.4-58.4	27.8-74.2	
Dkk1 (pmol/L) at 4 Months	n	6	5	0,784
	Median	40,1	27,8	
	Q1-Q3	18.5-57.5	22.8-62.9	
Dkk1 (pmol/L) at 6 Months	n	5	4	0,391
	Median	39,7	33	
	Q1-Q3	37.0-49.2	20.9-43.2	
Dkk1 (pmol/L) at 8 Months	n	5	3	0,551
	Median	28,2	34,4	
	Q1-Q3	23.8-29.7	5.9-51.3	
Dkk1 (pmol/L) at 10 Months	n	5	2	0,333
	Median	16,3	29,8	
	Q1-Q3	9.1-26.1	28.7-31.0	
Dkk1 (pmol/L) at 12 Months	n	2	0	
	Median	14,4	.	

	Q1-Q3	8.4-20.4	.-.	
	**activin-A (pg/ml)**			
activin-A (pg/ml) at baseline	n	11	13	0,487
	Median	614,9	735,4	
	Q1-Q3	505.0-770.8	492.3-913.5	
activin-A (pg/ml) at 2 Months	n	9	8	0,229
	Median	390,4	507,8	
	Q1-Q3	321.0-488.4	449.6-595.1	
activin-A (pg/ml) at 4 Months	n	6	5	0,523
	Median	443,5	412,5	
	Q1-Q3	355.4-580.9	334.5-492.5	
activin-A (pg/ml) at 6 Months	n	5	4	0,713
	Median	366,9	383,5	
	Q1-Q3	300.0-593.8	375.7-446.4	
activin-A (pg/ml) at 8 Months	n	5	3	>0.999
	Median	418,4	365,7	
	Q1-Q3	319.0-439.0	231.9-537.9	
activin-A (pg/ml) at 10 Months	n	5	2	0,846
	Median	362,8	320,7	
	Q1-Q3	280.5-422.7	283.8-357.5	
activin-A (pg/ml) at 12 Months	n	2	0	
	Median	287,5	.	
	Q1-Q3	256.8-318.2	.-.	
	**CCL3 (ng/ml)**			
CCL3 (ng/ml) at baseline	n	11	13	0,385
	Median	73,9	88,1	
	Q1-Q3	62.2-81.3	22.8-98.9	
CCL3 (ng/ml) at 2 Months	n	9	8	0,885
	Median	70,5	71,9	
	Q1-Q3	44.0-83.2	28.0-92.4	
CCL3 (ng/ml) at 4 Months	n	6	5	0,055
	Median	55,1	72	
	Q1-Q3	34.3-68.0	68.0-82.9	
CCL3 (ng/ml) at 6 Months	n	5	4	0,066
	Median	61,2	72,1	
	Q1-Q3	54.0-62.1	66.2-73.7	
CCL3 (ng/ml) at 8 Months	n	5	3	0,233
	Median	54,3	63,5	
	Q1-Q3	21.1-61.1	55.1-88.9	
CCL3 (ng/ml) at 10 Months	n	5	2	0,846
	Median	14,2	53,3	
	Q1-Q3	9.1-57.9	50.7-56.0	
CCL3 (ng/ml) at 12 Months	n	2	0	
	Median	34,1	.	
	Q1-Q3	3.9-64.3	.-.	

[a] Mann-Whitney U test

Table S2. Biomarker values at each timepoint, by ECOG PS at Kd initiation.

		ECOG		
		0-1	2 or higher	
		n (%)	n (%)	p-value[a]
	**bALP (μg/L)**			
bALP (μg/L) at baseline	n	20	4	0,810
	Median	10,6	14,8	
	Q1-Q3	8.6-11.4	10.9-25.5	
bALP (μg/L) at 2 Months	n	16	1	0,475
	Median	11,9	15,4	
	Q1-Q3	8.5-15.2	15.4-15.4	
bALP (μg/L) at 4 Months	n	11	0	
	Median	11,6	.	
	Q1-Q3	9.1-14.1	.-.	
bALP (μg/L) at 6 Months	n	9	0	
	Median	13,6	.	
	Q1-Q3	8.1-14.8	.-.	
bALP (μg/L) at 8 Months	n	8	0	
	Median	16	.	
	Q1-Q3	6.2-17.4	.-.	
bALP (μg/L) at 10 Months	n	7	0	
	Median	15	.	
	Q1-Q3	7.0-18.1	.-.	
bALP (μg/L) at 12 Months	n	2	0	
	Median	17,1	.	
	Q1-Q3	14.5-19.7	.-.	
	**Osteocalcin (ng/ml)**			
Osteocalcin (ng/ml) at baseline	n	20	4	**0,027**
	Median	7,4	11,6	
	Q1-Q3	4.6-10.8	10.7-19.4	
Osteocalcin (ng/ml) at 2 Months	n	16	1	0,610
	Median	10,7	9,4	
	Q1-Q3	7.3-14.5	9.4-9.4	
Osteocalcin (ng/ml) at 4 Months	n	11	0	
	Median	12,4	.	
	Q1-Q3	9.9-19.2	.-.	
Osteocalcin (ng/ml) at 6 Months	n	9	0	
	Median	13,9	.	
	Q1-Q3	11.1-18.9	.-.	
Osteocalcin (ng/ml) at 8 Months	n	8	0	
	Median	15,9	.	
	Q1-Q3	7.3-23.8	.-.	
Osteocalcin (ng/ml) at 10 Months	n	7	0	
	Median	16,8	.	
	Q1-Q3	3.8-19.7	.-.	
Osteocalcin (ng/ml) at 12 Months	n	2	0	
	Median	17,1	.	
	Q1-Q3	13.3-20.8	.-.	
	**PINP (pg/ml)**			
PINP (pg/ml) at baseline	n	20	4	0,131

	Median	477,8	746,4	
	Q1-Q3	272.6-693.6	639.5-766.1	
PINP (pg/ml) at 2 Months	n	16	1	0,126
	Median	467,8	135,1	
	Q1-Q3	227.9-777.4	135.1-135.1	
PINP (pg/ml) at 4 Months	n	11	0	
	Median	490,2	.	
	Q1-Q3	411.6-777.5	.-.	
PINP (pg/ml) at 6 Months	n	9	0	
	Median	442,8	.	
	Q1-Q3	419.7-789.0	.-.	
PINP (pg/ml) at 8 Months	n	8	0	
	Median	884,9	.	
	Q1-Q3	461.1-2072	.-.	
PINP (pg/ml) at 10 Months	n	7	0	
	Median	652	.	
	Q1-Q3	447.6-2567	.-.	
PINP (pg/ml) at 12 Months	n	2	0	
	Median	992,5	.	
	Q1-Q3	701.3-1284	.-.	
	**CTX(ng/ml)**			
CTX(ng/ml) at baseline	n	20	4	**0,018**
	Median	0,6	1,2	
	Q1-Q3	0.2-0.9	1.0-1.6	
CTX(ng/ml) at 2 Months	n	16	1	0,261
	Median	0,4	0,1	
	Q1-Q3	0.2-0.6	0.1-0.1	
CTX(ng/ml) at 4 Months	n	11	0	
	Median	0,3	.	
	Q1-Q3	0.2-0.5	.-.	
CTX(ng/ml) at 6 Months	n	9	0	
	Median	0,2	.	
	Q1-Q3	0.2-0.4	.-.	
CTX(ng/ml) at 8 Months	n	8	0	
	Median	0,1	.	
	Q1-Q3	0.1-0.4	.-.	
CTX(ng/ml) at 10 Months	n	7	0	
	Median	0,2	.	
	Q1-Q3	0.1-0.3	.-.	
CTX(ng/ml) at 12 Months	n	2	0	
	Median	0,3	.	
	Q1-Q3	0.2-0.4	.-.	
	**TRACP-5B (U/L)**			
TRACP-5B (U/L) at baseline	n	20	4	**0,009**
	Median	2,4	5,6	
	Q1-Q3	1.6-3.7	4.3-6.7	
TRACP-5B (U/L) at 2 Months	n	16	1	0,185
	Median	1,9	0,6	
	Q1-Q3	1.4-2.1	0.6-0.6	
TRACP-5B (U/L) at 4 Months	n	11	0	

	Median	1,2	.	
	Q1-Q3	0.8-2.0	.-.	
TRACP-5B (U/L) at 6 Months	n	9	0	
	Median	1,3	.	
	Q1-Q3	1.1-1.9	.-.	
TRACP-5B (U/L) at 8 Months	n	8	0	
	Median	1	.	
	Q1-Q3	0.9-1.1	.-.	
TRACP-5B (U/L) at 10 Months	n	5	0	
	Median	0,9	.	
	Q1-Q3	0.9-0.9	.-.	
TRACP-5B (U/L) at 12 Months	n	2	0	
	Median	1,3	.	
	Q1-Q3	0.9-1.8	.-.	
	**RANKL (pmol/L)**			
RANKL (pmol/L) at baseline	n	20	4	0,296
	Median	0,2	0,4	
	Q1-Q3	0.1-0.4	0.3-0.5	
RANKL (pmol/L) at 2 Months	n	16	1	0,262
	Median	0,2	0,1	
	Q1-Q3	0.1-0.2	0.1-0.1	
RANKL (pmol/L) at 4 Months	n	11	0	
	Median	0,1	.	
	Q1-Q3	0.1-0.1	.-.	
RANKL (pmol/L) at 6 Months	n	9	0	
	Median	0,1	.	
	Q1-Q3	0.1-0.1	.-.	
RANKL (pmol/L) at 8 Months	n	8	0	
	Median	0,1	.	
	Q1-Q3	0.1-0.1	.-.	
RANKL (pmol/L) at 10 Months	n	7	0	
	Median	0,1	.	
	Q1-Q3	0.0-0.2	.-.	
RANKL (pmol/L) at 12 Months	n	2	0	
	Median	0,1	.	
	Q1-Q3	0.1-0.2	.-.	
	**RANKL/OPG ratio**			
RANKL/OPG ratio at baseline	n	20	4	0,261
	Median	0,1	0,1	
	Q1-Q3	0.0-0.1	0.1-0.1	
RANKL/OPG ratio at 2 Months	n	16	1	0,475
	Median	0	0	
	Q1-Q3	0.0-0.0	0.0-0.0	
RANKL/OPG ratio at 4 Months	n	11	0	
	Median	0	.	
	Q1-Q3	0.0-0.0	.-.	
RANKL/OPG ratio at 6 Months	n	9	0	
	Median	0	.	
	Q1-Q3	0.0-0.0	.-.	
RANKL/OPG ratio at 8 Months	n	8	0	

	Median	0	.	
	Q1-Q3	0.0-0.0	.-.	
RANKL/OPG ratio at 10 Months	n	7	0	
	Median	0	.	
	Q1-Q3	0.0-0.0	.-.	
RANKL/OPG ratio at 12 Months	n	2	0	
	Median	0	.	
	Q1-Q3	0.0-0.0	.-.	
	**SOST (pmol/L)**			
SOST (pmol/L) at baseline	n	20	4	0,462
	Median	43,1	60,2	
	Q1-Q3	37.8-66.0	49.0-64.4	
SOST (pmol/L) at 2 Months	n	16	1	0,126
	Median	36,7	85,3	
	Q1-Q3	28.7-41.2	85.3-85.3	
SOST (pmol/L) at 4 Months	n	11	0	
	Median	33,2	.	
	Q1-Q3	25.0-45.8	.-.	
SOST (pmol/L) at 6 Months	n	9	0	
	Median	31,8	.	
	Q1-Q3	25.5-63.1	.-.	
SOST (pmol/L) at 8 Months	n	8	0	
	Median	28	.	
	Q1-Q3	22.3-53.9	.-.	
SOST (pmol/L) at 10 Months	n	7	0	
	Median	36,9	.	
	Q1-Q3	20.2-64.7	.-.	
SOST (pmol/L) at 12 Months	n	2	0	
	Median	27,8	.	
	Q1-Q3	20.0-35.7	.-.	
	**Dkk1 (pmol/L)**			
Dkk1 (pmol/L) at baseline	n	20	4	0,295
	Median	42,5	30,3	
	Q1-Q3	31.7-74.3	23.1-43.2	
Dkk1 (pmol/L) at 2 Months	n	16	1	0,475
	Median	39,4	26,9	
	Q1-Q3	26.5-65.0	26.9-26.9	
Dkk1 (pmol/L) at 4 Months	n	11	0	
	Median	33,7	.	
	Q1-Q3	18.5-58.4	.-.	
Dkk1 (pmol/L) at 6 Months	n	9	0	
	Median	37	.	
	Q1-Q3	32.0-49.2	.-.	
Dkk1 (pmol/L) at 8 Months	n	8	0	
	Median	29	.	
	Q1-Q3	21.5-32.7	.-.	
Dkk1 (pmol/L) at 10 Months	n	7	0	
	Median	26,1	.	
	Q1-Q3	9.1-31.0	.-.	
Dkk1 (pmol/L) at 12 Months	n	2	0	

	Median	14,4	.	
	Q1-Q3	8.4-20.4	.-.	
	**activin-A (pg/ml)**			
activin-A (pg/ml) at baseline	n	20	4	0,201
	Median	681,1	484,7	
	Q1-Q3	514.9-908.1	330.1-726.3	
activin-A (pg/ml) at 2 Months	n	16	1	0,358
	Median	454,8	562,2	
	Q1-Q3	339.6-528.0	562.2-562.2	
activin-A (pg/ml) at 4 Months	n	11	0	
	Median	418,7	.	
	Q1-Q3	334.5-519.6	.-.	
activin-A (pg/ml) at 6 Months	n	9	0	
	Median	378,7	.	
	Q1-Q3	366.9-504.5	.-.	
activin-A (pg/ml) at 8 Months	n	8	0	
	Median	392	.	
	Q1-Q3	275.4-488.5	.-.	
activin-A (pg/ml) at 10 Months	n	7	0	
	Median	357,5	.	
	Q1-Q3	280.5-422.7	.-.	
activin-A (pg/ml) at 12 Months	n	2	0	
	Median	287,5	.	
	Q1-Q3	256.8-318.2	.-.	
	**CCL3 (ng/ml)**			
CCL3 (ng/ml) at baseline	n	20	4	**0,040**
	Median	74,6	96,9	
	Q1-Q3	42.1-85.2	83.5-107.3	
CCL3 (ng/ml) at 2 Months	n	16	1	0,610
	Median	70,8	45,7	
	Q1-Q3	41.5-92.4	45.7-45.7	
CCL3 (ng/ml) at 4 Months	n	11	0	
	Median	68	.	
	Q1-Q3	47.0-72.0	.-.	
CCL3 (ng/ml) at 6 Months	n	9	0	
	Median	62,1	.	
	Q1-Q3	61.2-71.1	.-.	
CCL3 (ng/ml) at 8 Months	n	8	0	
	Median	58,1	.	
	Q1-Q3	37.7-65.2	.-.	
CCL3 (ng/ml) at 10 Months	n	7	0	
	Median	50,7	.	
	Q1-Q3	9.1-57.9	.-.	
CCL3 (ng/ml) at 12 Months	n	2	0	
	Median	34,1	.	
	Q1-Q3	3.9-64.3	.-.	

[a] Mann-Whitney U test; [b] Kruskal-Wallis test

Table S3. Biomarker values at each timepoint, by ISS stage at diagnosis

		ISS at diagnosis			
		I	II	III	
		n (%)	n (%)	n (%)	p-value[a]
	**bALP (µg/L)**				
bALP (µg/L) at baseline	n	8	8	8	0,990
	Median	11	10,8	10,8	
	Q1-Q3	8.6-12.9	9.7-11.5	9.3-11.6	
bALP (µg/L) at 2 Months	n	6	4	7	0,230
	Median	9	11,2	12,8	
	Q1-Q3	7.6-12.1	8.6-16.2	11.7-17.5	
bALP (µg/L) at 4 Months	n	3	3	5	0,177
	Median	9,1	11,9	12,9	
	Q1-Q3	7.7-11.3	10.8-14.1	11.6-14.4	
bALP (µg/L) at 6 Months	n	2	3	4	0,574
	Median	10,9	14,8	10,4	
	Q1-Q3	8.1-13.6	11.6-14.8	6.9-16.7	
bALP (µg/L) at 8 Months	n	2	2	4	0,264
	Median	9,4	18,3	11,9	
	Q1-Q3	3.8-15.0	16.9-19.6	6.2-17.4	
bALP (µg/L) at 10 Months	n	2	1	4	0,706
	Median	11	15,8	13,9	
	Q1-Q3	7.0-15.0	15.8-15.8	7.8-19.0	
bALP (µg/L) at 12 Months	n	1	1	0	
	Median	14,5	19,7	.	
	Q1-Q3	14.5-14.5	19.7-19.7	.-.	
	**Osteocalcin (ng/ml)**				
Osteocalcin (ng/ml) at baseline	n	8	8	8	0,724
	Median	11	9,6	7,4	
	Q1-Q3	6.2-11.6	4.7-10.5	4.6-14.0	
Osteocalcin (ng/ml) at 2 Months	n	6	4	7	0,482
	Median	14,1	10,7	9,4	
	Q1-Q3	10.5-14.9	6.3-11.6	5.8-20.4	

Osteocalcin (ng/ml) at 4 Months	n	3	3	5	0,179
	Median	8,6	19,2	12,3	
	Q1-Q3	1.0-17.2	12.4-19.4	11.2-17.7	
Osteocalcin (ng/ml) at 6 Months	n	2	3	4	0,398
	Median	10,1	18,9	12,5	
	Q1-Q3	4.0-16.2	12.8-26.7	10.9-19.7	
Osteocalcin (ng/ml) at 8 Months	n	2	2	4	0,607
	Median	11,9	27,5	12,7	
	Q1-Q3	6.0-17.8	14.9-40.1	7.0-23.3	
Osteocalcin (ng/ml) at 10 Months	n	2	1	4	0,725
	Median	10,2	16,8	14,3	
	Q1-Q3	2.8-17.7	16.8-16.8	6.3-23.9	
Osteocalcin (ng/ml) at 12 Months	n	1	1	0	
	Median	20,8	13,3	.	
	Q1-Q3	20.8-20.8	13.3-13.3	.-.	
	**PINP (pg/ml)**				
PINP (pg/ml) at baseline	n	8	8	8	0,310
	Median	415,2	578	422,4	
	Q1-Q3	139.5-702.4	512.4-1005	272.6-757.3	
PINP (pg/ml) at 2 Months	n	6	4	7	0,463
	Median	467,8	743	229,5	
	Q1-Q3	333.2-569.4	462.8-1072	139.5-1020	
PINP (pg/ml) at 4 Months	n	3	3	5	0,314
	Median	459,5	777,5	439,2	
	Q1-Q3	235.2-514.2	490.2-1567	411.6-601.4	
PINP (pg/ml) at 6 Months	n	2	3	4	0,705
	Median	442,3	698,7	431,2	
	Q1-Q3	95.6-789.0	439.3-1694	408.7-1249	
PINP (pg/ml) at 8 Months	n	2	2	4	0,570
	Median	601,7	1461	1265	
	Q1-Q3	198.9-1004	765.5-2156	461.1-2747	
PINP (pg/ml) at 10 Months	n	2	1	4	0,923
	Median	854,4	652	1563	

	Q1-Q3	447.6-1261	652.0-652.0	447.4-3109	
PINP (pg/ml) at 12 Months	n	1	1	0	
	Median	1284	701,3	.	
	Q1-Q3	1284-1284	701.3-701.3	.-.	
	**CTX(ng/ml)**				
CTX(ng/ml) at baseline	n	8	8	8	0,746
	Median	0,6	0,8	0,8	
	Q1-Q3	0.2-0.8	0.4-1.0	0.3-1.0	
CTX(ng/ml) at 2 Months	n	6	4	7	0,770
	Median	0,3	0,5	0,5	
	Q1-Q3	0.2-0.5	0.3-0.7	0.1-0.6	
CTX(ng/ml) at 4 Months	n	3	3	5	0,712
	Median	0,2	0,3	0,3	
	Q1-Q3	0.1-0.5	0.2-0.7	0.3-0.3	
CTX(ng/ml) at 6 Months	n	2	3	4	0,574
	Median	0,3	0,3	0,2	
	Q1-Q3	0.1-0.4	0.2-0.7	0.2-0.4	
CTX(ng/ml) at 8 Months	n	2	2	4	0,673
	Median	0,2	0,3	0,1	
	Q1-Q3	0.2-0.3	0.1-0.5	0.1-0.3	
CTX(ng/ml) at 10 Months	n	2	1	4	0,158
	Median	0,3	0,4	0,1	
	Q1-Q3	0.2-0.3	0.4-0.4	0.1-0.2	
CTX(ng/ml) at 12 Months	n	1	1	0	
	Median	0,2	0,4	.	
	Q1-Q3	0.2-0.2	0.4-0.4	.-.	
	**TRACP-5B (U/L)**				
TRACP-5B (U/L) at baseline	n	8	8	8	0,564
	Median	2	3,4	3,6	
	Q1-Q3	1.4-3.8	1.9-4.5	2.0-3.9	
TRACP-5B (U/L) at 2 Months	n	6	4	7	0,207
	Median	1,3	2,1	1,8	
	Q1-Q3	0.7-1.9	1.9-2.6	1.0-2.4	

TRACP-5B (U/L) at 4 Months	n	3	3	5	0,157
	Median	0,8	2	1,2	
	Q1-Q3	0.5-1.9	1.3-2.2	1.0-1.2	
TRACP-5B (U/L) at 6 Months	n	2	3	4	**0,047**
	Median	1,8	1,9	1	
	Q1-Q3	1.7-1.9	1.3-15.9	0.9-1.1	
TRACP-5B (U/L) at 8 Months	n	2	2	4	0,125
	Median	0,8	1,6	1	
	Q1-Q3	0.6-0.9	1.0-2.2	0.9-1.0	
TRACP-5B (U/L) at 10 Months	n	1	1	3	0,344
	Median	0,9	2	0,9	
	Q1-Q3	0.9-0.9	2.0-2.0	0.8-0.9	
TRACP-5B (U/L) at 12 Months	n	1	1	0	
	Median	0,9	1,8	.	
	Q1-Q3	0.9-0.9	1.8-1.8	.-.	
	**RANKL (pmol/L)**				
RANKL (pmol/L) at baseline	n	8	8	8	0,846
	Median	0,2	0,2	0,4	
	Q1-Q3	0.2-0.4	0.1-0.4	0.1-0.5	
RANKL (pmol/L) at 2 Months	n	6	4	7	0,560
	Median	0,2	0,2	0,2	
	Q1-Q3	0.1-0.2	0.1-0.3	0.1-0.2	
RANKL (pmol/L) at 4 Months	n	3	3	5	0,132
	Median	0,1	0,1	0,1	
	Q1-Q3	0.1-0.2	0.1-0.2	0.1-0.1	
RANKL (pmol/L) at 6 Months	n	2	3	4	0,398
	Median	0,1	0,1	0,1	
	Q1-Q3	0.1-0.1	0.1-0.2	0.1-0.1	
RANKL (pmol/L) at 8 Months	n	2	2	4	0,293
	Median	0,1	0,1	0,1	
	Q1-Q3	0.1-0.1	0.1-0.2	0.0-0.1	
RANKL (pmol/L) at 10 Months	n	2	1	4	0,235
	Median	0,1	0,3	0	

	Q1-Q3	0.1-0.1	0.3-0.3	0.0-0.1	
RANKL (pmol/L) at 12 Months	n	1	1	0	
	Median	0,1	0,2	.	
	Q1-Q3	0.1-0.1	0.2-0.2	.-.	
	**RANKL/OPG ratio**				
RANKL/OPG ratio at baseline	n	8	8	8	0,907
	Median	0,1	0,1	0,1	
	Q1-Q3	0.0-0.1	0.0-0.1	0.0-0.1	
RANKL/OPG ratio at 2 Months	n	6	4	7	0,318
	Median	0	0	0	
	Q1-Q3	0.0-0.0	0.0-0.1	0.0-0.0	
RANKL/OPG ratio at 4 Months	n	3	3	5	0,132
	Median	0	0	0	
	Q1-Q3	0.0-0.0	0.0-0.0	0.0-0.0	
RANKL/OPG ratio at 6 Months	n	2	3	4	0,705
	Median	0	0	0	
	Q1-Q3	0.0-0.0	0.0-0.0	0.0-0.0	
RANKL/OPG ratio at 8 Months	n	2	2	4	0,346
	Median	0	0	0	
	Q1-Q3	0.0-0.0	0.0-0.0	0.0-0.0	
RANKL/OPG ratio at 10 Months	n	2	1	4	0,325
	Median	0	0,1	0	
	Q1-Q3	0.0-0.0	0.1-0.1	0.0-0.0	
RANKL/OPG ratio at 12 Months	n	1	1	0	
	Median	0	0	.	
	Q1-Q3	0.0-0.0	0.0-0.0	.-.	
	**SOST (pmol/L)**				
SOST (pmol/L) at baseline	n	8	8	8	0,512
	Median	58,7	46	39,8	
	Q1-Q3	43.1-68.1	37.8-65.2	35.6-63.7	
SOST (pmol/L) at 2 Months	n	6	4	7	0,590
	Median	37,7	34,9	40,7	
	Q1-Q3	34.0-48.3	27.6-38.1	27.9-49.7	

SOST (pmol/L) at 4 Months	n	3	3	5	0,167
	Median	36,7	45,8	25	
	Q1-Q3	33.2-43.3	32.8-98.7	22.8-29.7	
SOST (pmol/L) at 6 Months	n	2	3	4	0,667
	Median	27,4	48,1	59,2	
	Q1-Q3	22.9-31.8	25.8-63.1	23.5-121.3	
SOST (pmol/L) at 8 Months	n	2	2	4	>0.999
	Median	33,9	28	42,3	
	Q1-Q3	23.5-44.3	26.2-29.9	20.8-88.3	
SOST (pmol/L) at 10 Months	n	2	1	4	0,923
	Median	42,9	36,9	30,4	
	Q1-Q3	21.1-64.7	36.9-36.9	19.8-61.3	
SOST (pmol/L) at 12 Months	n	1	1	0	
	Median	20	35,7	.	
	Q1-Q3	20.0-20.0	35.7-35.7	.-.	
	**Dkk1 (pmol/L)**				
Dkk1 (pmol/L) at baseline	n	8	8	8	0,175
	Median	36,5	35	52,8	
	Q1-Q3	23.8-46.8	23.1-59.8	42.5-78.3	
Dkk1 (pmol/L) at 2 Months	n	6	4	7	0,243
	Median	26,7	32,8	58,4	
	Q1-Q3	24.1-62.5	19.7-46.1	31.4-72.2	
Dkk1 (pmol/L) at 4 Months	n	3	3	5	0,761
	Median	58,4	27,8	33,7	
	Q1-Q3	14.6-63.3	15.9-57.5	22.8-46.5	
Dkk1 (pmol/L) at 6 Months	n	2	3	4	0,962
	Median	36,8	34	38,3	
	Q1-Q3	21.2-52.4	9.7-54.8	34.5-44.4	
Dkk1 (pmol/L) at 8 Months	n	2	2	4	0,408
	Median	35,2	17,1	30,4	
	Q1-Q3	19.2-51.3	5.9-28.2	26.8-32.7	
Dkk1 (pmol/L) at 10 Months	n	2	1	4	0,923
	Median	18,9	26,1	23,6	

	Q1-Q3	9.1-28.7	26.1-26.1	12.3-34.9	
Dkk1 (pmol/L) at 12 Months	n	1	1	0	
	Median	8,4	20,4	.	
	Q1-Q3	8.4-8.4	20.4-20.4	.-.	
	**activin-A (pg/ml)**				
activin-A (pg/ml) at baseline	n	8	8	8	0,595
	Median	581,6	798,5	644,7	
	Q1-Q3	389.2-703.4	553.9-908.9	514.9-833.2	
activin-A (pg/ml) at 2 Months	n	6	4	7	**0,041**
	Median	462,4	300,5	562,2	
	Q1-Q3	436.9-488.4	231.1-391.6	390.4-786.7	
activin-A (pg/ml) at 4 Months	n	3	3	5	0,976
	Median	412,5	418,7	468,3	
	Q1-Q3	355.4-580.9	334.5-519.6	223.6-492.5	
activin-A (pg/ml) at 6 Months	n	2	3	4	0,432
	Median	344,2	504,5	372,8	
	Q1-Q3	300.0-388.3	372.6-695.8	290.9-486.3	
activin-A (pg/ml) at 8 Months	n	2	2	4	0,264
	Median	289,6	488,5	368,7	
	Q1-Q3	213.5-365.7	439.0-537.9	275.4-700.5	
activin-A (pg/ml) at 10 Months	n	2	1	4	0,601
	Median	360,2	280,5	353,2	
	Q1-Q3	357.5-362.8	280.5-280.5	242.4-445.1	
activin-A (pg/ml) at 12 Months	n	1	1	0	
	Median	256,8	318,2	.	
	Q1-Q3	256.8-256.8	318.2-318.2	.-.	
	**CCL3 (ng/ml)**				
CCL3 (ng/ml) at baseline	n	8	8	8	0,388
	Median	79,2	80,1	72,4	
	Q1-Q3	42.1-101.3	73.9-96.8	36.2-84.2	
CCL3 (ng/ml) at 2 Months	n	6	4	7	0,849
	Median	76,8	55,7	71,2	
	Q1-Q3	39.0-95.5	27.2-85.1	45.7-89.4	

CCL3 (ng/ml) at 4 Months	n	3	3	5	0,886
	Median	63,3	68	68	
	Q1-Q3	32.8-82.9	34.3-138.2	61.3-69.6	
CCL3 (ng/ml) at 6 Months	n	2	3	4	0,678
	Median	69,3	61,3	61,7	
	Q1-Q3	67.4-71.1	6.3-74.3	57.6-67.6	
CCL3 (ng/ml) at 8 Months	n	2	2	4	0,368
	Median	58,9	31,9	64	
	Q1-Q3	54.3-63.5	8.7-55.1	41.1-77.9	
CCL3 (ng/ml) at 10 Months	n	2	1	4	0,248
	Median	58,4	9,1	32,4	
	Q1-Q3	56.0-60.8	9.1-9.1	8.8-54.3	
CCL3 (ng/ml) at 12 Months	n	1	1	0	
	Median	64,3	3,9	.	
	Q1-Q3	64.3-64.3	3.9-3.9	.-.	

[a] Kruskal-Walli test

Table S4. Biomarker values at each timepoint, by R-ISS stage at diagnosis

		R-ISS at diagnosis			
		I	II	III	
		n (%)	n (%)	n (%)	p-value[a]
	**bALP (µg/L)**				
bALP (µg/L) at baseline	n	7	11	6	0,790
	Median	11,3	11,1	10,4	
	Q1-Q3	8.9-14.0	9.3-11.7	8.3-11.5	
bALP (µg/L) at 2 Months	n	5	7	5	0,305
	Median	8	12,9	12,4	
	Q1-Q3	7.6-12.1	9.4-19.5	11.7-12.8	
bALP (µg/L) at 4 Months	n	3	5	3	0,157
	Median	9,1	11,9	12,9	
	Q1-Q3	7.7-11.3	10.8-14.1	11.6-14.4	
bALP (µg/L) at 6 Months	n	2	5	2	0,337
	Median	10,9	11,6	16,7	
	Q1-Q3	8.1-13.6	7.1-14.8	13.7-19.7	
bALP (µg/L) at 8 Months	n	2	4	2	0,264
	Median	9,4	11,9	17,4	
	Q1-Q3	3.8-15.0	6.2-18.3	16.9-18.0	
bALP (µg/L) at 10 Months	n	2	3	2	0,153
	Median	11	9,8	19	
	Q1-Q3	7.0-15.0	5.9-15.8	18.1-20.0	
bALP (µg/L) at 12 Months	n	1	1	0	
	Median	14,5	19,7	.	
	Q1-Q3	14.5-14.5	19.7-19.7	.-.	
	**Osteocalcin (ng/ml)**				
Osteocalcin (ng/ml) at baseline	n	7	11	6	0,652
	Median	10,8	9,2	8,3	
	Q1-Q3	5.8-11.4	2.9-10.8	7.4-18.9	
Osteocalcin (ng/ml) at 2 Months	n	5	7	5	0,668
	Median	14,1	10,9	9,4	
	Q1-Q3	10.5-14.9	2.0-14.1	8.8-9.4	

Osteocalcin (ng/ml) at 4 Months	n	3	5	3	0,257
	Median	8,6	17,7	12,3	
	Q1-Q3	1.0-17.2	12.4-19.2	11.2-19.7	
Osteocalcin (ng/ml) at 6 Months	n	2	5	2	0,549
	Median	10,1	12,8	19,7	
	Q1-Q3	4.0-16.2	11.1-18.9	13.9-25.4	
Osteocalcin (ng/ml) at 8 Months	n	2	4	2	0,607
	Median	11,9	11,7	23,3	
	Q1-Q3	6.0-17.8	7.0-27.5	16.9-29.8	
Osteocalcin (ng/ml) at 10 Months	n	2	3	2	0,153
	Median	10,2	8,9	23,9	
	Q1-Q3	2.8-17.7	3.8-16.8	19.7-28.2	
Osteocalcin (ng/ml) at 12 Months	n	1	1	0	
	Median	20,8	13,3	.	
	Q1-Q3	20.8-20.8	13.3-13.3	.-.	
	**PINP (pg/ml)**				
PINP (pg/ml) at baseline	n	7	11	6	0,298
	Median	362,7	572,8	419,7	
	Q1-Q3	115.5-750.7	467.8-1268	253.1-733.1	
PINP (pg/ml) at 2 Months	n	5	7	5	0,992
	Median	384,9	550,8	384,2	
	Q1-Q3	333.2-569.4	214.8-775.3	229.5-1020	
PINP (pg/ml) at 4 Months	n	3	5	3	0,469
	Median	459,5	490,2	601,4	
	Q1-Q3	235.2-514.2	411.6-777.5	439.2-1897	
PINP (pg/ml) at 6 Months	n	2	5	2	0,766
	Median	442,3	442,8	1238	
	Q1-Q3	95.6-789.0	439.3-698.7	419.7-2056	
PINP (pg/ml) at 8 Months	n	2	4	2	0,570
	Median	601,7	1461	1265	
	Q1-Q3	198.9-1004	573.3-2831	541.1-1988	
PINP (pg/ml) at 10 Months	n	2	3	2	0,898
	Median	854,4	652	1563	

	Q1-Q3	447.6-1261	335.8-3650	559.0-2567	
PINP (pg/ml) at 12 Months	n	1	1	0	
	Median	1284	701,3	.	
	Q1-Q3	1284-1284	701.3-701.3	.-.	
	**CTX(ng/ml)**				
CTX(ng/ml) at baseline	n	7	11	6	0,294
	Median	0,6	0,8	0,9	
	Q1-Q3	0.2-0.8	0.2-0.9	0.7-1.0	
CTX(ng/ml) at 2 Months	n	5	7	5	0,379
	Median	0,3	0,4	0,6	
	Q1-Q3	0.2-0.3	0.1-0.7	0.5-0.6	
CTX(ng/ml) at 4 Months	n	3	5	3	0,344
	Median	0,2	0,3	0,3	
	Q1-Q3	0.1-0.5	0.2-0.3	0.3-0.7	
CTX(ng/ml) at 6 Months	n	2	5	2	0,835
	Median	0,3	0,2	0,4	
	Q1-Q3	0.1-0.4	0.2-0.3	0.2-0.6	
CTX(ng/ml) at 8 Months	n	2	4	2	0,346
	Median	0,2	0,1	0,3	
	Q1-Q3	0.2-0.3	0.1-0.3	0.1-0.5	
CTX(ng/ml) at 10 Months	n	2	3	2	0,700
	Median	0,3	0,1	0,2	
	Q1-Q3	0.2-0.3	0.1-0.4	0.1-0.2	
CTX(ng/ml) at 12 Months	n	1	1	0	
	Median	0,2	0,4	.	
	Q1-Q3	0.2-0.2	0.4-0.4	.-.	
	**TRACP-5B (U/L)**				
TRACP-5B (U/L) at baseline	n	7	11	6	0,317
	Median	1,8	3,3	3,7	
	Q1-Q3	1.1-3.6	1.9-4.3	3.5-4.0	
TRACP-5B (U/L) at 2 Months	n	5	7	5	0,285
	Median	0,8	1,9	1,9	
	Q1-Q3	0.7-1.9	1.7-2.1	1.7-2.4	

TRACP-5B (U/L) at 4 Months	n	3	5	3	0,466
	Median	0,8	1,3	1,2	
	Q1-Q3	0.5-1.9	1.0-2.0	1.2-2.1	
TRACP-5B (U/L) at 6 Months	n	2	5	2	0,429
	Median	1,8	1,3	1,1	
	Q1-Q3	1.7-1.9	1.1-1.9	1.0-1.2	
TRACP-5B (U/L) at 8 Months	n	2	4	2	0,210
	Median	0,8	1,1	1	
	Q1-Q3	0.6-0.9	0.9-1.6	1.0-1.0	
TRACP-5B (U/L) at 10 Months	n	1	2	2	0,741
	Median	0,9	1,4	0,9	
	Q1-Q3	0.9-0.9	0.8-2.0	0.9-0.9	
TRACP-5B (U/L) at 12 Months	n	1	1	0	
	Median	0,9	1,8	.	
	Q1-Q3	0.9-0.9	1.8-1.8	.-.	
	**RANKL (pmol/L)**				
RANKL (pmol/L) at baseline	n	7	11	6	0,221
	Median	0,2	0,2	0,4	
	Q1-Q3	0.2-0.3	0.1-0.4	0.3-0.5	
RANKL (pmol/L) at 2 Months	n	5	7	5	0,679
	Median	0,2	0,2	0,2	
	Q1-Q3	0.1-0.2	0.1-0.2	0.2-0.2	
RANKL (pmol/L) at 4 Months	n	3	5	3	0,120
	Median	0,1	0,1	0,1	
	Q1-Q3	0.1-0.2	0.1-0.1	0.1-0.1	
RANKL (pmol/L) at 6 Months	n	2	5	2	0,076
	Median	0,1	0,1	0,1	
	Q1-Q3	0.1-0.1	0.1-0.2	0.1-0.1	
RANKL (pmol/L) at 8 Months	n	2	4	2	0,127
	Median	0,1	0,1	0	
	Q1-Q3	0.1-0.1	0.1-0.2	0.0-0.0	
RANKL (pmol/L) at 10 Months	n	2	3	2	0,140
	Median	0,1	0,2	0	

	Q1-Q3	0.1-0.1	0.1-0.3	0.0-0.0	
RANKL (pmol/L) at 12 Months	n	1	1	0	
	Median	0,1	0,2	.	
	Q1-Q3	0.1-0.1	0.2-0.2	.-.	
	**RANKL/OPG ratio**				
RANKL/OPG ratio at baseline	n	7	11	6	0,223
	Median	0,1	0,1	0,1	
	Q1-Q3	0.0-0.1	0.0-0.1	0.1-0.1	
RANKL/OPG ratio at 2 Months	n	5	7	5	0,676
	Median	0	0	0	
	Q1-Q3	0.0-0.0	0.0-0.1	0.0-0.0	
RANKL/OPG ratio at 4 Months	n	3	5	3	0,074
	Median	0	0	0	
	Q1-Q3	0.0-0.0	0.0-0.0	0.0-0.0	
RANKL/OPG ratio at 6 Months	n	2	5	2	0,102
	Median	0	0	0	
	Q1-Q3	0.0-0.0	0.0-0.0	0.0-0.0	
RANKL/OPG ratio at 8 Months	n	2	4	2	0,135
	Median	0	0	0	
	Q1-Q3	0.0-0.0	0.0-0.0	0.0-0.0	
RANKL/OPG ratio at 10 Months	n	2	3	2	0,069
	Median	0	0	0	
	Q1-Q3	0.0-0.0	0.0-0.1	0.0-0.0	
RANKL/OPG ratio at 12 Months	n	1	1	0	
	Median	0	0	.	
	Q1-Q3	0.0-0.0	0.0-0.0	.-.	
	**SOST (pmol/L)**				
SOST (pmol/L) at baseline	n	7	11	6	0,384
	Median	56,2	50,3	39,8	
	Q1-Q3	41.4-70.6	37.9-66.3	33.2-62.9	
SOST (pmol/L) at 2 Months	n	5	7	5	0,886
	Median	37,2	40,1	29,4	
	Q1-Q3	34.0-38.2	33.8-48.3	27.9-41.7	

SOST (pmol/L) at 4 Months	n	3	5	3	**0,047**
	Median	36,7	45,8	22,8	
	Q1-Q3	33.2-43.3	32.8-77.1	22.3-25.0	
SOST (pmol/L) at 6 Months	n	2	5	2	0,076
	Median	27,4	63,1	23,5	
	Q1-Q3	22.9-31.8	48.1-92.9	21.6-25.5	
SOST (pmol/L) at 8 Months	n	2	4	2	0,105
	Median	33,9	46,7	20,8	
	Q1-Q3	23.5-44.3	28.0-88.3	20.4-21.2	
SOST (pmol/L) at 10 Months	n	2	3	2	0,140
	Median	42,9	40,5	19,8	
	Q1-Q3	21.1-64.7	36.9-82.1	19.3-20.2	
SOST (pmol/L) at 12 Months	n	1	1	0	
	Median	20	35,7	.	
	Q1-Q3	20.0-20.0	35.7-35.7	.-.	
	**Dkk1 (pmol/L)**				
Dkk1 (pmol/L) at baseline	n	7	11	6	0,657
	Median	37,5	38	47	
	Q1-Q3	16.3-52.4	25.1-76.6	42.0-54.7	
Dkk1 (pmol/L) at 2 Months	n	5	7	5	0,606
	Median	29,1	36,9	42	
	Q1-Q3	24.4-62.5	22.7-58.4	31.4-72.2	
Dkk1 (pmol/L) at 4 Months	n	3	5	3	0,549
	Median	58,4	27,8	46,5	
	Q1-Q3	14.6-63.3	18.5-33.7	22.8-62.9	
Dkk1 (pmol/L) at 6 Months	n	2	5	2	1,000
	Median	36,8	37	40,6	
	Q1-Q3	21.2-52.4	34.0-39.7	32.0-49.2	
Dkk1 (pmol/L) at 8 Months	n	2	4	2	0,472
	Median	35,2	26	32,1	
	Q1-Q3	19.2-51.3	14.9-29.6	29.7-34.4	
Dkk1 (pmol/L) at 10 Months	n	2	3	2	0,140
	Median	18,9	16,3	34,9	

	Q1-Q3	9.1-28.7	8.4-26.1	31.0-38.7	
Dkk1 (pmol/L) at 12 Months	n	1	1	0	
	Median	8,4	20,4	.	
	Q1-Q3	8.4-8.4	20.4-20.4	.-.	
	**activin-A (pg/ml)**				
activin-A (pg/ml) at baseline	n	7	11	6	0,553
	Median	548,3	694,3	644,7	
	Q1-Q3	286.2-770.8	505.0-913.5	524.9-745.2	
activin-A (pg/ml) at 2 Months	n	5	7	5	0,208
	Median	447,5	390,4	562,2	
	Q1-Q3	436.9-488.4	280.0-477.3	538.3-627.9	
activin-A (pg/ml) at 4 Months	n	3	5	3	0,338
	Median	412,5	468,3	223,6	
	Q1-Q3	355.4-580.9	418.7-519.6	186.1-492.5	
activin-A (pg/ml) at 6 Months	n	2	5	2	0,318
	Median	344,2	504,5	296,9	
	Q1-Q3	300.0-388.3	372.6-593.8	215.0-378.7	
activin-A (pg/ml) at 8 Months	n	2	4	2	0,069
	Median	289,6	488,5	275,4	
	Q1-Q3	213.5-365.7	428.7-760.3	231.9-319.0	
activin-A (pg/ml) at 10 Months	n	2	3	2	0,292
	Median	360,2	422,7	242,4	
	Q1-Q3	357.5-362.8	280.5-467.5	201.0-283.8	
activin-A (pg/ml) at 12 Months	n	1	1	0	
	Median	256,8	318,2	.	
	Q1-Q3	256.8-256.8	318.2-318.2	.-.	
	**CCL3 (ng/ml)**				
CCL3 (ng/ml) at baseline	n	7	11	6	0,644
	Median	76,6	79	77,1	
	Q1-Q3	22.8-100.9	70.9-98.9	10.2-88.1	
CCL3 (ng/ml) at 2 Months	n	5	7	5	0,836
	Median	70,5	67,4	71,2	
	Q1-Q3	39.0-83.2	44.0-102.7	45.7-76.3	

CCL3 (ng/ml) at 4 Months	n	3	5	3	0,814
	Median	63,3	68	69,6	
	Q1-Q3	32.8-82.9	47.0-68.0	61.3-72.0	
CCL3 (ng/ml) at 6 Months	n	2	5	2	0,340
	Median	69,3	61,2	67,6	
	Q1-Q3	67.4-71.1	54.0-61.3	62.1-73.1	
CCL3 (ng/ml) at 8 Months	n	2	4	2	0,105
	Median	58,9	38,1	77,9	
	Q1-Q3	54.3-63.5	14.9-58.1	66.9-88.9	
CCL3 (ng/ml) at 10 Months	n	2	3	2	0,095
	Median	58,4	9,1	54,3	
	Q1-Q3	56.0-60.8	3.3-14.2	50.7-57.9	
CCL3 (ng/ml) at 12 Months	n	1	1	0	
	Median	64,3	3,9	.	
	Q1-Q3	64.3-64.3	3.9-3.9	.-.	

[a] Kruskal-Wallis test

Table S5. Biomarker values at each timepoint, by ISS stage at Kd initiation

		ISS at Kd initiation			
		**I**	**II**	**III**	
		**n (%)**	**n (%)**	**n (%)**	**p-value[a]**
		**bALP (µg/L)**			
bALP (µg/L) at baseline	n	9	7	8	0,996
	Median	11,1	11,2	10,5	
	Q1-Q3	10.4-11.3	7.8-14.0	9.5-11.6	
bALP (µg/L) at 2 Months	n	9	3	5	0,815
	Median	11,7	9,1	12,8	
	Q1-Q3	9.4-12.9	7.8-19.1	12.4-15.4	
bALP (µg/L) at 4 Months	n	7	2	2	0,842
	Median	11,9	11,4	10,9	
	Q1-Q3	9.1-14.4	11.3-11.6	8.9-12.9	
bALP (µg/L) at 6 Months	n	7	0	2	0,380
	Median	13,6	.	10,3	
	Q1-Q3	8.1-14.8	.-.	6.8-13.7	
bALP (µg/L) at 8 Months	n	6	0	2	0,739
	Median	16	.	11,3	
	Q1-Q3	6.8-18.0	.-.	5.6-16.9	
bALP (µg/L) at 10 Months	n	5	0	2	0,699
	Median	15	.	12	
	Q1-Q3	9.8-15.8	.-.	5.9-18.1	
bALP (µg/L) at 12 Months	n	2	0	0	
	Median	17,1	.	.	
	Q1-Q3	14.5-19.7	.-.	.-.	
		**Osteocalcin (ng/ml)**			
Osteocalcin (ng/ml) at baseline	n	9	7	8	0,642
	Median	9,2	7,5	10,5	
	Q1-Q3	6.4-10.8	2.9-10.8	4.9-18.2	
Osteocalcin (ng/ml) at 2 Months	n	9	3	5	0,348
	Median	12,2	9,4	9,4	
	Q1-Q3	10.5-14.9	3.5-10.5	5.8-14.1	

Osteocalcin (ng/ml) at 4 Months	n	7	2	2	0,363
	Median	17,2	6,6	14,8	
	Q1-Q3	11.2-19.2	1.0-12.3	9.9-19.7	
Osteocalcin (ng/ml) at 6 Months	n	7	0	2	0,770
	Median	13,9	.	18,2	
	Q1-Q3	10.8-18.9	.-.	11.1-25.4	
Osteocalcin (ng/ml) at 8 Months	n	6	0	2	0,739
	Median	15,9	.	19,1	
	Q1-Q3	6.0-17.8	.-.	8.5-29.8	
Osteocalcin (ng/ml) at 10 Months	n	5	0	2	0,699
	Median	16,8	.	16	
	Q1-Q3	8.9-17.7	.-.	3.8-28.2	
Osteocalcin (ng/ml) at 12 Months	n	2	0	0	
	Median	17,1	.	.	
	Q1-Q3	13.3-20.8	.-.	.-.	
	**PINP (pg/ml)**				
PINP (pg/ml) at baseline	n	9	7	8	0,390
	Median	654,2	537	330,1	
	Q1-Q3	467.8-1268	310.5-750.7	234.5-644.7	
PINP (pg/ml) at 2 Months	n	9	3	5	0,293
	Median	710,7	384,2	229,5	
	Q1-Q3	333.2-779.6	214.8-569.4	139.5-384.9	
PINP (pg/ml) at 4 Months	n	7	2	2	0,450
	Median	490,2	557,8	425,4	
	Q1-Q3	278.5-1567	514.2-601.4	411.6-439.2	
PINP (pg/ml) at 6 Months	n	7	0	2	0,143
	Median	698,7	.	408,7	
	Q1-Q3	439.3-1694	.-.	397.8-419.7	
PINP (pg/ml) at 8 Months	n	6	0	2	0,182
	Median	1496	.	461,1	
	Q1-Q3	765.5-2156	.-.	381.1-541.1	
PINP (pg/ml) at 10 Months	n	5	0	2	0,121
	Median	1261	.	447,4	

	Q1-Q3	652.0-2567	.-.	335.8-559.0	
PINP (pg/ml) at 12 Months	n	2	0	0	
	Median	992,5	.	.	
	Q1-Q3	701.3-1284	.-.	.-.	
		**CTX(ng/ml)**			
CTX(ng/ml) at baseline	n	9	7	8	0,772
	Median	0,8	0,9	0,7	
	Q1-Q3	0.2-0.9	0.2-1.2	0.4-0.9	
CTX(ng/ml) at 2 Months	n	9	3	5	0,644
	Median	0,5	0,3	0,2	
	Q1-Q3	0.3-0.6	0.1-0.5	0.1-0.6	
CTX(ng/ml) at 4 Months	n	7	2	2	0,618
	Median	0,3	0,3	0,5	
	Q1-Q3	0.2-0.5	0.2-0.3	0.3-0.7	
CTX(ng/ml) at 6 Months	n	7	0	2	0,380
	Median	0,2	.	0,4	
	Q1-Q3	0.1-0.4	.-.	0.2-0.6	
CTX(ng/ml) at 8 Months	n	6	0	2	0,739
	Median	0,1	.	0,3	
	Q1-Q3	0.1-0.3	.-.	0.1-0.5	
CTX(ng/ml) at 10 Months	n	5	0	2	0,699
	Median	0,2	.	0,2	
	Q1-Q3	0.1-0.3	.-.	0.1-0.2	
CTX(ng/ml) at 12 Months	n	2	0	0	
	Median	0,3	.	.	
	Q1-Q3	0.2-0.4	.-.	.-.	
		**TRACP-5B (U/L)**			
TRACP-5B (U/L) at baseline	n	9	7	8	0,629
	Median	2,2	3,6	3,6	
	Q1-Q3	1.5-3.9	1.9-4.8	2.0-3.7	
TRACP-5B (U/L) at 2 Months	n	9	3	5	0,241
	Median	2	1,9	1,7	
	Q1-Q3	1.0-2.1	1.7-2.4	0.6-1.8	

TRACP-5B (U/L) at 4 Months	n	7	2	2	0,299
	Median	1,2	2	1	
	Q1-Q3	0.8-2.0	1.9-2.1	0.7-1.2	
TRACP-5B (U/L) at 6 Months	n	7	0	2	0,143
	Median	1,7	.	1	
	Q1-Q3	1.1-1.9	.-.	0.9-1.2	
TRACP-5B (U/L) at 8 Months	n	6	0	2	0,505
	Median	1	.	1	
	Q1-Q3	0.8-1.0	.-.	1.0-1.1	
TRACP-5B (U/L) at 10 Months	n	3	0	2	0,564
	Median	0,9	.	0,8	
	Q1-Q3	0.9-2.0	.-.	0.8-0.9	
TRACP-5B (U/L) at 12 Months	n	2	0	0	
	Median	1,3	.	.	
	Q1-Q3	0.9-1.8	.-.	.-.	
	**RANKL (pmol/L)**				
RANKL (pmol/L) at baseline	n	9	7	8	0,834
	Median	0,3	0,2	0,3	
	Q1-Q3	0.2-0.4	0.1-0.4	0.2-0.5	
RANKL (pmol/L) at 2 Months	n	9	3	5	0,736
	Median	0,2	0,2	0,2	
	Q1-Q3	0.1-0.2	0.1-0.2	0.1-0.2	
RANKL (pmol/L) at 4 Months	n	7	2	2	0,718
	Median	0,1	0,1	0,1	
	Q1-Q3	0.1-0.2	0.1-0.1	0.1-0.1	
RANKL (pmol/L) at 6 Months	n	7	0	2	0,380
	Median	0,1	.	0,1	
	Q1-Q3	0.1-0.2	.-.	0.1-0.1	
RANKL (pmol/L) at 8 Months	n	6	0	2	>0.999
	Median	0,1	.	0,1	
	Q1-Q3	0.1-0.1	.-.	0.0-0.1	
RANKL (pmol/L) at 10 Months	n	5	0	2	0,699
	Median	0,1	.	0,1	

	Q1-Q3	0.1-0.1	.-.	0.0-0.2	
RANKL (pmol/L) at 12 Months	n	2	0	0	
	Median	0,1	.	.	
	Q1-Q3	0.1-0.2	.-.	.-.	
	**RANKL/OPG ratio**				
RANKL/OPG ratio at baseline	n	9	7	8	0,772
	Median	0,1	0,1	0,1	
	Q1-Q3	0.0-0.1	0.0-0.1	0.0-0.1	
RANKL/OPG ratio at 2 Months	n	9	3	5	0,907
	Median	0	0	0	
	Q1-Q3	0.0-0.0	0.0-0.1	0.0-0.0	
RANKL/OPG ratio at 4 Months	n	7	2	2	0,751
	Median	0	0	0	
	Q1-Q3	0.0-0.0	0.0-0.0	0.0-0.0	
RANKL/OPG ratio at 6 Months	n	7	0	2	0,242
	Median	0	.	0	
	Q1-Q3	0.0-0.0	.-.	0.0-0.0	
RANKL/OPG ratio at 8 Months	n	6	0	2	0,739
	Median	0	.	0	
	Q1-Q3	0.0-0.0	.-.	0.0-0.0	
RANKL/OPG ratio at 10 Months	n	5	0	2	0,699
	Median	0	.	0	
	Q1-Q3	0.0-0.0	.-.	0.0-0.0	
RANKL/OPG ratio at 12 Months	n	2	0	0	
	Median	0	.	.	
	Q1-Q3	0.0-0.0	.-.	.-.	
	**SOST (pmol/L)**				
SOST (pmol/L) at baseline	n	9	7	8	0,932
	Median	50,3	56,2	40,8	
	Q1-Q3	37.9-66.3	37.7-65.6	38.8-64.4	
SOST (pmol/L) at 2 Months	n	9	3	5	0,761
	Median	37,2	38,2	29,4	
	Q1-Q3	34.0-48.3	33.8-41.7	22.0-40.7	

SOST (pmol/L) at 4 Months	n	7	2	2	0,212
	Median	36,7	34,1	26	
	Q1-Q3	32.8-77.1	25.0-43.3	22.3-29.7	
SOST (pmol/L) at 6 Months	n	7	0	2	0,770
	Median	31,8	.	57,2	
	Q1-Q3	25.5-63.1	.-.	21.6-92.9	
SOST (pmol/L) at 8 Months	n	6	0	2	>0.999
	Median	28	.	66,8	
	Q1-Q3	23.5-44.3	.-.	20.4-113.2	
SOST (pmol/L) at 10 Months	n	5	0	2	0,439
	Median	36,9	.	29,9	
	Q1-Q3	21.1-64.7	.-.	19.3-40.5	
SOST (pmol/L) at 12 Months	n	2	0	0	
	Median	27,8	.	.	
	Q1-Q3	20.0-35.7	.-.	.-.	
	**Dkk1 (pmol/L)**				
Dkk1 (pmol/L) at baseline	n	9	7	8	0,258
	Median	54,7	35,5	39,7	
	Q1-Q3	38.0-76.6	21.2-46.8	20.7-47.0	
Dkk1 (pmol/L) at 2 Months	n	9	3	5	0,731
	Median	42	24,4	31,4	
	Q1-Q3	29.1-58.4	10.8-76.2	26.9-67.5	
Dkk1 (pmol/L) at 4 Months	n	7	2	2	0,181
	Median	33,7	60,7	20,6	
	Q1-Q3	15.9-57.5	58.4-62.9	18.5-22.8	
Dkk1 (pmol/L) at 6 Months	n	7	0	2	0,558
	Median	39,7	.	34,5	
	Q1-Q3	21.2-52.4	.-.	32.0-37.0	
Dkk1 (pmol/L) at 8 Months	n	6	0	2	0,739
	Median	29	.	29,1	
	Q1-Q3	19.2-31.0	.-.	23.8-34.4	
Dkk1 (pmol/L) at 10 Months	n	5	0	2	0,699
	Median	26,1	.	19,7	

	Q1-Q3	16.3-28.7	.-.	8.4-31.0	
Dkk1 (pmol/L) at 12 Months	n	2	0	0	
	Median	14,4	.	.	
	Q1-Q3	8.4-20.4	.-.	.-.	
	**activin-A (pg/ml)**				
activin-A (pg/ml) at baseline	n	9	7	8	0,829
	Median	636,1	694,3	620,2	
	Q1-Q3	524.9-913.5	548.3-902.7	349.3-833.2	
activin-A (pg/ml) at 2 Months	n	9	3	5	0,143
	Median	436,9	517,7	562,2	
	Q1-Q3	315.5-462.2	321.0-538.3	488.4-627.9	
activin-A (pg/ml) at 4 Months	n	7	2	2	0,254
	Median	412,5	383,5	698,6	
	Q1-Q3	334.5-468.3	186.1-580.9	492.5-904.7	
activin-A (pg/ml) at 6 Months	n	7	0	2	0,380
	Median	372,6	.	486,3	
	Q1-Q3	300.0-504.5	.-.	378.7-593.8	
activin-A (pg/ml) at 8 Months	n	6	0	2	0,739
	Median	392	.	607,3	
	Q1-Q3	319.0-439.0	.-.	231.9-982.7	
activin-A (pg/ml) at 10 Months	n	5	0	2	0,699
	Median	357,5	.	353,2	
	Q1-Q3	280.5-362.8	.-.	283.8-422.7	
activin-A (pg/ml) at 12 Months	n	2	0	0	
	Median	287,5	.	.	
	Q1-Q3	256.8-318.2	.-.	.-.	
	**CCL3 (ng/ml)**				
CCL3 (ng/ml) at baseline	n	9	7	8	0,395
	Median	76,6	81,3	70,6	
	Q1-Q3	72.4-98.9	22.8-105.8	32.2-84.2	
CCL3 (ng/ml) at 2 Months	n	9	3	5	0,106
	Median	83,2	44	45,7	
	Q1-Q3	70.5-97.0	11.1-76.3	39.0-61.2	

CCL3 (ng/ml) at 4 Months	n	7	2	2	0,239
	Median	68	47	59,5	
	Q1-Q3	63.3-82.9	32.8-61.3	47.0-72.0	
CCL3 (ng/ml) at 6 Months	n	7	0	2	0,770
	Median	62,1	.	67,2	
	Q1-Q3	54.0-71.1	.-.	61.2-73.1	
CCL3 (ng/ml) at 8 Months	n	6	0	2	0,739
	Median	58,1	.	55	
	Q1-Q3	54.3-63.5	.-.	21.1-88.9	
CCL3 (ng/ml) at 10 Months	n	5	0	2	0,245
	Median	56	.	27	
	Q1-Q3	14.2-57.9	.-.	3.3-50.7	
CCL3 (ng/ml) at 12 Months	n	2	0	0	
	Median	34,1	.	.	
	Q1-Q3	3.9-64.3	.-.	.-.	

[a] Kruskal-Wallis test

Table S6. Biomarker values at each timepoint, by R-ISS stage at Kd initiation

		R-ISS at Kd initiation			
		**I**	**II**	**III**	
		**n (%)**	**n (%)**	**n (%)**	**p-value[a]**
		**bALP (µg/L)**			
bALP (µg/L) at baseline	n	5	12	7	0,559
	Median	11,3	10,7	10,5	
	Q1-Q3	10.7-11.7	8.1-11.5	10.0-11.7	
bALP (µg/L) at 2 Months	n	4	8	5	0,403
	Median	12,5	9,3	12,8	
	Q1-Q3	9.8-16.0	7.9-14.8	12.4-15.4	
bALP (µg/L) at 4 Months	n	2	6	3	0,761
	Median	12,7	11,2	12,9	
	Q1-Q3	11.3-14.1	9.1-11.9	8.9-14.4	
bALP (µg/L) at 6 Months	n	2	4	3	0,555
	Median	14,2	9,8	13,7	
	Q1-Q3	13.6-14.8	7.6-13.2	6.8-19.7	
bALP (µg/L) at 8 Months	n	2	3	3	0,368
	Median	17,3	6,8	16,9	
	Q1-Q3	15.0-19.6	3.8-16.9	5.6-18.0	
bALP (µg/L) at 10 Months	n	1	3	3	0,751
	Median	15	9,8	18,1	
	Q1-Q3	15.0-15.0	7.0-15.8	5.9-20.0	
bALP (µg/L) at 12 Months	n	1	1	0	
	Median	14,5	19,7	.	
	Q1-Q3	14.5-14.5	19.7-19.7	.-.	
		**Osteocalcin (ng/ml)**			
Osteocalcin (ng/ml) at baseline	n	5	12	7	0,651
	Median	9,9	8,3	9,1	
	Q1-Q3	6.6-11.2	4.0-10.8	3.9-18.9	
Osteocalcin (ng/ml) at 2 Months	n	4	8	5	0,449
	Median	13,2	10,7	8,8	
	Q1-Q3	7.9-14.5	10.0-16.0	5.8-9.4	

Osteocalcin (ng/ml) at 4 Months	n	2	6	3	0,886
	Median	10,1	14,8	11,2	
	Q1-Q3	1.0-19.2	12.3-17.7	9.9-19.7	
Osteocalcin (ng/ml) at 6 Months	n	2	4	3	0,233
	Median	21,4	11,8	13,9	
	Q1-Q3	16.2-26.7	7.4-15.8	11.1-25.4	
Osteocalcin (ng/ml) at 8 Months	n	2	3	3	0,103
	Median	29	6	16,9	
	Q1-Q3	17.8-40.1	5.6-14.9	8.5-29.8	
Osteocalcin (ng/ml) at 10 Months	n	1	3	3	0,368
	Median	17,7	8,9	19,7	
	Q1-Q3	17.7-17.7	2.8-16.8	3.8-28.2	
Osteocalcin (ng/ml) at 12 Months	n	1	1	0	
	Median	20,8	13,3	.	
	Q1-Q3	20.8-20.8	13.3-13.3	.-.	
	**PINP (pg/ml)**				
PINP (pg/ml) at baseline	n	5	12	7	0,899
	Median	654,2	512,4	547,4	
	Q1-Q3	362.7-780.4	301.3-667.0	253.1-742.1	
PINP (pg/ml) at 2 Months	n	4	8	5	0,235
	Median	674,5	358,7	229,5	
	Q1-Q3	477.1-1074	220.6-630.7	139.5-1020	
PINP (pg/ml) at 4 Months	n	2	6	3	0,469
	Median	1041	474,8	439,2	
	Q1-Q3	514.2-1567	278.5-601.4	411.6-1897	
PINP (pg/ml) at 6 Months	n	2	4	3	0,326
	Median	1242	441	419,7	
	Q1-Q3	789.0-1694	267.4-570.7	397.8-2056	
PINP (pg/ml) at 8 Months	n	2	3	3	0,574
	Median	1580	765,5	541,1	
	Q1-Q3	1004-2156	198.9-3505	381.1-1988	
PINP (pg/ml) at 10 Months	n	1	3	3	0,751
	Median	1261	652	559	

	Q1-Q3	1261-1261	447.6-3650	335.8-2567	
PINP (pg/ml) at 12 Months	n	1	1	0	
	Median	1284	701,3	.	
	Q1-Q3	1284-1284	701.3-701.3	.-.	
		**CTX(ng/ml)**			
CTX(ng/ml) at baseline	n	5	12	7	0,713
	Median	0,7	0,7	0,8	
	Q1-Q3	0.6-0.8	0.2-1.0	0.4-1.0	
CTX(ng/ml) at 2 Months	n	4	8	5	0,886
	Median	0,4	0,3	0,6	
	Q1-Q3	0.3-0.6	0.2-0.6	0.1-0.6	
CTX(ng/ml) at 4 Months	n	2	6	3	0,469
	Median	0,3	0,3	0,3	
	Q1-Q3	0.2-0.3	0.2-0.5	0.3-0.7	
CTX(ng/ml) at 6 Months	n	2	4	3	0,555
	Median	0,4	0,2	0,2	
	Q1-Q3	0.3-0.4	0.1-0.4	0.2-0.6	
CTX(ng/ml) at 8 Months	n	2	3	3	0,986
	Median	0,2	0,2	0,1	
	Q1-Q3	0.1-0.3	0.0-0.5	0.1-0.5	
CTX(ng/ml) at 10 Months	n	1	3	3	0,565
	Median	0,3	0,2	0,1	
	Q1-Q3	0.3-0.3	0.1-0.4	0.1-0.2	
CTX(ng/ml) at 12 Months	n	1	1	0	
	Median	0,2	0,4	.	
	Q1-Q3	0.2-0.2	0.4-0.4	.-.	
		**TRACP-5B (U/L)**			
TRACP-5B (U/L) at baseline	n	5	12	7	0,35
	Median	2,2	2,8	3,7	
	Q1-Q3	1.6-3.3	1.7-4.1	2.7-5.0	
TRACP-5B (U/L) at 2 Months	n	4	8	5	0,936
	Median	2	1,8	1,8	
	Q1-Q3	1.3-2.5	0.9-2.1	1.7-1.9	

TRACP-5B (U/L) at 4 Months	n	2	6	3	0,218
	Median	2,1	1,2	1,2	
	Q1-Q3	1.9-2.2	0.8-2.0	0.7-1.2	
TRACP-5B (U/L) at 6 Months	n	2	4	3	0,108
	Median	1,8	1,6	1	
	Q1-Q3	1.7-1.9	1.2-8.9	0.9-1.2	
TRACP-5B (U/L) at 8 Months	n	2	3	3	0,707
	Median	1	0,8	1	
	Q1-Q3	0.9-1.0	0.6-2.2	1.0-1.1	
TRACP-5B (U/L) at 10 Months	n	1	1	3	0,344
	Median	0,9	2	0,9	
	Q1-Q3	0.9-0.9	2.0-2.0	0.8-0.9	
TRACP-5B (U/L) at 12 Months	n	1	1	0	
	Median	0,9	1,8	.	
	Q1-Q3	0.9-0.9	1.8-1.8	.-.	
	**RANKL (pmol/L)**				
RANKL (pmol/L) at baseline	n	5	12	7	0,686
	Median	0,2	0,2	0,4	
	Q1-Q3	0.2-0.3	0.1-0.4	0.1-0.5	
RANKL (pmol/L) at 2 Months	n	4	8	5	0,659
	Median	0,2	0,2	0,2	
	Q1-Q3	0.2-0.2	0.1-0.2	0.1-0.2	
RANKL (pmol/L) at 4 Months	n	2	6	3	0,635
	Median	0,1	0,1	0,1	
	Q1-Q3	0.1-0.1	0.1-0.2	0.1-0.1	
RANKL (pmol/L) at 6 Months	n	2	4	3	0,432
	Median	0,1	0,1	0,1	
	Q1-Q3	0.1-0.1	0.1-0.2	0.1-0.1	
RANKL (pmol/L) at 8 Months	n	2	3	3	0,506
	Median	0,1	0,1	0	
	Q1-Q3	0.1-0.1	0.1-0.2	0.0-0.1	
RANKL (pmol/L) at 10 Months	n	1	3	3	0,526
	Median	0,1	0,1	0	

	Q1-Q3	0.1-0.1	0.1-0.3	0.0-0.2	
RANKL (pmol/L) at 12 Months	n	1	1	0	
	Median	0,1	0,2	.	
	Q1-Q3	0.1-0.1	0.2-0.2	.-.	
	**RANKL/OPG ratio**				
RANKL/OPG ratio at baseline	n	5	12	7	0,694
	Median	0,1	0,1	0,1	
	Q1-Q3	0.1-0.1	0.0-0.1	0.0-0.1	
RANKL/OPG ratio at 2 Months	n	4	8	5	0,692
	Median	0	0	0	
	Q1-Q3	0.0-0.1	0.0-0.1	0.0-0.0	
RANKL/OPG ratio at 4 Months	n	2	6	3	0,635
	Median	0	0	0	
	Q1-Q3	0.0-0.0	0.0-0.0	0.0-0.0	
RANKL/OPG ratio at 6 Months	n	2	4	3	0,112
	Median	0	0	0	
	Q1-Q3	0.0-0.0	0.0-0.0	0.0-0.0	
RANKL/OPG ratio at 8 Months	n	2	3	3	0,236
	Median	0	0	0	
	Q1-Q3	0.0-0.0	0.0-0.0	0.0-0.0	
RANKL/OPG ratio at 10 Months	n	1	3	3	0,368
	Median	0	0	0	
	Q1-Q3	0.0-0.0	0.0-0.1	0.0-0.0	
RANKL/OPG ratio at 12 Months	n	1	1	0	
	Median	0	0	.	
	Q1-Q3	0.0-0.0	0.0-0.0	.-.	
	**SOST (pmol/L)**				
SOST (pmol/L) at baseline	n	5	12	7	0,326
	Median	44,8	58,7	39,4	
	Q1-Q3	41.4-65.6	39.7-68.5	33.2-64.1	
SOST (pmol/L) at 2 Months	n	4	8	5	0,356
	Median	35,1	40,9	29,4	
	Q1-Q3	24.4-37.2	35.5-49.0	27.9-40.7	

SOST (pmol/L) at 4 Months	n	2	6	3	0,073
	Median	38	41,2	22,8	
	Q1-Q3	32.8-43.3	33.2-77.1	22.3-29.7	
SOST (pmol/L) at 6 Months	n	2	4	3	0,607
	Median	28,8	55,6	25,5	
	Q1-Q3	25.8-31.8	35.5-106.4	21.6-92.9	
SOST (pmol/L) at 8 Months	n	2	3	3	0,405
	Median	24,8	44,3	21,2	
	Q1-Q3	23.5-26.2	29.9-63.5	20.4-113.2	
SOST (pmol/L) at 10 Months	n	1	3	3	0,208
	Median	21,1	64,7	20,2	
	Q1-Q3	21.1-21.1	36.9-82.1	19.3-40.5	
SOST (pmol/L) at 12 Months	n	1	1	0	
	Median	20	35,7	.	
	Q1-Q3	20.0-20.0	35.7-35.7	.-.	
	**Dkk1 (pmol/L)**				
Dkk1 (pmol/L) at baseline	n	5	12	7	0,752
	Median	38	41,2	43	
	Q1-Q3	37.5-41.2	26.3-76.2	25.1-54.7	
Dkk1 (pmol/L) at 2 Months	n	4	8	5	0,649
	Median	30,6	42,2	42	
	Q1-Q3	24.2-49.7	25.7-67.3	31.4-67.5	
Dkk1 (pmol/L) at 4 Months	n	2	6	3	0,695
	Median	43,1	45,6	22,8	
	Q1-Q3	27.8-58.4	15.9-62.9	18.5-46.5	
Dkk1 (pmol/L) at 6 Months	n	2	4	3	0,449
	Median	27,6	46,1	37	
	Q1-Q3	21.2-34.0	24.7-53.6	32.0-49.2	
Dkk1 (pmol/L) at 8 Months	n	2	3	3	0,119
	Median	12,6	31	29,7	
	Q1-Q3	5.9-19.2	28.2-51.3	23.8-34.4	
Dkk1 (pmol/L) at 10 Months	n	1	3	3	0,565
	Median	9,1	26,1	31	

	Q1-Q3	9.1-9.1	16.3-28.7	8.4-38.7	
Dkk1 (pmol/L) at 12 Months	n	1	1	0	
	Median	8,4	20,4	.	
	Q1-Q3	8.4-8.4	20.4-20.4	.-.	
	**activin-A (pg/ml)**				
activin-A (pg/ml) at baseline	n	5	12	7	0,537
	Median	770,8	652	524,9	
	Q1-Q3	614.9-913.5	520.3-903.5	421.1-745.2	
activin-A (pg/ml) at 2 Months	n	4	8	5	0,445
	Median	475,3	397,5	562,2	
	Q1-Q3	454.8-503.1	300.5-507.8	390.4-627.9	
activin-A (pg/ml) at 4 Months	n	2	6	3	0,756
	Median	457,7	415,6	492,5	
	Q1-Q3	334.5-580.9	355.4-468.3	223.6-904.7	
activin-A (pg/ml) at 6 Months	n	2	4	3	0,885
	Median	402,2	380,5	378,7	
	Q1-Q3	300.0-504.5	369.7-542.1	215.0-593.8	
activin-A (pg/ml) at 8 Months	n	2	3	3	0,895
	Median	375,7	418,4	319	
	Q1-Q3	213.5-537.9	365.7-439.0	231.9-982.7	
activin-A (pg/ml) at 10 Months	n	1	3	3	0,751
	Median	362,8	357,5	283,8	
	Q1-Q3	362.8-362.8	280.5-467.5	201.0-422.7	
activin-A (pg/ml) at 12 Months	n	1	1	0	
	Median	256,8	318,2	.	
	Q1-Q3	256.8-256.8	318.2-318.2	.-.	
	**CCL3 (ng/ml)**				
CCL3 (ng/ml) at baseline	n	5	12	7	0,297
	Median	61,3	81,6	79	
	Q1-Q3	22.8-76.6	71.7-101.3	62.2-88.1	
CCL3 (ng/ml) at 2 Months	n	4	8	5	0,258
	Median	53,2	85,9	61,2	
	Q1-Q3	25.0-75.3	57.2-99.9	45.7-71.2	

CCL3 (ng/ml) at 4 Months	n	2	6	3	0,635
	Median	50,4	65,6	69,6	
	Q1-Q3	32.8-68.0	61.3-82.9	47.0-72.0	
CCL3 (ng/ml) at 6 Months	n	2	4	3	0,962
	Median	64,4	62,5	62,1	
	Q1-Q3	61.3-67.4	30.1-72.7	61.2-73.1	
CCL3 (ng/ml) at 8 Months	n	2	3	3	0,566
	Median	54,7	61,1	66,9	
	Q1-Q3	54.3-55.1	8.7-63.5	21.1-88.9	
CCL3 (ng/ml) at 10 Months	n	1	3	3	0,319
	Median	60,8	14,2	50,7	
	Q1-Q3	60.8-60.8	9.1-56.0	3.3-57.9	
CCL3 (ng/ml) at 12 Months	n	1	1	0	
	Median	64,3	3,9	.	
	Q1-Q3	64.3-64.3	3.9-3.9	.-.	

[a] Kruskal-Wallis test

Table S7. Biomarker values at each timepoint, by prior ASCT

		Prior ASCT		
		Yes	No	
		n (%)	n (%)	p-value[a]
	**bALP (µg/L)**			
bALP (µg/L) at baseline	n	14	10	0,977
	Median	11,1	10,4	
	Q1-Q3	9.3-11.5	8.9-14.0	
bALP (µg/L) at 2 Months	n	10	7	0,464
	Median	11,1	12,8	
	Q1-Q3	8.0-12.9	9.1-19.1	
bALP (µg/L) at 4 Months	n	6	5	>0.999
	Median	11,9	11,6	
	Q1-Q3	9.1-14.1	11.3-11.9	
bALP (µg/L) at 6 Months	n	6	3	0,897
	Median	13,7	11,6	
	Q1-Q3	8.1-14.8	6.8-19.7	
bALP (µg/L) at 8 Months	n	6	2	>0.999
	Median	16	11,8	
	Q1-Q3	6.8-16.9	5.6-18.0	
bALP (µg/L) at 10 Months	n	5	2	>0.999
	Median	15	12,9	
	Q1-Q3	9.8-15.8	5.9-20.0	
bALP (µg/L) at 12 Months	n	2	0	
	Median	17,1	.	
	Q1-Q3	14.5-19.7	.-.	
	**Osteocalcin (ng/ml)**			
Osteocalcin (ng/ml) at baseline	n	14	10	0,703
	Median	10	7,4	
	Q1-Q3	5.2-11.2	6.4-11.4	
Osteocalcin (ng/ml) at 2 Months	n	10	7	**0,005**
	Median	13,2	5,8	
	Q1-Q3	10.5-17.8	2.0-9.4	
Osteocalcin (ng/ml) at 4 Months	n	6	5	0,235
	Median	17,5	11,2	
	Q1-Q3	12.4-19.2	9.9-12.3	
Osteocalcin (ng/ml) at 6 Months	n	6	3	>0.999
	Median	14,5	13,9	
	Q1-Q3	10.8-25.4	11.1-18.9	
Osteocalcin (ng/ml) at 8 Months	n	6	2	0,868
	Median	16,4	12,7	
	Q1-Q3	6.0-29.8	8.5-16.9	
Osteocalcin (ng/ml) at 10 Months	n	5	2	>0.999
	Median	16,8	11,7	
	Q1-Q3	8.9-17.7	3.8-19.7	
Osteocalcin (ng/ml) at 12 Months	n	2	0	
	Median	17,1	.	
	Q1-Q3	13.3-20.8	.-.	
	**PINP (pg/ml)**			
PINP (pg/ml) at baseline	n	14	10	0,501

	Median	512,4	640,2	
	Q1-Q3	215.8-654.2	297.5-750.7	
PINP (pg/ml) at 2 Months	n	10	7	0,306
	Median	281,4	569,4	
	Q1-Q3	214.8-710.7	384.2-1020	
PINP (pg/ml) at 4 Months	n	6	5	0,411
	Median	449,4	514,2	
	Q1-Q3	278.5-777.5	490.2-601.4	
PINP (pg/ml) at 6 Months	n	6	3	>0.999
	Median	570,7	439,3	
	Q1-Q3	419.7-789.0	397.8-2056	
PINP (pg/ml) at 8 Months	n	6	2	0,868
	Median	884,9	1185	
	Q1-Q3	541.1-2156	381.1-1988	
PINP (pg/ml) at 10 Months	n	5	2	0,846
	Median	652	1452	
	Q1-Q3	559.0-1261	335.8-2567	
PINP (pg/ml) at 12 Months	n	2	0	
	Median	992,5	.	
	Q1-Q3	701.3-1284	.-.	
	**CTX(ng/ml)**			
CTX(ng/ml) at baseline	n	14	10	0,977
	Median	0,8	0,6	
	Q1-Q3	0.2-1.0	0.4-0.9	
CTX(ng/ml) at 2 Months	n	10	7	0,807
	Median	0,4	0,4	
	Q1-Q3	0.1-0.7	0.2-0.6	
CTX(ng/ml) at 4 Months	n	6	5	0,523
	Median	0,4	0,3	
	Q1-Q3	0.2-0.7	0.2-0.3	
CTX(ng/ml) at 6 Months	n	6	3	0,519
	Median	0,4	0,2	
	Q1-Q3	0.1-0.6	0.2-0.2	
CTX(ng/ml) at 8 Months	n	6	2	0,405
	Median	0,2	0,1	
	Q1-Q3	0.1-0.5	0.1-0.1	
CTX(ng/ml) at 10 Months	n	5	2	0,333
	Median	0,2	0,1	
	Q1-Q3	0.2-0.3	0.1-0.1	
CTX(ng/ml) at 12 Months	n	2	0	
	Median	0,3	.	
	Q1-Q3	0.2-0.4	.-.	
	**TRACP-5B (U/L)**			
TRACP-5B (U/L) at baseline	n	14	10	0,703
	Median	3,4	3,2	
	Q1-Q3	1.9-3.7	1.5-5.0	
TRACP-5B (U/L) at 2 Months	n	10	7	0,262
	Median	1,7	1,9	
	Q1-Q3	0.8-2.0	1.8-2.4	
TRACP-5B (U/L) at 4 Months	n	6	5	0,784

	Median	1,1	1,3	
	Q1-Q3	0.8-2.0	1.2-1.9	
TRACP-5B (U/L) at 6 Months	n	6	3	0,093
	Median	1,8	1	
	Q1-Q3	1.2-1.9	0.9-1.3	
TRACP-5B (U/L) at 8 Months	n	6	2	0,405
	Median	1	1	
	Q1-Q3	0.8-1.0	1.0-1.1	
TRACP-5B (U/L) at 10 Months	n	3	2	0,386
	Median	0,9	0,8	
	Q1-Q3	0.9-2.0	0.8-0.9	
TRACP-5B (U/L) at 12 Months	n	2	0	
	Median	1,3	.	
	Q1-Q3	0.9-1.8	.-.	
	**RANKL (pmol/L)**			
RANKL (pmol/L) at baseline	n	14	10	0,619
	Median	0,3	0,3	
	Q1-Q3	0.2-0.4	0.1-0.4	
RANKL (pmol/L) at 2 Months	n	10	7	0,884
	Median	0,2	0,2	
	Q1-Q3	0.1-0.2	0.1-0.2	
RANKL (pmol/L) at 4 Months	n	6	5	0,927
	Median	0,1	0,1	
	Q1-Q3	0.1-0.2	0.1-0.1	
RANKL (pmol/L) at 6 Months	n	6	3	0,699
	Median	0,1	0,1	
	Q1-Q3	0.1-0.2	0.1-0.1	
RANKL (pmol/L) at 8 Months	n	6	2	0,868
	Median	0,1	0,1	
	Q1-Q3	0.1-0.1	0.0-0.1	
RANKL (pmol/L) at 10 Months	n	5	2	>0.999
	Median	0,1	0,1	
	Q1-Q3	0.1-0.1	0.0-0.2	
RANKL (pmol/L) at 12 Months	n	2	0	
	Median	0,1	.	
	Q1-Q3	0.1-0.2	.-.	
	**RANKL/OPG ratio**			
RANKL/OPG ratio at baseline	n	14	10	0,464
	Median	0,1	0,1	
	Q1-Q3	0.0-0.1	0.0-0.1	
RANKL/OPG ratio at 2 Months	n	10	7	0,526
	Median	0	0	
	Q1-Q3	0.0-0.0	0.0-0.0	
RANKL/OPG ratio at 4 Months	n	6	5	0,784
	Median	0	0	
	Q1-Q3	0.0-0.0	0.0-0.0	
RANKL/OPG ratio at 6 Months	n	6	3	0,366
	Median	0	0	
	Q1-Q3	0.0-0.0	0.0-0.0	
RANKL/OPG ratio at 8 Months	n	6	2	0,617

	Median	0	0	
	Q1-Q3	0.0-0.0	0.0-0.0	
RANKL/OPG ratio at 10 Months	n	5	2	>0.999
	Median	0	0	
	Q1-Q3	0.0-0.0	0.0-0.0	
RANKL/OPG ratio at 12 Months	n	2	0	
	Median	0	.	
	Q1-Q3	0.0-0.0	.-.	
	**SOST (pmol/L)**			
SOST (pmol/L) at baseline	n	14	10	0,93
	Median	43,3	53,3	
	Q1-Q3	37.9-66.3	38.1-64.1	
SOST (pmol/L) at 2 Months	n	10	7	0,088
	Median	38,6	27,9	
	Q1-Q3	34.0-49.7	21.5-40.7	
SOST (pmol/L) at 4 Months	n	6	5	0,784
	Median	35	29,7	
	Q1-Q3	32.8-45.8	25.0-43.3	
SOST (pmol/L) at 6 Months	n	6	3	0,519
	Median	28,8	63,1	
	Q1-Q3	22.9-48.1	25.5-92.9	
SOST (pmol/L) at 8 Months	n	6	2	0,868
	Median	28	67,2	
	Q1-Q3	23.5-44.3	21.2-113.2	
SOST (pmol/L) at 10 Months	n	5	2	0,846
	Median	36,9	30,4	
	Q1-Q3	21.1-64.7	20.2-40.5	
SOST (pmol/L) at 12 Months	n	2	0	
	Median	27,8	.	
	Q1-Q3	20.0-35.7	.-.	
	**Dkk1 (pmol/L)**			
Dkk1 (pmol/L) at baseline	n	14	10	0,86
	Median	40	42,1	
	Q1-Q3	31.4-52.4	25.1-75.9	
Dkk1 (pmol/L) at 2 Months	n	10	7	0,661
	Median	34,1	42	
	Q1-Q3	26.9-58.4	24.4-72.2	
Dkk1 (pmol/L) at 4 Months	n	6	5	0,927
	Median	30,7	46,5	
	Q1-Q3	22.8-57.5	18.5-58.4	
Dkk1 (pmol/L) at 6 Months	n	6	3	0,699
	Median	36,8	37	
	Q1-Q3	32.0-52.4	9.7-49.2	
Dkk1 (pmol/L) at 8 Months	n	6	2	0,868
	Median	29,6	26,8	
	Q1-Q3	19.2-34.4	23.8-29.7	
Dkk1 (pmol/L) at 10 Months	n	5	2	>0.999
	Median	26,1	23,6	
	Q1-Q3	16.3-28.7	8.4-38.7	
Dkk1 (pmol/L) at 12 Months	n	2	0	

	Median	14,4	.	
	Q1-Q3	8.4-20.4	.-.	
	**activin-A (pg/ml)**			
activin-A (pg/ml) at baseline	n	14	10	0,501
	Median	681,1	551,2	
	Q1-Q3	492.3-904.2	505.0-770.8	
activin-A (pg/ml) at 2 Months	n	10	7	0,961
	Median	454,8	488,4	
	Q1-Q3	358.2-562.2	315.5-538.3	
activin-A (pg/ml) at 4 Months	n	6	5	0,648
	Median	415,6	519,6	
	Q1-Q3	355.4-468.3	223.6-580.9	
activin-A (pg/ml) at 6 Months	n	6	3	0,699
	Median	383,5	372,6	
	Q1-Q3	366.9-504.5	215.0-593.8	
activin-A (pg/ml) at 8 Months	n	6	2	0,617
	Median	392	650,8	
	Q1-Q3	231.9-439.0	319.0-982.7	
activin-A (pg/ml) at 10 Months	n	5	2	0,846
	Median	357,5	311,8	
	Q1-Q3	283.8-362.8	201.0-422.7	
activin-A (pg/ml) at 12 Months	n	2	0	
	Median	287,5	.	
	Q1-Q3	256.8-318.2	.-.	
	**CCL3 (ng/ml)**			
CCL3 (ng/ml) at baseline	n	14	10	**0,024**
	Median	85	61,8	
	Q1-Q3	75.3-98.9	22.8-79.0	
CCL3 (ng/ml) at 2 Months	n	10	7	**0,017**
	Median	86,3	39	
	Q1-Q3	67.4-97.0	10.4-71.2	
CCL3 (ng/ml) at 4 Months	n	6	5	0,648
	Median	68	61,3	
	Q1-Q3	63.3-72.0	47.0-69.6	
CCL3 (ng/ml) at 6 Months	n	6	3	0,699
	Median	64,4	62,1	
	Q1-Q3	54.0-71.1	61.2-74.3	
CCL3 (ng/ml) at 8 Months	n	6	2	>0.999
	Median	58,1	44	
	Q1-Q3	54.3-63.5	21.1-66.9	
CCL3 (ng/ml) at 10 Months	n	5	2	0,846
	Median	50,7	30,6	
	Q1-Q3	14.2-56.0	3.3-57.9	
CCL3 (ng/ml) at 12 Months	n	2	0	
	Median	34,1	.	
	Q1-Q3	3.9-64.3	.-.	

[a] Mann-Whitney U test

Table S8. Biomarker values at each timepoint, by prior radiotherapy

		Prior radiotherapy		
		Yes	No	
		n (%)	n (%)	p-value[a]
	bALP (µg/L)			
bALP (µg/L) at baseline	n	6	18	0,334
	Median	11,2	10,8	
	Q1-Q3	10.0-14.0	8.9-11.5	
bALP (µg/L) at 2 Months	n	4	13	0,193
	Median	14,1	10,1	
	Q1-Q3	12.4-17.3	8.0-12.9	
bALP (µg/L) at 4 Months	n	1	10	0,874
	Median	11,3	11,7	
	Q1-Q3	11.3-11.3	9.1-14.1	
bALP (µg/L) at 6 Months	n	1	8	>0.999
	Median	13,6	12,7	
	Q1-Q3	13.6-13.6	7.6-14.8	
bALP (µg/L) at 8 Months	n	1	7	>0.999
	Median	15	16,9	
	Q1-Q3	15.0-15.0	5.6-18.0	
bALP (µg/L) at 10 Months	n	1	6	>0.999
	Median	15	12,8	
	Q1-Q3	15.0-15.0	7.0-18.1	
bALP (µg/L) at 12 Months	n	1	1	
	Median	14,5	19,7	
	Q1-Q3	14.5-14.5	19.7-19.7	
	Osteocalcin (ng/ml)			
Osteocalcin (ng/ml) at baseline	n	6	18	0,571
	Median	8,9	9,2	
	Q1-Q3	5.8-11.9	5.2-10.8	
Osteocalcin (ng/ml) at 2 Months	n	4	13	0,336
	Median	7,6	10,9	
	Q1-Q3	4.6-12.2	9.4-14.1	
Osteocalcin (ng/ml) at 4 Months	n	1	10	0,155
	Median	1	14,8	
	Q1-Q3	1.0-1.0	11.2-19.2	
Osteocalcin (ng/ml) at 6 Months	n	1	8	0,846
	Median	16,2	13,4	
	Q1-Q3	16.2-16.2	10.9-22.2	
Osteocalcin (ng/ml) at 8 Months	n	1	7	0,663
	Median	17,8	14,9	
	Q1-Q3	17.8-17.8	6.0-29.8	
Osteocalcin (ng/ml) at 10 Months	n	1	6	0,803
	Median	17,7	12,8	
	Q1-Q3	17.7-17.7	3.8-19.7	
Osteocalcin (ng/ml) at 12 Months	n	1	1	
	Median	20,8	13,3	
	Q1-Q3	20.8-20.8	13.3-13.3	
	PINP (pg/ml)			
PINP (pg/ml) at baseline	n	6	18	0,334

	Median	698,1	477,8	
	Q1-Q3	547.4-780.4	292.1-733.1	
PINP (pg/ml) at 2 Months	n	4	13	0,61
	Median	674,5	384,2	
	Q1-Q3	352.2-900.0	226.3-710.7	
PINP (pg/ml) at 4 Months	n	1	10	0,874
	Median	514,2	474,8	
	Q1-Q3	514.2-514.2	411.6-777.5	
PINP (pg/ml) at 6 Months	n	1	8	0,561
	Median	789	441	
	Q1-Q3	789.0-789.0	408.7-1196	
PINP (pg/ml) at 8 Months	n	1	7	>0.999
	Median	1004	765,5	
	Q1-Q3	1004-1004	381.1-2156	
PINP (pg/ml) at 10 Months	n	1	6	0,803
	Median	1261	605,5	
	Q1-Q3	1261-1261	447.6-2567	
PINP (pg/ml) at 12 Months	n	1	1	
	Median	1284	701,3	
	Q1-Q3	1284-1284	701.3-701.3	
	**CTX(ng/ml)**			
CTX(ng/ml) at baseline	n	6	18	0,665
	Median	0,7	0,7	
	Q1-Q3	0.7-0.8	0.2-1.0	
CTX(ng/ml) at 2 Months	n	4	13	0,91
	Median	0,4	0,4	
	Q1-Q3	0.2-0.6	0.2-0.6	
CTX(ng/ml) at 4 Months	n	1	10	0,635
	Median	0,2	0,3	
	Q1-Q3	0.2-0.2	0.2-0.5	
CTX(ng/ml) at 6 Months	n	1	8	0,561
	Median	0,4	0,2	
	Q1-Q3	0.4-0.4	0.2-0.5	
CTX(ng/ml) at 8 Months	n	1	7	0,663
	Median	0,3	0,1	
	Q1-Q3	0.3-0.3	0.1-0.5	
CTX(ng/ml) at 10 Months	n	1	6	0,453
	Median	0,3	0,2	
	Q1-Q3	0.3-0.3	0.1-0.2	
CTX(ng/ml) at 12 Months	n	1	1	
	Median	0,2	0,4	
	Q1-Q3	0.2-0.2	0.4-0.4	
	**TRACP-5B (U/L)**			
TRACP-5B (U/L) at baseline	n	6	18	0,443
	Median	3,7	3	
	Q1-Q3	2.2-3.7	1.5-4.0	
TRACP-5B (U/L) at 2 Months	n	4	13	0,955
	Median	1,9	1,8	
	Q1-Q3	1.3-2.0	1.0-2.1	
TRACP-5B (U/L) at 4 Months	n	1	10	0,635

	Median	1,9	1,2	
	Q1-Q3	1.9-1.9	0.8-2.0	
TRACP-5B (U/L) at 6 Months	n	1	8	0,846
	Median	1,7	1,2	
	Q1-Q3	1.7-1.7	1.0-1.9	
TRACP-5B (U/L) at 8 Months	n	1	7	0,663
	Median	0,9	1	
	Q1-Q3	0.9-0.9	0.8-1.1	
TRACP-5B (U/L) at 10 Months	n	1	4	0,724
	Median	0,9	0,9	
	Q1-Q3	0.9-0.9	0.8-1.4	
TRACP-5B (U/L) at 12 Months	n	1	1	
	Median	0,9	1,8	
	Q1-Q3	0.9-0.9	1.8-1.8	
	**RANKL (pmol/L)**			
RANKL (pmol/L) at baseline	n	6	18	0,062
	Median	0,4	0,2	
	Q1-Q3	0.2-0.5	0.1-0.4	
RANKL (pmol/L) at 2 Months	n	4	13	0,955
	Median	0,2	0,2	
	Q1-Q3	0.1-0.2	0.1-0.2	
RANKL (pmol/L) at 4 Months	n	1	10	0,429
	Median	0,1	0,1	
	Q1-Q3	0.1-0.1	0.1-0.1	
RANKL (pmol/L) at 6 Months	n	1	8	>0.999
	Median	0,1	0,1	
	Q1-Q3	0.1-0.1	0.1-0.1	
RANKL (pmol/L) at 8 Months	n	1	7	>0.999
	Median	0,1	0,1	
	Q1-Q3	0.1-0.1	0.0-0.1	
RANKL (pmol/L) at 10 Months	n	1	6	>0.999
	Median	0,1	0,1	
	Q1-Q3	0.1-0.1	0.0-0.2	
RANKL (pmol/L) at 12 Months	n	1	1	
	Median	0,1	0,2	
	Q1-Q3	0.1-0.1	0.2-0.2	
	**RANKL/OPG ratio**			
RANKL/OPG ratio at baseline	n	6	18	0,117
	Median	0,1	0,1	
	Q1-Q3	0.1-0.1	0.0-0.1	
RANKL/OPG ratio at 2 Months	n	4	13	0,61
	Median	0	0	
	Q1-Q3	0.0-0.0	0.0-0.0	
RANKL/OPG ratio at 4 Months	n	1	10	0,635
	Median	0	0	
	Q1-Q3	0.0-0.0	0.0-0.0	
RANKL/OPG ratio at 6 Months	n	1	8	0,561
	Median	0	0	
	Q1-Q3	0.0-0.0	0.0-0.0	
RANKL/OPG ratio at 8 Months	n	1	7	0,663

	Median	0	0	
	Q1-Q3	0.0-0.0	0.0-0.0	
RANKL/OPG ratio at 10 Months	n	1	6	0,803
	Median	0	0	
	Q1-Q3	0.0-0.0	0.0-0.0	
RANKL/OPG ratio at 12 Months	n	1	1	
	Median	0	0	
	Q1-Q3	0.0-0.0	0.0-0.0	
	**SOST (pmol/L)**			
SOST (pmol/L) at baseline	n	6	18	0,571
	Median	54,5	46	
	Q1-Q3	41.4-64.6	37.7-66.3	
SOST (pmol/L) at 2 Months	n	4	13	0,955
	Median	36,1	37,2	
	Q1-Q3	28.0-61.8	29.4-41.7	
SOST (pmol/L) at 4 Months	n	1	10	0,635
	Median	43,3	33	
	Q1-Q3	43.3-43.3	25.0-45.8	
SOST (pmol/L) at 6 Months	n	1	8	>0.999
	Median	31,8	37	
	Q1-Q3	31.8-31.8	24.2-78.0	
SOST (pmol/L) at 8 Months	n	1	7	0,663
	Median	23,5	29,9	
	Q1-Q3	23.5-23.5	21.2-63.5	
SOST (pmol/L) at 10 Months	n	1	6	0,803
	Median	21,1	38,7	
	Q1-Q3	21.1-21.1	20.2-64.7	
SOST (pmol/L) at 12 Months	n	1	1	
	Median	20	35,7	
	Q1-Q3	20.0-20.0	35.7-35.7	
	**Dkk1 (pmol/L)**			
Dkk1 (pmol/L) at baseline	n	6	18	>0.999
	Median	42,1	40	
	Q1-Q3	37.5-50.9	21.2-75.9	
Dkk1 (pmol/L) at 2 Months	n	4	13	0,955
	Median	44,7	36,9	
	Q1-Q3	25.6-67.3	28.7-58.4	
Dkk1 (pmol/L) at 4 Months	n	1	10	0,429
	Median	58,4	30,7	
	Q1-Q3	58.4-58.4	18.5-57.5	
Dkk1 (pmol/L) at 6 Months	n	1	8	0,333
	Median	21,2	38,3	
	Q1-Q3	21.2-21.2	33.0-50.8	
Dkk1 (pmol/L) at 8 Months	n	1	7	0,383
	Median	19,2	29,7	
	Q1-Q3	19.2-19.2	23.8-34.4	
Dkk1 (pmol/L) at 10 Months	n	1	6	0,453
	Median	9,1	27,4	
	Q1-Q3	9.1-9.1	16.3-31.0	
Dkk1 (pmol/L) at 12 Months	n	1	1	

	Median	8,4	20,4	
	Q1-Q3	8.4-8.4	20.4-20.4	
	**activin-A (pg/ml)**			
activin-A (pg/ml) at baseline	n	6	18	0,868
	Median	692,8	652	
	Q1-Q3	421.1-921.2	505.0-902.7	
activin-A (pg/ml) at 2 Months	n	4	13	0,126
	Median	540	436,9	
	Q1-Q3	482.6-674.5	321.0-488.4	
activin-A (pg/ml) at 4 Months	n	1	10	0,268
	Median	580,9	415,6	
	Q1-Q3	580.9-580.9	334.5-492.5	
activin-A (pg/ml) at 6 Months	n	1	8	0,333
	Median	300	383,5	
	Q1-Q3	300.0-300.0	369.7-549.1	
activin-A (pg/ml) at 8 Months	n	1	7	0,19
	Median	213,5	418,4	
	Q1-Q3	213.5-213.5	319.0-537.9	
activin-A (pg/ml) at 10 Months	n	1	6	0,803
	Median	362,8	320,7	
	Q1-Q3	362.8-362.8	280.5-422.7	
activin-A (pg/ml) at 12 Months	n	1	1	
	Median	256,8	318,2	
	Q1-Q3	256.8-256.8	318.2-318.2	
	**CCL3 (ng/ml)**			
CCL3 (ng/ml) at baseline	n	6	18	0,102
	Median	49,7	80,8	
	Q1-Q3	3.1-79.0	70.9-98.9	
CCL3 (ng/ml) at 2 Months	n	4	13	0,126
	Median	28,4	71,2	
	Q1-Q3	7.5-64.4	61.2-95.5	
CCL3 (ng/ml) at 4 Months	n	1	10	0,155
	Median	32,8	68	
	Q1-Q3	32.8-32.8	61.3-72.0	
CCL3 (ng/ml) at 6 Months	n	1	8	0,846
	Median	67,4	61,7	
	Q1-Q3	67.4-67.4	57.6-72.1	
CCL3 (ng/ml) at 8 Months	n	1	7	0,663
	Median	54,3	61,1	
	Q1-Q3	54.3-54.3	21.1-66.9	
CCL3 (ng/ml) at 10 Months	n	1	6	0,211
	Median	60,8	32,4	
	Q1-Q3	60.8-60.8	9.1-56.0	
CCL3 (ng/ml) at 12 Months	n	1	1	
	Median	64,3	3,9	
	Q1-Q3	64.3-64.3	3.9-3.9	

[a] Mann-Whitney U test

Table S9. Biomarker values at each timepoint, by refractoriness to PI

		Refractorinesss to PI		
		**Yes**	**No**	
		**n (%)**	**n (%)**	**p-value[a]**
	**bALP (µg/L)**			
bALP (µg/L) at baseline	n	10	14	0,429
	Median	11,5	10,6	
	Q25-Q75	9.3-11.7	8.9-11.3	
bALP (µg/L) at 2 Months	n	8	9	0,81
	Median	12,4	11,7	
	Q25-Q75	8.6-18.3	9.1-12.9	
bALP (µg/L) at 4 Months	n	5	6	0,121
	Median	10,8	13,5	
	Q25-Q75	9.1-11.3	11.6-14.4	
bALP (µg/L) at 6 Months	n	5	4	0,27
	Median	11,6	14,3	
	Q25-Q75	8.1-13.6	10.4-17.3	
bALP (µg/L) at 8 Months	n	4	4	0,112
	Median	10,3	17,4	
	Q25-Q75	4.7-16.0	11.9-18.8	
bALP (µg/L) at 10 Months	n	4	3	0,216
	Median	11	18,1	
	Q25-Q75	6.4-15.4	9.8-20.0	
bALP (µg/L) at 12 Months	n	2	0	
	Median	17,1	.	
	Q25-Q75	14.5-19.7	.-.	
	**Osteocalcin (ng/ml)**			
Osteocalcin (ng/ml) at baseline	n	10	14	0,279
	Median	7,9	9,5	
	Q25-Q75	3.9-10.8	5.8-11.9	
Osteocalcin (ng/ml) at 2 Months	n	8	9	0,163
	Median	8,1	12,2	
	Q25-Q75	2.8-12.9	9.4-14.1	
Osteocalcin (ng/ml) at 4 Months	n	5	6	0,648
	Median	12,4	15	
	Q25-Q75	9.9-17.2	11.2-19.2	
Osteocalcin (ng/ml) at 6 Months	n	5	4	0,391
	Median	12,8	19,7	
	Q25-Q75	11.1-16.2	12.4-26.1	
Osteocalcin (ng/ml) at 8 Months	n	4	4	0,47
	Median	11,7	23,3	
	Q25-Q75	7.3-16.4	11.2-34.9	
Osteocalcin (ng/ml) at 10 Months	n	4	3	0,216
	Median	10,3	19,7	
	Q25-Q75	3.3-17.2	8.9-28.2	
Osteocalcin (ng/ml) at 12 Months	n	2	0	
	Median	17,1	.	
	Q25-Q75	13.3-20.8	.-.	
	**PINP (pg/ml)**			
PINP (pg/ml) at baseline	n	10	14	0,747

	Median	560,1	477,8	
	Q25-Q75	310.5-654.2	253.1-750.7	
PINP (pg/ml) at 2 Months	n	8	9	0,885
	Median	640	384,2	
	Q25-Q75	196.9-777.4	229.5-550.8	
PINP (pg/ml) at 4 Months	n	5	6	0,523
	Median	490,2	530,4	
	Q25-Q75	411.6-514.2	439.2-1567	
PINP (pg/ml) at 6 Months	n	5	4	0,27
	Median	439,3	1068	
	Q25-Q75	397.8-698.7	431.2-1875	
PINP (pg/ml) at 8 Months	n	4	4	0,112
	Median	573,3	2072	
	Q25-Q75	290.0-884.9	1265-2831	
PINP (pg/ml) at 10 Months	n	4	3	0,216
	Median	549,8	2567	
	Q25-Q75	391.7-956.6	559.0-3650	
PINP (pg/ml) at 12 Months	n	2	0	
	Median	992,5	.	
	Q25-Q75	701.3-1284	.-.	
	**CTX(ng/ml)**			
CTX(ng/ml) at baseline	n	10	14	0,703
	Median	0,6	0,8	
	Q25-Q75	0.2-0.9	0.3-1.0	
CTX(ng/ml) at 2 Months	n	8	9	0,386
	Median	0,4	0,5	
	Q25-Q75	0.1-0.6	0.2-0.6	
CTX(ng/ml) at 4 Months	n	5	6	0,235
	Median	0,2	0,3	
	Q25-Q75	0.2-0.3	0.3-0.5	
CTX(ng/ml) at 6 Months	n	5	4	0,903
	Median	0,2	0,2	
	Q25-Q75	0.2-0.4	0.2-0.5	
CTX(ng/ml) at 8 Months	n	4	4	0,665
	Median	0,2	0,1	
	Q25-Q75	0.1-0.4	0.1-0.3	
CTX(ng/ml) at 10 Months	n	4	3	0,377
	Median	0,3	0,1	
	Q25-Q75	0.1-0.4	0.1-0.2	
CTX(ng/ml) at 12 Months	n	2	0	
	Median	0,3	.	
	Q25-Q75	0.2-0.4	.-.	
	**TRACP-5B (U/L)**			
TRACP-5B (U/L) at baseline	n	10	14	0,792
	Median	2,4	3,6	
	Q25-Q75	1.8-3.7	1.3-4.0	
TRACP-5B (U/L) at 2 Months	n	8	9	0,665
	Median	1,9	1,7	
	Q25-Q75	1.8-2.0	0.8-2.4	
TRACP-5B (U/L) at 4 Months	n	5	6	0,927

	Median	1,3	1,2	
	Q25-Q75	0.8-1.9	1.0-2.1	
TRACP-5B (U/L) at 6 Months	n	5	4	0,54
	Median	1,7	1,1	
	Q25-Q75	1.3-1.9	1.0-1.5	
TRACP-5B (U/L) at 8 Months	n	4	4	0,885
	Median	1	1	
	Q25-Q75	0.8-1.6	0.9-1.0	
TRACP-5B (U/L) at 10 Months	n	3	2	0,773
	Median	0,9	0,9	
	Q25-Q75	0.8-2.0	0.9-0.9	
TRACP-5B (U/L) at 12 Months	n	2	0	
	Median	1,3	.	
	Q25-Q75	0.9-1.8	.-.	
	**RANKL (pmol/L)**			
RANKL (pmol/L) at baseline	n	10	14	0,815
	Median	0,2	0,3	
	Q25-Q75	0.1-0.5	0.2-0.4	
RANKL (pmol/L) at 2 Months	n	8	9	0,962
	Median	0,2	0,2	
	Q25-Q75	0.1-0.2	0.2-0.2	
RANKL (pmol/L) at 4 Months	n	5	6	0,121
	Median	0,1	0,1	
	Q25-Q75	0.1-0.1	0.1-0.1	
RANKL (pmol/L) at 6 Months	n	5	4	0,903
	Median	0,1	0,1	
	Q25-Q75	0.1-0.1	0.1-0.1	
RANKL (pmol/L) at 8 Months	n	4	4	0,061
	Median	0,1	0,1	
	Q25-Q75	0.1-0.2	0.0-0.1	
RANKL (pmol/L) at 10 Months	n	4	3	0,052
	Median	0,1	0	
	Q25-Q75	0.1-0.2	0.0-0.1	
RANKL (pmol/L) at 12 Months	n	2	0	
	Median	0,1	.	
	Q25-Q75	0.1-0.2	.-.	
	**RANKL/OPG ratio**			
RANKL/OPG ratio at baseline	n	10	14	0,792
	Median	0,1	0,1	
	Q25-Q75	0.0-0.1	0.0-0.1	
RANKL/OPG ratio at 2 Months	n	8	9	0,962
	Median	0	0	
	Q25-Q75	0.0-0.1	0.0-0.0	
RANKL/OPG ratio at 4 Months	n	5	6	0,121
	Median	0	0	
	Q25-Q75	0.0-0.0	0.0-0.0	
RANKL/OPG ratio at 6 Months	n	5	4	0,713
	Median	0	0	
	Q25-Q75	0.0-0.0	0.0-0.0	
RANKL/OPG ratio at 8 Months	n	4	4	0,112

	Median	0	0	
	Q25-Q75	0.0-0.0	0.0-0.0	
RANKL/OPG ratio at 10 Months	n	4	3	0,216
	Median	0	0	
	Q25-Q75	0.0-0.0	0.0-0.0	
RANKL/OPG ratio at 12 Months	n	2	0	
	Median	0	.	
	Q25-Q75	0.0-0.0	.-.	
	**SOST (pmol/L)**			
SOST (pmol/L) at baseline	n	10	14	0,578
	Median	47,6	48,8	
	Q25-Q75	39.4-66.3	37.7-64.1	
SOST (pmol/L) at 2 Months	n	8	9	0,665
	Median	36,1	37,2	
	Q25-Q75	27.9-40.4	29.4-48.3	
SOST (pmol/L) at 4 Months	n	5	6	0,171
	Median	43,3	28,9	
	Q25-Q75	33.2-45.8	22.8-36.7	
SOST (pmol/L) at 6 Months	n	5	4	0,54
	Median	48,1	25,6	
	Q25-Q75	31.8-63.1	23.5-87.7	
SOST (pmol/L) at 8 Months	n	4	4	0,312
	Median	37,1	23,7	
	Q25-Q75	26.7-78.7	20.8-44.8	
SOST (pmol/L) at 10 Months	n	4	3	0,596
	Median	38,7	20,2	
	Q25-Q75	29.0-52.6	19.3-82.1	
SOST (pmol/L) at 12 Months	n	2	0	
	Median	27,8	.	
	Q25-Q75	20.0-35.7	.-.	
	**Dkk1 (pmol/L)**			
Dkk1 (pmol/L) at baseline	n	10	14	0,229
	Median	47,7	37,7	
	Q25-Q75	32.0-76.6	25.1-50.9	
Dkk1 (pmol/L) at 2 Months	n	8	9	0,413
	Median	58,9	31,4	
	Q25-Q75	26.5-69.8	26.9-42.0	
Dkk1 (pmol/L) at 4 Months	n	5	6	0,648
	Median	57,5	30,7	
	Q25-Q75	18.5-58.4	22.8-46.5	
Dkk1 (pmol/L) at 6 Months	n	5	4	>0.999
	Median	37	36,8	
	Q25-Q75	21.2-52.4	33.0-44.4	
Dkk1 (pmol/L) at 8 Months	n	4	4	0,885
	Median	26	30,4	
	Q25-Q75	21.5-39.7	17.8-32.7	
Dkk1 (pmol/L) at 10 Months	n	4	3	0,216
	Median	17,6	31	
	Q25-Q75	8.8-27.4	16.3-38.7	
Dkk1 (pmol/L) at 12 Months	n	2	0	

	Median	14,4	.	
	Q25-Q75	8.4-20.4	.-.	
	**activin-A (pg/ml)**			
activin-A (pg/ml) at baseline	n	10	14	0,429
	Median	719,4	595,1	
	Q25-Q75	505.0-904.2	421.1-745.2	
activin-A (pg/ml) at 2 Months	n	8	9	0,136
	Median	413,6	488,4	
	Q25-Q75	300.5-482.6	462.2-562.2	
activin-A (pg/ml) at 4 Months	n	5	6	0,055
	Median	519,6	344,9	
	Q25-Q75	418.7-580.9	223.6-468.3	
activin-A (pg/ml) at 6 Months	n	5	4	0,391
	Median	388,3	372,8	
	Q25-Q75	372.6-593.8	290.9-441.6	
activin-A (pg/ml) at 8 Months	n	4	4	0,885
	Median	402,3	368,7	
	Q25-Q75	289.6-710.9	275.4-478.2	
activin-A (pg/ml) at 10 Months	n	4	3	0,86
	Median	360,2	283,8	
	Q25-Q75	319.0-392.7	201.0-467.5	
activin-A (pg/ml) at 12 Months	n	2	0	
	Median	287,5	.	
	Q25-Q75	256.8-318.2	.-.	
	**CCL3 (ng/ml)**			
CCL3 (ng/ml) at baseline	n	10	14	0,501
	Median	73,9	80,8	
	Q25-Q75	22.8-94.8	70.9-88.5	
CCL3 (ng/ml) at 2 Months	n	8	9	0,312
	Median	52,6	71,2	
	Q25-Q75	10.7-90.1	67.4-89.4	
CCL3 (ng/ml) at 4 Months	n	5	6	0,648
	Median	47	68	
	Q25-Q75	34.3-82.9	63.3-69.6	
CCL3 (ng/ml) at 6 Months	n	5	4	0,903
	Median	67,4	61,7	
	Q25-Q75	61.2-71.1	57.6-67.6	
CCL3 (ng/ml) at 8 Months	n	4	4	0,112
	Median	37,7	64	
	Q25-Q75	14.9-58.9	58.1-77.9	
CCL3 (ng/ml) at 10 Months	n	4	3	0,86
	Median	32,5	50,7	
	Q25-Q75	6.2-58.4	14.2-57.9	
CCL3 (ng/ml) at 12 Months	n	2	0	
	Median	34,1	.	
	Q25-Q75	3.9-64.3	.-.	

[a] Mann-Whitney U test

Table S10. Biomarker values at each timepoint, by refractoriness to IMiD

		Refractorinesss to IMiD		
		**Yes**	**No**	
		**n (%)**	**n (%)**	**p-value**[a]
	**bALP (µg/L)**			
bALP (µg/L) at baseline	n	16	8	0,105
	Median	11,3	9,5	
	Q1-Q3	10.4-11.7	8.1-11.0	
bALP (µg/L) at 2 Months	n	11	6	0,079
	Median	12,9	9,6	
	Q1-Q3	9.4-19.1	8.0-11.7	
bALP (µg/L) at 4 Months	n	7	4	0,925
	Median	11,3	12,2	
	Q1-Q3	9.1-14.1	9.6-13.7	
bALP (µg/L) at 6 Months	n	7	2	0,188
	Median	11,6	16,7	
	Q1-Q3	7.1-14.8	13.7-19.7	
bALP (µg/L) at 8 Months	n	6	2	0,243
	Median	10,9	17,4	
	Q1-Q3	5.6-16.9	16.9-18.0	
bALP (µg/L) at 10 Months	n	5	2	0,081
	Median	9,8	19	
	Q1-Q3	7.0-15.0	18.1-20.0	
bALP (µg/L) at 12 Months	n	2	0	
	Median	17,1	.	
	Q1-Q3	14.5-19.7	.-.	
	**Osteocalcin (ng/ml)**			
Osteocalcin (ng/ml) at baseline	n	16	8	0,092
	Median	7,9	11,6	
	Q1-Q3	4.6-10.4	7.4-14.7	
Osteocalcin (ng/ml) at 2 Months	n	11	6	0,393
	Median	10,5	12,3	
	Q1-Q3	3.5-14.9	9.4-14.1	
Osteocalcin (ng/ml) at 4 Months	n	7	4	0,777
	Median	17,2	11,7	
	Q1-Q3	9.9-19.2	9.9-16.0	
Osteocalcin (ng/ml) at 6 Months	n	7	2	0,464
	Median	12,8	19,7	
	Q1-Q3	10.8-18.9	13.9-25.4	
Osteocalcin (ng/ml) at 8 Months	n	6	2	0,405
	Median	11,7	23,3	
	Q1-Q3	6.0-17.8	16.9-29.8	
Osteocalcin (ng/ml) at 10 Months	n	5	2	0,081
	Median	8,9	23,9	
	Q1-Q3	3.8-16.8	19.7-28.2	
Osteocalcin (ng/ml) at 12 Months	n	2	0	
	Median	17,1	.	
	Q1-Q3	13.3-20.8	.-.	
	**PINP (pg/ml)**			
PINP (pg/ml) at baseline	n	16	8	0,343

	Median	560,1	415,2	
	Q1-Q3	304.0-781.0	254.0-737.6	
PINP (pg/ml) at 2 Months	n	11	6	0,88
	Median	569,4	384,6	
	Q1-Q3	178.9-779.6	333.2-550.8	
PINP (pg/ml) at 4 Months	n	7	4	0,508
	Median	490,2	530,4	
	Q1-Q3	278.5-777.5	449.4-1249	
PINP (pg/ml) at 6 Months	n	7	2	0,661
	Median	442,8	1238	
	Q1-Q3	397.8-789.0	419.7-2056	
PINP (pg/ml) at 8 Months	n	6	2	>0.999
	Median	884,9	1265	
	Q1-Q3	381.1-2156	541.1-1988	
PINP (pg/ml) at 10 Months	n	5	2	0,846
	Median	652	1563	
	Q1-Q3	447.6-1261	559.0-2567	
PINP (pg/ml) at 12 Months	n	2	0	
	Median	992,5	.	
	Q1-Q3	701.3-1284	.-.	
	**CTX(ng/ml)**			
CTX(ng/ml) at baseline	n	16	8	0,257
	Median	0,6	0,9	
	Q1-Q3	0.2-0.9	0.6-1.0	
CTX(ng/ml) at 2 Months	n	11	6	0,159
	Median	0,3	0,5	
	Q1-Q3	0.1-0.6	0.3-0.8	
CTX(ng/ml) at 4 Months	n	7	4	0,073
	Median	0,2	0,4	
	Q1-Q3	0.2-0.3	0.3-0.6	
CTX(ng/ml) at 6 Months	n	7	2	0,884
	Median	0,2	0,4	
	Q1-Q3	0.1-0.4	0.2-0.6	
CTX(ng/ml) at 8 Months	n	6	2	0,405
	Median	0,1	0,3	
	Q1-Q3	0.1-0.3	0.1-0.5	
CTX(ng/ml) at 10 Months	n	5	2	>0.999
	Median	0,2	0,2	
	Q1-Q3	0.1-0.3	0.1-0.2	
CTX(ng/ml) at 12 Months	n	2	0	
	Median	0,3	.	
	Q1-Q3	0.2-0.4	.-.	
	**TRACP-5B (U/L)**			
TRACP-5B (U/L) at baseline	n	16	8	0,188
	Median	2,4	4	
	Q1-Q3	1.7-3.7	2.3-5.7	
TRACP-5B (U/L) at 2 Months	n	11	6	0,96
	Median	1,9	1,8	
	Q1-Q3	1.0-2.1	0.8-2.4	
TRACP-5B (U/L) at 4 Months	n	7	4	0,777

	Median	1,3	1,2	
	Q1-Q3	0.8-2.0	0.8-1.6	
TRACP-5B (U/L) at 6 Months	n	7	2	0,306
	Median	1,7	1,1	
	Q1-Q3	1.1-1.9	1.0-1.2	
TRACP-5B (U/L) at 8 Months	n	6	2	>0.999
	Median	1	1	
	Q1-Q3	0.8-1.1	1.0-1.0	
TRACP-5B (U/L) at 10 Months	n	3	2	0,773
	Median	0,9	0,9	
	Q1-Q3	0.8-2.0	0.9-0.9	
TRACP-5B (U/L) at 12 Months	n	2	0	
	Median	1,3	.	
	Q1-Q3	0.9-1.8	.-.	
	**RANKL (pmol/L)**			
RANKL (pmol/L) at baseline	n	16	8	0,342
	Median	0,2	0,4	
	Q1-Q3	0.1-0.4	0.3-0.4	
RANKL (pmol/L) at 2 Months	n	11	6	0,209
	Median	0,1	0,2	
	Q1-Q3	0.1-0.2	0.2-0.2	
RANKL (pmol/L) at 4 Months	n	7	4	0,299
	Median	0,1	0,1	
	Q1-Q3	0.1-0.1	0.1-0.1	
RANKL (pmol/L) at 6 Months	n	7	2	0,107
	Median	0,1	0,1	
	Q1-Q3	0.1-0.2	0.1-0.1	
RANKL (pmol/L) at 8 Months	n	6	2	0,067
	Median	0,1	0	
	Q1-Q3	0.1-0.1	0.0-0.0	
RANKL (pmol/L) at 10 Months	n	5	2	0,081
	Median	0,1	0	
	Q1-Q3	0.1-0.2	0.0-0.0	
RANKL (pmol/L) at 12 Months	n	2	0	
	Median	0,1	.	
	Q1-Q3	0.1-0.2	.-.	
	**RANKL/OPG ratio**			
RANKL/OPG ratio at baseline	n	16	8	0,257
	Median	0,1	0,1	
	Q1-Q3	0.0-0.1	0.1-0.1	
RANKL/OPG ratio at 2 Months	n	11	6	0,393
	Median	0	0	
	Q1-Q3	0.0-0.0	0.0-0.0	
RANKL/OPG ratio at 4 Months	n	7	4	0,219
	Median	0	0	
	Q1-Q3	0.0-0.0	0.0-0.0	
RANKL/OPG ratio at 6 Months	n	7	2	0,057
	Median	0	0	
	Q1-Q3	0.0-0.0	0.0-0.0	
RANKL/OPG ratio at 8 Months	n	6	2	0,067

	Median	0	0	
	Q1-Q3	0.0-0.0	0.0-0.0	
RANKL/OPG ratio at 10 Months	n	5	2	0,081
	Median	0	0	
	Q1-Q3	0.0-0.0	0.0-0.0	
RANKL/OPG ratio at 12 Months	n	2	0	
	Median	0	.	
	Q1-Q3	0.0-0.0	.-.	
	**SOST (pmol/L)**			
SOST (pmol/L) at baseline	n	16	8	0,927
	Median	43,3	58,7	
	Q1-Q3	38.0-66.0	36.7-63.5	
SOST (pmol/L) at 2 Months	n	11	6	0,451
	Median	38,2	33,3	
	Q1-Q3	33.8-49.7	27.9-41.7	
SOST (pmol/L) at 4 Months	n	7	4	**0,047**
	Median	43,3	23,9	
	Q1-Q3	32.8-77.1	22.5-30.9	
SOST (pmol/L) at 6 Months	n	7	2	0,107
	Median	48,1	23,5	
	Q1-Q3	25.8-92.9	21.6-25.5	
SOST (pmol/L) at 8 Months	n	6	2	0,067
	Median	37,1	20,8	
	Q1-Q3	26.2-63.5	20.4-21.2	
SOST (pmol/L) at 10 Months	n	5	2	0,081
	Median	40,5	19,8	
	Q1-Q3	36.9-64.7	19.3-20.2	
SOST (pmol/L) at 12 Months	n	2	0	
	Median	27,8	.	
	Q1-Q3	20.0-35.7	.-.	
	**Dkk1 (pmol/L)**			
Dkk1 (pmol/L) at baseline	n	16	8	0,168
	Median	44,9	33,4	
	Q1-Q3	34.7-74.7	20.7-48.3	
Dkk1 (pmol/L) at 2 Months	n	11	6	0,451
	Median	55,3	30,2	
	Q1-Q3	26.9-67.5	24.1-42.0	
Dkk1 (pmol/L) at 4 Months	n	7	4	0,777
	Median	33,7	34,7	
	Q1-Q3	18.5-58.4	18.7-54.7	
Dkk1 (pmol/L) at 6 Months	n	7	2	>0.999
	Median	37	40,6	
	Q1-Q3	21.2-52.4	32.0-49.2	
Dkk1 (pmol/L) at 8 Months	n	6	2	0,405
	Median	26	32,1	
	Q1-Q3	19.2-31.0	29.7-34.4	
Dkk1 (pmol/L) at 10 Months	n	5	2	0,081
	Median	16,3	34,9	
	Q1-Q3	9.1-26.1	31.0-38.7	
Dkk1 (pmol/L) at 12 Months	n	2	0	

	Median	14,4	.	
	Q1-Q3	8.4-20.4	.-.	
	**activin-A (pg/ml)**			
activin-A (pg/ml) at baseline	n	16	8	**0,016**
	Median	758	536,6	
	Q1-Q3	559.9-917.4	281.9-595.1	
activin-A (pg/ml) at 2 Months	n	11	6	0,802
	Median	447,5	482,9	
	Q1-Q3	321.0-562.2	358.2-538.3	
activin-A (pg/ml) at 4 Months	n	7	4	0,108
	Median	468,3	289,5	
	Q1-Q3	412.5-580.9	204.8-423.9	
activin-A (pg/ml) at 6 Months	n	7	2	0,306
	Median	388,3	296,9	
	Q1-Q3	366.9-593.8	215.0-378.7	
activin-A (pg/ml) at 8 Months	n	6	2	0,243
	Median	428,7	275,4	
	Q1-Q3	365.7-537.9	231.9-319.0	
activin-A (pg/ml) at 10 Months	n	5	2	0,175
	Median	362,8	242,4	
	Q1-Q3	357.5-422.7	201.0-283.8	
activin-A (pg/ml) at 12 Months	n	2	0	
	Median	287,5	.	
	Q1-Q3	256.8-318.2	.-.	
	**CCL3 (ng/ml)**			
CCL3 (ng/ml) at baseline	n	16	8	0,52
	Median	75,9	80,4	
	Q1-Q3	42.5-91.4	67.6-95.1	
CCL3 (ng/ml) at 2 Months	n	11	6	0,514
	Median	61,2	73,8	
	Q1-Q3	11.1-97.0	70.5-89.4	
CCL3 (ng/ml) at 4 Months	n	7	4	0,777
	Median	68	66,4	
	Q1-Q3	34.3-82.9	62.3-70.8	
CCL3 (ng/ml) at 6 Months	n	7	2	0,464
	Median	61,3	67,6	
	Q1-Q3	54.0-71.1	62.1-73.1	
CCL3 (ng/ml) at 8 Months	n	6	2	0,067
	Median	54,7	77,9	
	Q1-Q3	21.1-61.1	66.9-88.9	
CCL3 (ng/ml) at 10 Months	n	5	2	0,561
	Median	14,2	54,3	
	Q1-Q3	9.1-56.0	50.7-57.9	
CCL3 (ng/ml) at 12 Months	n	2	0	
	Median	34,1	.	
	Q1-Q3	3.9-64.3	.-.	

[a] Mann-Whitney U test

Table S11. Biomarker values at each timepoint, by refractoriness to last line of therapy

		Refractorinesss to last line of therapy		
		**Yes**	**No**	
		**n (%)**	**n (%)**	**p-value**[a]
	**bALP (µg/L)**			
bALP (µg/L) at baseline	n	13	11	0,385
	Median	11,2	10,5	
	Q1-Q3	10.3-11.7	8.3-11.5	
bALP (µg/L) at 2 Months	n	9	8	0,413
	Median	12,8	10,9	
	Q1-Q3	9.4-17.5	8.5-12.6	
bALP (µg/L) at 4 Months	n	5	6	0,648
	Median	10,8	12,2	
	Q1-Q3	9.1-11.9	11.3-14.1	
bALP (µg/L) at 6 Months	n	6	3	0,053
	Median	9,8	14,8	
	Q1-Q3	7.1-13.6	13.7-19.7	
bALP (µg/L) at 8 Months	n	5	3	**0,037**
	Median	6,8	18	
	Q1-Q3	5.6-15.0	16.9-19.6	
bALP (µg/L) at 10 Months	n	5	2	0,081
	Median	9,8	19	
	Q1-Q3	7.0-15.0	18.1-20.0	
bALP (µg/L) at 12 Months	n	2	0	
	Median	17,1	.	
	Q1-Q3	14.5-19.7	.-.	
	**Osteocalcin (ng/ml)**			
Osteocalcin (ng/ml) at baseline	n	13	11	0,247
	Median	9,1	9,9	
	Q1-Q3	3.9-10.8	6.6-11.9	
Osteocalcin (ng/ml) at 2 Months	n	9	8	0,665
	Median	10,5	11,4	
	Q1-Q3	5.8-14.9	9.1-14.1	
Osteocalcin (ng/ml) at 4 Months	n	5	6	0,523
	Median	17,2	11,7	
	Q1-Q3	12.4-17.7	8.6-19.2	
Osteocalcin (ng/ml) at 6 Months	n	6	3	0,093
	Median	11,9	25,4	
	Q1-Q3	10.8-16.2	13.9-26.7	
Osteocalcin (ng/ml) at 8 Months	n	5	3	0,074
	Median	8,5	29,8	
	Q1-Q3	6.0-14.9	16.9-40.1	
Osteocalcin (ng/ml) at 10 Months	n	5	2	0,081
	Median	8,9	23,9	
	Q1-Q3	3.8-16.8	19.7-28.2	
Osteocalcin (ng/ml) at 12 Months	n	2	0	
	Median	17,1	.	
	Q1-Q3	13.3-20.8	.-.	
	**PINP (pg/ml)**			

PINP (pg/ml) at baseline	n	13	11	0,602
	Median	547,4	467,8	
	Q1-Q3	310.5-654.2	215.8-750.7	
PINP (pg/ml) at 2 Months	n	9	8	0,268
	Median	226,3	467,8	
	Q1-Q3	178.9-775.3	358.7-808.9	
PINP (pg/ml) at 4 Months	n	5	6	0,121
	Median	411,6	557,8	
	Q1-Q3	278.5-490.2	459.5-1567	
PINP (pg/ml) at 6 Months	n	6	3	0,245
	Median	441	1694	
	Q1-Q3	397.8-698.7	419.7-2056	
PINP (pg/ml) at 8 Months	n	5	3	0,551
	Median	765,5	1988	
	Q1-Q3	381.1-1004	541.1-2156	
PINP (pg/ml) at 10 Months	n	5	2	0,846
	Median	652	1563	
	Q1-Q3	447.6-1261	559.0-2567	
PINP (pg/ml) at 12 Months	n	2	0	
	Median	992,5	.	
	Q1-Q3	701.3-1284	.-.	
	**CTX(ng/ml)**			
CTX(ng/ml) at baseline	n	13	11	0,325
	Median	0,6	0,8	
	Q1-Q3	0.2-0.9	0.6-1.0	
CTX(ng/ml) at 2 Months	n	9	8	0,083
	Median	0,2	0,5	
	Q1-Q3	0.1-0.5	0.3-0.7	
CTX(ng/ml) at 4 Months	n	5	6	0,121
	Median	0,2	0,3	
	Q1-Q3	0.2-0.3	0.3-0.5	
CTX(ng/ml) at 6 Months	n	6	3	0,699
	Median	0,2	0,3	
	Q1-Q3	0.1-0.4	0.2-0.6	
CTX(ng/ml) at 8 Months	n	5	3	0,766
	Median	0,2	0,1	
	Q1-Q3	0.1-0.3	0.1-0.5	
CTX(ng/ml) at 10 Months	n	5	2	>0.999
	Median	0,2	0,2	
	Q1-Q3	0.1-0.3	0.1-0.2	
CTX(ng/ml) at 12 Months	n	2	0	
	Median	0,3	.	
	Q1-Q3	0.2-0.4	.-.	
	**TRACP-5B (U/L)**			
TRACP-5B (U/L) at baseline	n	13	11	0,325
	Median	2,2	3,6	
	Q1-Q3	1.8-3.7	1.6-5.0	
TRACP-5B (U/L) at 2 Months	n	9	8	0,532
	Median	1,8	1,9	
	Q1-Q3	1.0-2.0	1.3-2.7	

TRACP-5B (U/L) at 4 Months	n	5	6	0,411
	Median	1	1,6	
	Q1-Q3	0.8-1.3	1.2-2.1	
TRACP-5B (U/L) at 6 Months	n	6	3	0,897
	Median	1,5	1,2	
	Q1-Q3	1.1-1.9	1.0-1.9	
TRACP-5B (U/L) at 8 Months	n	5	3	0,766
	Median	0,9	1	
	Q1-Q3	0.8-1.1	1.0-1.0	
TRACP-5B (U/L) at 10 Months	n	3	2	0,773
	Median	0,9	0,9	
	Q1-Q3	0.8-2.0	0.9-0.9	
TRACP-5B (U/L) at 12 Months	n	2	0	
	Median	1,3	.	
	Q1-Q3	0.9-1.8	.-.	
	**RANKL (pmol/L)**			
RANKL (pmol/L) at baseline	n	13	11	0,543
	Median	0,2	0,3	
	Q1-Q3	0.1-0.5	0.2-0.4	
RANKL (pmol/L) at 2 Months	n	9	8	0,268
	Median	0,1	0,2	
	Q1-Q3	0.1-0.2	0.2-0.2	
RANKL (pmol/L) at 4 Months	n	5	6	0,648
	Median	0,1	0,1	
	Q1-Q3	0.1-0.1	0.1-0.1	
RANKL (pmol/L) at 6 Months	n	6	3	0,366
	Median	0,1	0,1	
	Q1-Q3	0.1-0.2	0.1-0.1	
RANKL (pmol/L) at 8 Months	n	5	3	0,136
	Median	0,1	0	
	Q1-Q3	0.1-0.1	0.0-0.1	
RANKL (pmol/L) at 10 Months	n	5	2	0,081
	Median	0,1	0	
	Q1-Q3	0.1-0.2	0.0-0.0	
RANKL (pmol/L) at 12 Months	n	2	0	
	Median	0,1	.	
	Q1-Q3	0.1-0.2	.-.	
	**RANKL/OPG ratio**			
RANKL/OPG ratio at baseline	n	13	11	0,451
	Median	0,1	0,1	
	Q1-Q3	0.0-0.1	0.1-0.1	
RANKL/OPG ratio at 2 Months	n	9	8	0,597
	Median	0	0	
	Q1-Q3	0.0-0.0	0.0-0.0	
RANKL/OPG ratio at 4 Months	n	5	6	0,523
	Median	0	0	
	Q1-Q3	0.0-0.0	0.0-0.0	
RANKL/OPG ratio at 6 Months	n	6	3	0,156
	Median	0	0	
	Q1-Q3	0.0-0.0	0.0-0.0	

RANKL/OPG ratio at 8 Months	n	5	3	0,136
	Median	0	0	
	Q1-Q3	0.0-0.0	0.0-0.0	
RANKL/OPG ratio at 10 Months	n	5	2	0,081
	Median	0	0	
	Q1-Q3	0.0-0.0	0.0-0.0	
RANKL/OPG ratio at 12 Months	n	2	0	
	Median	0	.	
	Q1-Q3	0.0-0.0	.-.	
	**SOST (pmol/L)**			
SOST (pmol/L) at baseline	n	13	11	>0.999
	Median	44,8	56,2	
	Q1-Q3	38.1-66.3	37.9-64.1	
SOST (pmol/L) at 2 Months	n	9	8	0,47
	Median	40,1	36,7	
	Q1-Q3	33.8-49.7	28.7-40.0	
SOST (pmol/L) at 4 Months	n	5	6	0,083
	Median	45,8	28,9	
	Q1-Q3	33.2-77.1	22.8-36.7	
SOST (pmol/L) at 6 Months	n	6	3	0,093
	Median	55,6	25,5	
	Q1-Q3	31.8-92.9	21.6-25.8	
SOST (pmol/L) at 8 Months	n	5	3	0,074
	Median	44,3	21,2	
	Q1-Q3	29.9-63.5	20.4-26.2	
SOST (pmol/L) at 10 Months	n	5	2	0,081
	Median	40,5	19,8	
	Q1-Q3	36.9-64.7	19.3-20.2	
SOST (pmol/L) at 12 Months	n	2	0	
	Median	27,8	.	
	Q1-Q3	20.0-35.7	.-.	
	**Dkk1 (pmol/L)**			
Dkk1 (pmol/L) at baseline	n	13	11	0,117
	Median	50,9	37,5	
	Q1-Q3	32.0-76.6	25.1-42.0	
Dkk1 (pmol/L) at 2 Months	n	9	8	0,229
	Median	58,4	30,2	
	Q1-Q3	28.7-67.5	24.2-39.4	
Dkk1 (pmol/L) at 4 Months	n	5	6	>0.999
	Median	33,7	37,2	
	Q1-Q3	18.5-57.5	22.8-58.4	
Dkk1 (pmol/L) at 6 Months	n	6	3	0,897
	Median	38,3	34	
	Q1-Q3	21.2-52.4	32.0-49.2	
Dkk1 (pmol/L) at 8 Months	n	5	3	>0.999
	Median	28,2	29,7	
	Q1-Q3	23.8-31.0	5.9-34.4	
Dkk1 (pmol/L) at 10 Months	n	5	2	0,081
	Median	16,3	34,9	
	Q1-Q3	9.1-26.1	31.0-38.7	

Dkk1 (pmol/L) at 12 Months	n	2	0	
	Median	14,4	.	
	Q1-Q3	8.4-20.4	.-.	
	**activin-A (pg/ml)**			
activin-A (pg/ml) at baseline	n	13	11	0,354
	Median	694,3	554,1	
	Q1-Q3	505.0-904.2	286.2-770.8	
activin-A (pg/ml) at 2 Months	n	9	8	0,597
	Median	436,9	482,9	
	Q1-Q3	321.0-562.2	410.2-528.0	
activin-A (pg/ml) at 4 Months	n	5	6	0,171
	Median	468,3	344,9	
	Q1-Q3	418.7-519.6	223.6-492.5	
activin-A (pg/ml) at 6 Months	n	6	3	0,699
	Median	380,5	378,7	
	Q1-Q3	366.9-593.8	215.0-504.5	
activin-A (pg/ml) at 8 Months	n	5	3	0,766
	Median	418,4	319	
	Q1-Q3	365.7-439.0	231.9-537.9	
activin-A (pg/ml) at 10 Months	n	5	2	0,175
	Median	362,8	242,4	
	Q1-Q3	357.5-422.7	201.0-283.8	
activin-A (pg/ml) at 12 Months	n	2	0	
	Median	287,5	.	
	Q1-Q3	256.8-318.2	.-.	
	**CCL3 (ng/ml)**			
CCL3 (ng/ml) at baseline	n	13	11	>0.999
	Median	76,6	79	
	Q1-Q3	70.9-88.1	22.8-98.9	
CCL3 (ng/ml) at 2 Months	n	9	8	>0.999
	Median	61,2	70,8	
	Q1-Q3	44.0-97.0	53.2-82.9	
CCL3 (ng/ml) at 4 Months	n	5	6	0,784
	Median	68	65,6	
	Q1-Q3	47.0-82.9	61.3-69.6	
CCL3 (ng/ml) at 6 Months	n	6	3	0,699
	Median	64,3	62,1	
	Q1-Q3	54.0-71.1	61.3-73.1	
CCL3 (ng/ml) at 8 Months	n	5	3	0,136
	Median	54,3	66,9	
	Q1-Q3	21.1-61.1	55.1-88.9	
CCL3 (ng/ml) at 10 Months	n	5	2	0,561
	Median	14,2	54,3	
	Q1-Q3	9.1-56.0	50.7-57.9	
CCL3 (ng/ml) at 12 Months	n	2	0	
	Median	34,1	.	
	Q1-Q3	3.9-64.3	.-.	

[a] Mann-Whitney U test

Table S12. Biomarker values at each timepoint, by prior use of bisphosphonates

		Prior use of bisphosphonates		
		Yes	No	
		n (%)	n (%)	p-value[a]
	**bALP (µg/L)**			
bALP (µg/L) at baseline	n	18	6	0,102
	Median	10,6	14,6	
	Q1-Q3	8.3-11.5	10.3-21.6	
bALP (µg/L) at 2 Months	n	13	4	0,462
	Median	11,7	14,2	
	Q1-Q3	9.1-12.8	10.3-17.5	
bALP (µg/L) at 4 Months	n	9	2	0,409
	Median	11,3	13	
	Q1-Q3	9.1-12.9	11.9-14.1	
bALP (µg/L) at 6 Months	n	7	2	0,661
	Median	13,6	13,2	
	Q1-Q3	7.1-14.8	11.6-14.8	
bALP (µg/L) at 8 Months	n	7	1	0,19
	Median	15	19,6	
	Q1-Q3	5.6-16.9	19.6-19.6	
bALP (µg/L) at 10 Months	n	7	0	
	Median	15	.	
	Q1-Q3	7.0-18.1	.-.	
bALP (µg/L) at 12 Months	n	2	0	
	Median	17,1	.	
	Q1-Q3	14.5-19.7	.-.	
	**Osteocalcin (ng/ml)**			
Osteocalcin (ng/ml) at baseline	n	18	6	0,117
	Median	7,4	10,6	
	Q1-Q3	3.9-10.8	9.1-17.6	
Osteocalcin (ng/ml) at 2 Months	n	13	4	0,61
	Median	10,5	10,8	
	Q1-Q3	8.8-14.9	5.7-13.2	
Osteocalcin (ng/ml) at 4 Months	n	9	2	0,126
	Median	12,3	19,3	
	Q1-Q3	9.9-17.2	19.2-19.4	
Osteocalcin (ng/ml) at 6 Months	n	7	2	0,107
	Median	12,8	22,8	
	Q1-Q3	10.8-16.2	18.9-26.7	
Osteocalcin (ng/ml) at 8 Months	n	7	1	0,19
	Median	14,9	40,1	
	Q1-Q3	6.0-17.8	40.1-40.1	
Osteocalcin (ng/ml) at 10 Months	n	7	0	
	Median	16,8	.	
	Q1-Q3	3.8-19.7	.-.	
Osteocalcin (ng/ml) at 12 Months	n	2	0	
	Median	17,1	.	
	Q1-Q3	13.3-20.8	.-.	
	**PINP (pg/ml)**			
PINP (pg/ml) at baseline	n	18	6	0,117

	Median	512,4	766,1	
	Q1-Q3	292.1-654.2	362.7-1268	
PINP (pg/ml) at 2 Months	n	13	4	0,692
	Median	384,2	580,1	
	Q1-Q3	226.3-710.7	260.0-1072	
PINP (pg/ml) at 4 Months	n	9	2	0,409
	Median	459,5	1029	
	Q1-Q3	411.6-601.4	490.2-1567	
PINP (pg/ml) at 6 Months	n	7	2	0,661
	Median	442,8	1067	
	Q1-Q3	397.8-789.0	439.3-1694	
PINP (pg/ml) at 8 Months	n	7	1	0,383
	Median	765,5	2156	
	Q1-Q3	381.1-1988	2156-2156	
PINP (pg/ml) at 10 Months	n	7	0	
	Median	652	.	
	Q1-Q3	447.6-2567	.-.	
PINP (pg/ml) at 12 Months	n	2	0	
	Median	992,5	.	
	Q1-Q3	701.3-1284	.-.	
	**CTX(ng/ml)**			
CTX(ng/ml) at baseline	n	18	6	0,527
	Median	0,7	0,7	
	Q1-Q3	0.2-0.9	0.4-1.3	
CTX(ng/ml) at 2 Months	n	13	4	0,692
	Median	0,5	0,3	
	Q1-Q3	0.2-0.6	0.2-0.5	
CTX(ng/ml) at 4 Months	n	9	2	0,724
	Median	0,3	0,3	
	Q1-Q3	0.2-0.5	0.2-0.3	
CTX(ng/ml) at 6 Months	n	7	2	>0.999
	Median	0,2	0,2	
	Q1-Q3	0.1-0.6	0.2-0.3	
CTX(ng/ml) at 8 Months	n	7	1	0,663
	Median	0,2	0,1	
	Q1-Q3	0.1-0.5	0.1-0.1	
CTX(ng/ml) at 10 Months	n	7	0	
	Median	0,2	.	
	Q1-Q3	0.1-0.3	.-.	
CTX(ng/ml) at 12 Months	n	2	0	
	Median	0,3	.	
	Q1-Q3	0.2-0.4	.-.	
	**TRACP-5B (U/L)**			
TRACP-5B (U/L) at baseline	n	18	6	0,405
	Median	3,5	2,4	
	Q1-Q3	1.9-4.0	1.2-3.7	
TRACP-5B (U/L) at 2 Months	n	13	4	0,692
	Median	1,9	1,3	
	Q1-Q3	1.7-2.0	0.6-2.6	
TRACP-5B (U/L) at 4 Months	n	9	2	0,195

	Median	1,2	1,8	
	Q1-Q3	0.8-1.9	1.3-2.2	
TRACP-5B (U/L) at 6 Months	n	7	2	0,464
	Median	1,2	1,6	
	Q1-Q3	1.0-1.9	1.3-1.9	
TRACP-5B (U/L) at 8 Months	n	7	1	0,663
	Median	1	1	
	Q1-Q3	0.8-1.1	1.0-1.0	
TRACP-5B (U/L) at 10 Months	n	5	0	
	Median	0,9	.	
	Q1-Q3	0.9-0.9	.-.	
TRACP-5B (U/L) at 12 Months	n	2	0	
	Median	1,3	.	
	Q1-Q3	0.9-1.8	.-.	
	**RANKL (pmol/L)**			
RANKL (pmol/L) at baseline	n	18	6	0,571
	Median	0,3	0,3	
	Q1-Q3	0.2-0.4	0.1-0.3	
RANKL (pmol/L) at 2 Months	n	13	4	0,692
	Median	0,2	0,1	
	Q1-Q3	0.1-0.2	0.1-0.2	
RANKL (pmol/L) at 4 Months	n	9	2	0,906
	Median	0,1	0,1	
	Q1-Q3	0.1-0.1	0.1-0.1	
RANKL (pmol/L) at 6 Months	n	7	2	0,884
	Median	0,1	0,1	
	Q1-Q3	0.1-0.2	0.1-0.1	
RANKL (pmol/L) at 8 Months	n	7	1	>0.999
	Median	0,1	0,1	
	Q1-Q3	0.0-0.1	0.1-0.1	
RANKL (pmol/L) at 10 Months	n	7	0	
	Median	0,1	.	
	Q1-Q3	0.0-0.2	.-.	
RANKL (pmol/L) at 12 Months	n	2	0	
	Median	0,1	.	
	Q1-Q3	0.1-0.2	.-.	
	**RANKL/OPG ratio**			
RANKL/OPG ratio at baseline	n	18	6	0,617
	Median	0,1	0,1	
	Q1-Q3	0.0-0.1	0.0-0.1	
RANKL/OPG ratio at 2 Months	n	13	4	0,865
	Median	0	0	
	Q1-Q3	0.0-0.0	0.0-0.1	
RANKL/OPG ratio at 4 Months	n	9	2	>0.999
	Median	0	0	
	Q1-Q3	0.0-0.0	0.0-0.0	
RANKL/OPG ratio at 6 Months	n	7	2	>0.999
	Median	0	0	
	Q1-Q3	0.0-0.0	0.0-0.0	
RANKL/OPG ratio at 8 Months	n	7	1	>0.999

	Median	0	0	
	Q1-Q3	0.0-0.0	0.0-0.0	
RANKL/OPG ratio at 10 Months	n	7	0	
	Median	0	.	
	Q1-Q3	0.0-0.0	.-.	
RANKL/OPG ratio at 12 Months	n	2	0	
	Median	0	.	
	Q1-Q3	0.0-0.0	.-.	
	**SOST (pmol/L)**			
SOST (pmol/L) at baseline	n	18	6	0,973
	Median	43,3	53,3	
	Q1-Q3	38.1-65.6	37.9-64.6	
SOST (pmol/L) at 2 Months	n	13	4	0,396
	Median	38,2	28,8	
	Q1-Q3	33.8-41.7	18.1-60.7	
SOST (pmol/L) at 4 Months	n	9	2	0,409
	Median	33,2	65,8	
	Q1-Q3	25.0-43.3	32.8-98.7	
SOST (pmol/L) at 6 Months	n	7	2	0,884
	Median	31,8	44,5	
	Q1-Q3	22.9-92.9	25.8-63.1	
SOST (pmol/L) at 8 Months	n	7	1	>0.999
	Median	29,9	26,2	
	Q1-Q3	21.2-63.5	26.2-26.2	
SOST (pmol/L) at 10 Months	n	7	0	
	Median	36,9	.	
	Q1-Q3	20.2-64.7	.-.	
SOST (pmol/L) at 12 Months	n	2	0	
	Median	27,8	.	
	Q1-Q3	20.0-35.7	.-.	
	**Dkk1 (pmol/L)**			
Dkk1 (pmol/L) at baseline	n	18	6	0,386
	Median	42,5	36,8	
	Q1-Q3	31.4-72.8	16.3-50.9	
Dkk1 (pmol/L) at 2 Months	n	13	4	0,157
	Median	55,3	27,8	
	Q1-Q3	29.1-67.5	25.5-32.8	
Dkk1 (pmol/L) at 4 Months	n	9	2	0,289
	Median	46,5	21,9	
	Q1-Q3	22.8-58.4	15.9-27.8	
Dkk1 (pmol/L) at 6 Months	n	7	2	0,188
	Median	39,7	21,9	
	Q1-Q3	32.0-52.4	9.7-34.0	
Dkk1 (pmol/L) at 8 Months	n	7	1	0,19
	Median	29,7	5,9	
	Q1-Q3	23.8-34.4	5.9-5.9	
Dkk1 (pmol/L) at 10 Months	n	7	0	
	Median	26,1	.	
	Q1-Q3	9.1-31.0	.-.	
Dkk1 (pmol/L) at 12 Months	n	2	0	

	Median	14,4	.	
	Q1-Q3	8.4-20.4	.-.	
	**activin-A (pg/ml)**			
activin-A (pg/ml) at baseline	n	18	6	0,92
	Median	652	646,8	
	Q1-Q3	505.0-902.7	421.1-913.5	
activin-A (pg/ml) at 2 Months	n	13	4	0,955
	Median	447,5	475,3	
	Q1-Q3	358.2-538.3	322.2-525.3	
activin-A (pg/ml) at 4 Months	n	9	2	>0.999
	Median	418,7	427	
	Q1-Q3	355.4-492.5	334.5-519.6	
activin-A (pg/ml) at 6 Months	n	7	2	0,884
	Median	378,7	438,5	
	Q1-Q3	300.0-593.8	372.6-504.5	
activin-A (pg/ml) at 8 Months	n	7	1	0,383
	Median	365,7	537,9	
	Q1-Q3	231.9-439.0	537.9-537.9	
activin-A (pg/ml) at 10 Months	n	7	0	
	Median	357,5	.	
	Q1-Q3	280.5-422.7	.-.	
activin-A (pg/ml) at 12 Months	n	2	0	
	Median	287,5	.	
	Q1-Q3	256.8-318.2	.-.	
	**CCL3 (ng/ml)**			
CCL3 (ng/ml) at baseline	n	18	6	0,571
	Median	75,9	84,2	
	Q1-Q3	62.2-88.5	61.3-98.9	
CCL3 (ng/ml) at 2 Months	n	13	4	0,079
	Median	76,3	42,3	
	Q1-Q3	61.2-95.5	24.7-56.6	
CCL3 (ng/ml) at 4 Months	n	9	2	0,195
	Median	63,3	103,1	
	Q1-Q3	47.0-69.6	68.0-138.2	
CCL3 (ng/ml) at 6 Months	n	7	2	0,464
	Median	62,1	67,8	
	Q1-Q3	54.0-71.1	61.3-74.3	
CCL3 (ng/ml) at 8 Months	n	7	1	>0.999
	Median	61,1	55,1	
	Q1-Q3	21.1-66.9	55.1-55.1	
CCL3 (ng/ml) at 10 Months	n	7	0	
	Median	50,7	.	
	Q1-Q3	9.1-57.9	.-.	
CCL3 (ng/ml) at 12 Months	n	2	0	
	Median	34,1	.	
	Q1-Q3	3.9-64.3	.-.	

[a] Mann-Whitney U test

Table S13. Biomarker values at each timepoint, by prior use of PIs

		Prior use of PIs		
		Yes	No	
		n (%)	n (%)	p-value[a]
	**bALP (µg/L)**			
bALP (µg/L) at baseline	n	21	3	0,861
	Median	11,1	10,5	
	Q1-Q3	9.3-11.7	7.7-17.9	
bALP (µg/L) at 2 Months	n	15	2	0,412
	Median	12,4	9,8	
	Q1-Q3	9.1-17.5	8.0-11.7	
bALP (µg/L) at 4 Months	n	9	2	0,906
	Median	11,6	11,1	
	Q1-Q3	10.8-12.9	7.7-14.4	
bALP (µg/L) at 6 Months	n	8	1	0,175
	Median	12,6	19,7	
	Q1-Q3	7.6-14.3	19.7-19.7	
bALP (µg/L) at 8 Months	n	7	1	0,383
	Median	15	18	
	Q1-Q3	5.6-16.9	18.0-18.0	
bALP (µg/L) at 10 Months	n	6	1	0,211
	Median	12,4	20	
	Q1-Q3	7.0-15.8	20.0-20.0	
bALP (µg/L) at 12 Months	n	2	0	
	Median	17,1	.	
	Q1-Q3	14.5-19.7	.-.	
	**Osteocalcin (ng/ml)**			
Osteocalcin (ng/ml) at baseline	n	21	3	0,600
	Median	9,2	7,4	
	Q1-Q3	5.8-11.2	0.5-11.4	
Osteocalcin (ng/ml) at 2 Months	n	15	2	0,602
	Median	10,9	9,7	
	Q1-Q3	5.8-14.9	8.8-10.5	
Osteocalcin (ng/ml) at 4 Months	n	9	2	0,195
	Median	17,2	9,9	
	Q1-Q3	12.3-19.2	8.6-11.2	
Osteocalcin (ng/ml) at 6 Months	n	8	1	>0.999
	Median	14,5	13,9	
	Q1-Q3	10.9-22.2	13.9-13.9	
Osteocalcin (ng/ml) at 8 Months	n	7	1	>0.999
	Median	14,9	16,9	
	Q1-Q3	6.0-29.8	16.9-16.9	
Osteocalcin (ng/ml) at 10 Months	n	6	1	0,453
	Median	12,8	19,7	
	Q1-Q3	3.8-17.7	19.7-19.7	
Osteocalcin (ng/ml) at 12 Months	n	2	0	
	Median	17,1	.	
	Q1-Q3	13.3-20.8	.-.	
	**PINP (pg/ml)**			
PINP (pg/ml) at baseline	n	21	3	>0.999

	Median	537	733,1	
	Q1-Q3	297.5-742.1	5.5-750.7	
PINP (pg/ml) at 2 Months	n	15	2	0,502
	Median	384,9	690,8	
	Q1-Q3	214.8-775.3	333.2-1048	
PINP (pg/ml) at 4 Months	n	9	2	0,409
	Median	490,2	1178	
	Q1-Q3	411.6-601.4	459.5-1897	
PINP (pg/ml) at 6 Months	n	8	1	0,175
	Median	441	2056	
	Q1-Q3	408.7-743.8	2056-2056	
PINP (pg/ml) at 8 Months	n	7	1	0,663
	Median	765,5	1988	
	Q1-Q3	381.1-2156	1988-1988	
PINP (pg/ml) at 10 Months	n	6	1	0,453
	Median	605,5	2567	
	Q1-Q3	447.6-1261	2567-2567	
PINP (pg/ml) at 12 Months	n	2	0	
	Median	992,5	.	
	Q1-Q3	701.3-1284	.-.	
	**CTX(ng/ml)**			
CTX(ng/ml) at baseline	n	21	3	0,541
	Median	0,7	1	
	Q1-Q3	0.3-0.9	0.1-1.9	
CTX(ng/ml) at 2 Months	n	15	2	0,881
	Median	0,4	0,4	
	Q1-Q3	0.1-0.6	0.3-0.6	
CTX(ng/ml) at 4 Months	n	9	2	0,289
	Median	0,3	0,4	
	Q1-Q3	0.2-0.3	0.3-0.5	
CTX(ng/ml) at 6 Months	n	8	1	0,561
	Median	0,3	0,2	
	Q1-Q3	0.2-0.5	0.2-0.2	
CTX(ng/ml) at 8 Months	n	7	1	>0.999
	Median	0,2	0,1	
	Q1-Q3	0.1-0.5	0.1-0.1	
CTX(ng/ml) at 10 Months	n	6	1	0,803
	Median	0,2	0,1	
	Q1-Q3	0.1-0.3	0.1-0.1	
CTX(ng/ml) at 12 Months	n	2	0	
	Median	0,3	.	
	Q1-Q3	0.2-0.4	.-.	
	**TRACP-5B (U/L)**			
TRACP-5B (U/L) at baseline	n	21	3	0,432
	Median	3,3	5	
	Q1-Q3	1.8-3.7	1.0-7.0	
TRACP-5B (U/L) at 2 Months	n	15	2	0,709
	Median	1,9	2	
	Q1-Q3	1.0-2.1	0.8-3.2	
TRACP-5B (U/L) at 4 Months	n	9	2	0,195

	Median	1,3	0,8	
	Q1-Q3	1.0-2.0	0.5-1.2	
TRACP-5B (U/L) at 6 Months	n	8	1	0,333
	Median	1,5	1	
	Q1-Q3	1.1-1.9	1.0-1.0	
TRACP-5B (U/L) at 8 Months	n	7	1	>0.999
	Median	1	1	
	Q1-Q3	0.8-1.1	1.0-1.0	
TRACP-5B (U/L) at 10 Months	n	4	1	>0.999
	Median	0,9	0,9	
	Q1-Q3	0.8-1.4	0.9-0.9	
TRACP-5B (U/L) at 12 Months	n	2	0	
	Median	1,3	.	
	Q1-Q3	0.9-1.8	.-.	
	**RANKL (pmol/L)**			
RANKL (pmol/L) at baseline	n	21	3	0,793
	Median	0,2	0,3	
	Q1-Q3	0.2-0.4	0.1-0.4	
RANKL (pmol/L) at 2 Months	n	15	2	0,157
	Median	0,2	0,2	
	Q1-Q3	0.1-0.2	0.2-0.3	
RANKL (pmol/L) at 4 Months	n	9	2	0,556
	Median	0,1	0,1	
	Q1-Q3	0.1-0.1	0.1-0.2	
RANKL (pmol/L) at 6 Months	n	8	1	0,561
	Median	0,1	0,1	
	Q1-Q3	0.1-0.1	0.1-0.1	
RANKL (pmol/L) at 8 Months	n	7	1	0,190
	Median	0,1	0	
	Q1-Q3	0.1-0.1	0.0-0.0	
RANKL (pmol/L) at 10 Months	n	6	1	0,453
	Median	0,1	0	
	Q1-Q3	0.1-0.2	0.0-0.0	
RANKL (pmol/L) at 12 Months	n	2	0	
	Median	0,1	.	
	Q1-Q3	0.1-0.2	.-.	
	**RANKL/OPG ratio**			
RANKL/OPG ratio at baseline	n	21	3	>0.999
	Median	0,1	0,1	
	Q1-Q3	0.0-0.1	0.0-0.1	
RANKL/OPG ratio at 2 Months	n	15	2	0,264
	Median	0	0	
	Q1-Q3	0.0-0.0	0.0-0.0	
RANKL/OPG ratio at 4 Months	n	9	2	0,724
	Median	0	0	
	Q1-Q3	0.0-0.0	0.0-0.0	
RANKL/OPG ratio at 6 Months	n	8	1	0,333
	Median	0	0	
	Q1-Q3	0.0-0.0	0.0-0.0	
RANKL/OPG ratio at 8 Months	n	7	1	0,190

	Median	0	0	
	Q1-Q3	0.0-0.0	0.0-0.0	
RANKL/OPG ratio at 10 Months	n	6	1	0,453
	Median	0	0	
	Q1-Q3	0.0-0.0	0.0-0.0	
RANKL/OPG ratio at 12 Months	n	2	0	
	Median	0	.	
	Q1-Q3	0.0-0.0	.-.	
	**SOST (pmol/L)**			
SOST (pmol/L) at baseline	n	21	3	0,163
	Median	50,3	33,2	
	Q1-Q3	39.4-65.6	32.4-56.2	
SOST (pmol/L) at 2 Months	n	15	2	0,502
	Median	38,2	32,6	
	Q1-Q3	29.4-48.3	27.9-37.2	
SOST (pmol/L) at 4 Months	n	9	2	0,556
	Median	33,2	29,8	
	Q1-Q3	29.7-45.8	22.8-36.7	
SOST (pmol/L) at 6 Months	n	8	1	0,561
	Median	40	25,5	
	Q1-Q3	24.4-78.0	25.5-25.5	
SOST (pmol/L) at 8 Months	n	7	1	0,383
	Median	29,9	21,2	
	Q1-Q3	23.5-63.5	21.2-21.2	
SOST (pmol/L) at 10 Months	n	6	1	0,453
	Median	38,7	20,2	
	Q1-Q3	21.1-64.7	20.2-20.2	
SOST (pmol/L) at 12 Months	n	2	0	
	Median	27,8	.	
	Q1-Q3	20.0-35.7	.-.	
	**Dkk1 (pmol/L)**			
Dkk1 (pmol/L) at baseline	n	21	3	0,485
	Median	42	35,5	
	Q1-Q3	31.4-72.8	16.3-54.7	
Dkk1 (pmol/L) at 2 Months	n	15	2	0,941
	Median	36,9	35,6	
	Q1-Q3	24.4-67.5	29.1-42.0	
Dkk1 (pmol/L) at 4 Months	n	9	2	0,409
	Median	33,7	30,6	
	Q1-Q3	22.8-58.4	14.6-46.5	
Dkk1 (pmol/L) at 6 Months	n	8	1	0,561
	Median	35,5	49,2	
	Q1-Q3	26.6-46.1	49.2-49.2	
Dkk1 (pmol/L) at 8 Months	n	7	1	>0.999
	Median	28,2	29,7	
	Q1-Q3	19.2-34.4	29.7-29.7	
Dkk1 (pmol/L) at 10 Months	n	6	1	0,211
	Median	21,2	38,7	
	Q1-Q3	9.1-28.7	38.7-38.7	
Dkk1 (pmol/L) at 12 Months	n	2	0	

	Median	14,4	.	
	Q1-Q3	8.4-20.4	.-.	
	**activin-A (pg/ml)**			
activin-A (pg/ml) at baseline	n	21	3	0,138
	Median	694,3	524,9	
	Q1-Q3	505.0-904.2	286.2-548.3	
activin-A (pg/ml) at 2 Months	n	15	2	0,157
	Median	477,3	336,9	
	Q1-Q3	390.4-562.2	315.5-358.2	
activin-A (pg/ml) at 4 Months	n	9	2	0,195
	Median	468,3	289,5	
	Q1-Q3	412.5-519.6	223.6-355.4	
activin-A (pg/ml) at 6 Months	n	8	1	0,175
	Median	383,5	215	
	Q1-Q3	369.7-549.1	215.0-215.0	
activin-A (pg/ml) at 8 Months	n	7	1	0,663
	Median	418,4	319	
	Q1-Q3	231.9-537.9	319.0-319.0	
activin-A (pg/ml) at 10 Months	n	6	1	0,211
	Median	360,2	201	
	Q1-Q3	283.8-422.7	201.0-201.0	
activin-A (pg/ml) at 12 Months	n	2	0	
	Median	287,5	.	
	Q1-Q3	256.8-318.2	.-.	
	**CCL3 (ng/ml)**			
CCL3 (ng/ml) at baseline	n	21	3	0,337
	Median	76,6	81,9	
	Q1-Q3	61.3-88.5	73.9-105.8	
CCL3 (ng/ml) at 2 Months	n	15	2	0,941
	Median	67,4	70,8	
	Q1-Q3	39.0-95.5	70.5-71.2	
CCL3 (ng/ml) at 4 Months	n	9	2	0,906
	Median	68	66,4	
	Q1-Q3	47.0-72.0	63.3-69.6	
CCL3 (ng/ml) at 6 Months	n	8	1	>0.999
	Median	64,4	62,1	
	Q1-Q3	57.6-72.1	62.1-62.1	
CCL3 (ng/ml) at 8 Months	n	7	1	0,383
	Median	55,1	66,9	
	Q1-Q3	21.1-63.5	66.9-66.9	
CCL3 (ng/ml) at 10 Months	n	6	1	0,453
	Median	32,4	57,9	
	Q1-Q3	9.1-56.0	57.9-57.9	
CCL3 (ng/ml) at 12 Months	n	2	0	
	Median	34,1	.	
	Q1-Q3	3.9-64.3	.-.	

[a] Mann-Whitney U test

Table S14. Biomarker values at each timepoint, by lytic bone lesions at Kd initiation

		Lytic bone lesions at Kd initiation				
		**None**	**1-3**	**4-10**	**>10**	
		**n (%)**	**n (%)**	**n (%)**	**n (%)**	**p-value[a]**
		**bALP (µg/L)**				
bALP (µg/L) at baseline	n	4	5	7	8	0,415
	Median	16,4	11,2	10,5	10,4	
	Q1-Q3	10.1-27.3	10.7-11.7	7.8-11.7	9.2-11.3	
bALP (µg/L) at 2 Months	n	4	1	6	6	0,825
	Median	11,5	10,1	11,9	12,6	
	Q1-Q3	7.6-17.5	10.1-10.1	9.1-12.9	9.4-19.1	
bALP (µg/L) at 4 Months	n	2	0	4	5	0,817
	Median	10,5	.	12,8	11,3	
	Q1-Q3	9.1-11.9	.-.	10.2-14.3	10.8-12.9	
bALP (µg/L) at 6 Months	n	2	0	4	3	0,638
	Median	9,8	.	14,2	13,7	
	Q1-Q3	8.1-11.6	.-.	10.2-17.3	7.1-14.8	
bALP (µg/L) at 8 Months	n	1	0	4	3	0,297
	Median	3,8	.	16,5	16,9	
	Q1-Q3	3.8-3.8	.-.	10.3-18.8	6.8-16.9	
bALP (µg/L) at 10 Months	n	1	0	3	3	0,565
	Median	7	.	15	15,8	
	Q1-Q3	7.0-7.0	.-.	5.9-20.0	9.8-18.1	
bALP (µg/L) at 12 Months	n	0	0	1	1	
	Median	.	.	14,5	19,7	
	Q1-Q3	.-.	.-.	14.5-14.5	19.7-19.7	
		**Osteocalcin (ng/ml)**				
Osteocalcin (ng/ml) at baseline	n	4	5	7	8	0,447
	Median	14,2	10,1	7,5	7,9	
	Q1-Q3	8.6-22.2	5.8-11.4	2.9-10.8	4.6-10.6	
Osteocalcin (ng/ml) at 2 Months	n	4	1	6	6	0,748
	Median	11,7	14,1	10	10,7	
	Q1-Q3	5.7-15.9	14.1-14.1	8.8-12.2	5.8-20.4	

Osteocalcin (ng/ml) at 4 Months	n	2	0	4	5	0,493
	Median	18,3	.	11,7	12,4	
	Q1-Q3	17.2-19.4	.-.	10.5-15.7	8.6-17.7	
Osteocalcin (ng/ml) at 6 Months	n	2	0	4	3	0,737
	Median	11,4	.	15,1	12,8	
	Q1-Q3	4.0-18.9	.-.	12.5-21.4	10.8-25.4	
Osteocalcin (ng/ml) at 8 Months	n	1	0	4	3	0,4
	Median	6	.	17,4	14,9	
	Q1-Q3	6.0-6.0	.-.	12.7-29.0	5.6-29.8	
Osteocalcin (ng/ml) at 10 Months	n	1	0	3	3	0,319
	Median	2,8	.	17,7	16,8	
	Q1-Q3	2.8-2.8	.-.	3.8-19.7	8.9-28.2	
Osteocalcin (ng/ml) at 12 Months	n	0	0	1	1	
	Median	.	.	20,8	13,3	
	Q1-Q3	.-.	.-.	20.8-20.8	13.3-13.3	
		**PINP (pg/ml)**				
PINP (pg/ml) at baseline	n	4	5	7	8	0,922
	Median	572,1	487,7	572,8	565,3	
	Q1-Q3	263.1-1055	467.8-537.0	297.5-733.1	234.5-761.2	
PINP (pg/ml) at 2 Months	n	4	1	6	6	0,714
	Median	281,9	550,8	581,9	451,3	
	Q1-Q3	157.0-580.1	550.8-550.8	214.8-1048	229.5-710.7	
PINP (pg/ml) at 4 Months	n	2	0	4	5	0,252
	Median	362,7	.	1084	459,5	
	Q1-Q3	235.2-490.2	.-.	506.5-1732	439.2-514.2	
PINP (pg/ml) at 6 Months	n	2	0	4	3	0,233
	Median	267,4	.	1242	442,8	
	Q1-Q3	95.6-439.3	.-.	593.4-1875	419.7-698.7	
PINP (pg/ml) at 8 Months	n	1	0	4	3	0,311
	Median	198,9	.	1496	765,5	
	Q1-Q3	198.9-198.9	.-.	692.8-2072	541.1-3505	
PINP (pg/ml) at 10 Months	n	1	0	3	3	0,565
	Median	447,6	.	1261	652	

	Q1-Q3	447.6-447.6	.-.	335.8-2567	559.0-3650	
PINP (pg/ml) at 12 Months	n	0	0	1	1	
	Median	.	.	1284	701,3	
	Q1-Q3	.-.	.-.	1284-1284	701.3-701.3	
		**CTX(ng/ml)**				
CTX(ng/ml) at baseline	n	4	5	7	8	0,645
	Median	0,4	0,9	0,8	0,6	
	Q1-Q3	0.2-0.9	0.7-1.2	0.2-0.9	0.3-0.9	
CTX(ng/ml) at 2 Months	n	4	1	6	6	0,14
	Median	0,2	0,8	0,5	0,5	
	Q1-Q3	0.1-0.3	0.8-0.8	0.1-0.6	0.3-0.7	
CTX(ng/ml) at 4 Months	n	2	0	4	5	0,161
	Median	0,1	.	0,3	0,5	
	Q1-Q3	0.1-0.2	.-.	0.3-0.3	0.2-0.7	
CTX(ng/ml) at 6 Months	n	2	0	4	3	0,3
	Median	0,2	.	0,3	0,6	
	Q1-Q3	0.1-0.2	.-.	0.2-0.4	0.1-0.7	
CTX(ng/ml) at 8 Months	n	1	0	4	3	0,683
	Median	0,2	.	0,1	0,5	
	Q1-Q3	0.2-0.2	.-.	0.1-0.2	0.0-0.5	
CTX(ng/ml) at 10 Months	n	1	0	3	3	0,931
	Median	0,2	.	0,1	0,2	
	Q1-Q3	0.2-0.2	.-.	0.1-0.3	0.1-0.4	
CTX(ng/ml) at 12 Months	n	0	0	1	1	
	Median	.	.	0,2	0,4	
	Q1-Q3	.-.	.-.	0.2-0.2	0.4-0.4	
		**TRACP-5B (U/L)**				
TRACP-5B (U/L) at baseline	n	4	5	7	8	0,18
	Median	1,7	3,9	3,3	2,6	
	Q1-Q3	1.3-2.8	3.6-4.8	2.2-4.0	1.3-4.0	
TRACP-5B (U/L) at 2 Months	n	4	1	6	6	0,138
	Median	0,6	1,9	2,2	1,8	
	Q1-Q3	0.6-1.4	1.9-1.9	1.8-3.1	1.0-1.9	

TRACP-5B (U/L) at 4 Months	n	2	0	4	5	0,734
	Median	1	.	1,6	1,2	
	Q1-Q3	0.8-1.3	.-.	1.0-2.2	1.0-1.9	
TRACP-5B (U/L) at 6 Months	n	2	0	4	3	0,737
	Median	1,6	.	1,4	1,2	
	Q1-Q3	1.3-1.9	.-.	0.9-1.8	1.1-15.9	
TRACP-5B (U/L) at 8 Months	n	1	0	4	3	0,297
	Median	0,6	.	1	1	
	Q1-Q3	0.6-0.6	.-.	1.0-1.1	0.8-2.2	
TRACP-5B (U/L) at 10 Months	n	0	0	3	2	0,083
	Median	.	.	0,9	1,4	
	Q1-Q3	.-.	.-.	0.8-0.9	0.9-2.0	
TRACP-5B (U/L) at 12 Months	n	0	0	1	1	
	Median	.	.	0,9	1,8	
	Q1-Q3	.-.	.-.	0.9-0.9	1.8-1.8	
		**RANKL (pmol/L)**				
RANKL (pmol/L) at baseline	n	4	5	7	8	0,969
	Median	0,2	0,2	0,4	0,3	
	Q1-Q3	0.2-0.4	0.2-0.3	0.1-0.5	0.1-0.5	
RANKL (pmol/L) at 2 Months	n	4	1	6	6	0,732
	Median	0,1	0,2	0,2	0,2	
	Q1-Q3	0.1-0.2	0.2-0.2	0.2-0.2	0.1-0.2	
RANKL (pmol/L) at 4 Months	n	2	0	4	5	0,763
	Median	0,1	.	0,1	0,1	
	Q1-Q3	0.1-0.1	.-.	0.1-0.1	0.1-0.2	
RANKL (pmol/L) at 6 Months	n	2	0	4	3	0,472
	Median	0,1	.	0,1	0,2	
	Q1-Q3	0.1-0.1	.-.	0.1-0.1	0.1-0.2	
RANKL (pmol/L) at 8 Months	n	1	0	4	3	0,806
	Median	0,1	.	0,1	0,1	
	Q1-Q3	0.1-0.1	.-.	0.0-0.1	0.0-0.2	
RANKL (pmol/L) at 10 Months	n	1	0	3	3	0,867
	Median	0,1	.	0,1	0,1	

	Q1-Q3	0.1-0.1	.-.	0.0-0.2	0.0-0.3	
RANKL (pmol/L) at 12 Months	n	0	0	1	1	
	Median	.	.	0,1	0,2	
	Q1-Q3	.-.	.-.	0.1-0.1	0.2-0.2	
		**RANKL/OPG ratio**				
RANKL/OPG ratio at baseline	n	4	5	7	8	0,873
	Median	0,1	0,1	0,1	0,1	
	Q1-Q3	0.0-0.1	0.1-0.1	0.0-0.1	0.0-0.1	
RANKL/OPG ratio at 2 Months	n	4	1	6	6	0,954
	Median	0	0	0	0	
	Q1-Q3	0.0-0.1	0.0-0.0	0.0-0.0	0.0-0.0	
RANKL/OPG ratio at 4 Months	n	2	0	4	5	0,861
	Median	0	.	0	0	
	Q1-Q3	0.0-0.0	.-.	0.0-0.0	0.0-0.0	
RANKL/OPG ratio at 6 Months	n	2	0	4	3	0,607
	Median	0	.	0	0	
	Q1-Q3	0.0-0.0	.-.	0.0-0.0	0.0-0.0	
RANKL/OPG ratio at 8 Months	n	1	0	4	3	0,297
	Median	0	.	0	0	
	Q1-Q3	0.0-0.0	.-.	0.0-0.0	0.0-0.0	
RANKL/OPG ratio at 10 Months	n	1	0	3	3	0,931
	Median	0	.	0	0	
	Q1-Q3	0.0-0.0	.-.	0.0-0.0	0.0-0.1	
RANKL/OPG ratio at 12 Months	n	0	0	1	1	
	Median	.	.	0	0	
	Q1-Q3	.-.	.-.	0.0-0.0	0.0-0.0	
		**SOST (pmol/L)**				
SOST (pmol/L) at baseline	n	4	5	7	8	0,205
	Median	67,6	41,8	38,1	52,2	
	Q1-Q3	57.4-80.2	41.4-56.2	33.2-62.9	35.9-66.0	
SOST (pmol/L) at 2 Months	n	4	1	6	6	0,779
	Median	53,3	48,3	35,1	37,7	
	Q1-Q3	18.1-85.2	48.3-48.3	33.8-40.7	29.4-40.1	

SOST (pmol/L) at 4 Months	n	2	0	4	5	0,145
	Median	66	.	27,4	43,3	
	Q1-Q3	33.2-98.7	.-.	23.9-31.2	36.7-45.8	
SOST (pmol/L) at 6 Months	n	2	0	4	3	0,946
	Median	43	.	28,8	48,1	
	Q1-Q3	22.9-63.1	.-.	25.6-62.3	21.6-149.6	
SOST (pmol/L) at 8 Months	n	1	0	4	3	0,806
	Median	44,3	.	24,8	29,9	
	Q1-Q3	44.3-44.3	.-.	22.3-69.7	20.4-63.5	
SOST (pmol/L) at 10 Months	n	1	0	3	3	0,565
	Median	64,7	.	21,1	36,9	
	Q1-Q3	64.7-64.7	.-.	20.2-40.5	19.3-82.1	
SOST (pmol/L) at 12 Months	n	0	0	1	1	
	Median	.	.	20	35,7	
	Q1-Q3	.-.	.-.	20.0-20.0	35.7-35.7	
		**Dkk1 (pmol/L)**				
Dkk1 (pmol/L) at baseline	n	4	5	7	8	0,475
	Median	63,8	35,5	52,4	41,6	
	Q1-Q3	33.6-76.6	31.4-37.5	32.0-75.9	20.7-57.9	
Dkk1 (pmol/L) at 2 Months	n	4	1	6	6	0,431
	Median	27,8	22,7	52,3	43,3	
	Q1-Q3	25.5-54.0	22.7-22.7	36.9-67.5	29.1-58.4	
Dkk1 (pmol/L) at 4 Months	n	2	0	4	5	0,932
	Median	39,6	.	37,2	33,7	
	Q1-Q3	15.9-63.3	.-.	23.1-54.7	22.8-57.5	
Dkk1 (pmol/L) at 6 Months	n	2	0	4	3	0,741
	Median	31,1	.	35,5	39,7	
	Q1-Q3	9.7-52.4	.-.	27.6-43.1	32.0-54.8	
Dkk1 (pmol/L) at 8 Months	n	1	0	4	3	0,092
	Median	51,3	.	21,5	31	
	Q1-Q3	51.3-51.3	.-.	12.6-26.8	28.2-34.4	
Dkk1 (pmol/L) at 10 Months	n	1	0	3	3	0,751
	Median	28,7	.	9,1	26,1	

	Q1-Q3	28.7-28.7	.-.	8.4-38.7	16.3-31.0	
Dkk1 (pmol/L) at 12 Months	n	0	0	1	1	
	Median	.	.	8,4	20,4	
	Q1-Q3	.-.	.-.	8.4-8.4	20.4-20.4	
		**activin-A (pg/ml)**				
activin-A (pg/ml) at baseline	n	4	5	7	8	0,449
	Median	456,7	694,3	554,1	740,3	
	Q1-Q3	349.3-834.4	636.1-904.2	505.0-902.7	477.1-846.0	
activin-A (pg/ml) at 2 Months	n	4	1	6	6	0,568
	Median	462,6	477,3	418,9	572,8	
	Q1-Q3	309.5-525.3	477.3-477.3	321.0-462.2	358.2-786.7	
activin-A (pg/ml) at 4 Months	n	2	0	4	5	0,417
	Median	466,1	.	279	468,3	
	Q1-Q3	412.5-519.6	.-.	204.8-619.6	418.7-492.5	
activin-A (pg/ml) at 6 Months	n	2	0	4	3	0,856
	Median	380,5	.	402,2	378,7	
	Q1-Q3	372.6-388.3	.-.	257.5-549.1	366.9-695.8	
activin-A (pg/ml) at 8 Months	n	1	0	4	3	0,953
	Median	365,7	.	428,4	418,4	
	Q1-Q3	365.7-365.7	.-.	266.2-760.3	231.9-439.0	
activin-A (pg/ml) at 10 Months	n	1	0	3	3	>0.999
	Median	357,5	.	362,8	283,8	
	Q1-Q3	357.5-357.5	.-.	201.0-422.7	280.5-467.5	
activin-A (pg/ml) at 12 Months	n	0	0	1	1	
	Median	.	.	256,8	318,2	
	Q1-Q3	.-.	.-.	256.8-256.8	318.2-318.2	
		**CCL3 (ng/ml)**				
CCL3 (ng/ml) at baseline	n	4	5	7	8	0,399
	Median	74,7	101,8	75,3	75,7	
	Q1-Q3	42.0-94.5	81.3-105.8	62.2-94.8	46.9-81.1	
CCL3 (ng/ml) at 2 Months	n	4	1	6	6	0,584
	Median	42,3	95,5	69,3	79,9	
	Q1-Q3	24.7-71.3	95.5-95.5	61.2-76.3	11.1-102.7	

CCL3 (ng/ml) at 4 Months	n	2	0	4	5	0,097
	Median	110,6	.	64,6	63,3	
	Q1-Q3	82.9-138.2	.-.	54.1-68.8	34.3-68.0	
CCL3 (ng/ml) at 6 Months	n	2	0	4	3	0,197
	Median	72,7	.	61,7	54	
	Q1-Q3	71.1-74.3	.-.	61.3-64.8	6.3-73.1	
CCL3 (ng/ml) at 8 Months	n	1	0	4	3	0,757
	Median	63,5	.	54,7	61,1	
	Q1-Q3	63.5-63.5	.-.	37.7-61.0	8.7-88.9	
CCL3 (ng/ml) at 10 Months	n	1	0	3	3	0,565
	Median	56	.	57,9	14,2	
	Q1-Q3	56.0-56.0	.-.	3.3-60.8	9.1-50.7	
CCL3 (ng/ml) at 12 Months	n	0	0	1	1	
	Median	.	.	64,3	3,9	
	Q1-Q3	.-.	.-.	64.3-64.3	3.9-3.9	

[a] Kruskal-Wallis test

Table S15. Biomarker values at each timepoint, by history of SREs

		History of SREs		
		Yes	No	
		n (%)	n (%)	p-value[a]
	**bALP (µg/L)**			
bALP (µg/L) at baseline	n	9	15	0,633
	Median	11,1	10,7	
	Q1-Q3	10.0-11.7	8.3-11.5	
bALP (µg/L) at 2 Months	n	5	12	0,635
	Median	12,1	12	
	Q1-Q3	9.4-19.1	8.5-14.2	
bALP (µg/L) at 4 Months	n	3	8	0,759
	Median	11,3	11,7	
	Q1-Q3	10.8-28.2	9.0-13.5	
bALP (µg/L) at 6 Months	n	3	6	0,897
	Median	13,6	12,7	
	Q1-Q3	7.1-14.8	8.1-14.8	
bALP (µg/L) at 8 Months	n	3	5	0,766
	Median	15	16,9	
	Q1-Q3	6.8-16.9	5.6-18.0	
bALP (µg/L) at 10 Months	n	3	4	>0.999
	Median	15	12,5	
	Q1-Q3	9.8-15.8	6.4-19.0	
bALP (µg/L) at 12 Months	n	2	0	
	Median	17,1	.	
	Q1-Q3	14.5-19.7	.-.	
	**Osteocalcin (ng/ml)**			
Osteocalcin (ng/ml) at baseline	n	9	15	0,592
	Median	10,1	7,5	
	Q1-Q3	6.6-11.2	3.9-11.9	
Osteocalcin (ng/ml) at 2 Months	n	5	12	0,562
	Median	10,9	10	
	Q1-Q3	10.5-14.9	7.3-14.1	
Osteocalcin (ng/ml) at 4 Months	n	3	8	0,61
	Median	12,4	14,7	
	Q1-Q3	1.0-17.7	10.5-19.3	
Osteocalcin (ng/ml) at 6 Months	n	3	6	0,519
	Median	12,8	16,4	
	Q1-Q3	10.8-16.2	11.1-25.4	
Osteocalcin (ng/ml) at 8 Months	n	3	5	0,551
	Median	14,9	16,9	
	Q1-Q3	5.6-17.8	8.5-29.8	
Osteocalcin (ng/ml) at 10 Months	n	3	4	>0.999
	Median	16,8	11,7	
	Q1-Q3	8.9-17.7	3.3-23.9	
Osteocalcin (ng/ml) at 12 Months	n	2	0	
	Median	17,1	.	
	Q1-Q3	13.3-20.8	.-.	
	**PINP (pg/ml)**			
PINP (pg/ml) at baseline	n	9	15	**0,049**

	Median	654,2	362,7	
	Q1-Q3	572.8-750.7	215.8-733.1	
PINP (pg/ml) at 2 Months	n	5	12	0,958
	Median	569,4	384,6	
	Q1-Q3	226.3-710.7	204.2-897.9	
PINP (pg/ml) at 4 Months	n	3	8	>0.999
	Median	514,2	474,8	
	Q1-Q3	278.5-777.5	425.4-1084	
PINP (pg/ml) at 6 Months	n	3	6	0,519
	Median	698,7	429,5	
	Q1-Q3	442.8-789.0	397.8-1694	
PINP (pg/ml) at 8 Months	n	3	5	0,371
	Median	1004	541,1	
	Q1-Q3	765.5-3505	381.1-1988	
PINP (pg/ml) at 10 Months	n	3	4	0,216
	Median	1261	503,3	
	Q1-Q3	652.0-3650	391.7-1563	
PINP (pg/ml) at 12 Months	n	2	0	
	Median	992,5	.	
	Q1-Q3	701.3-1284	.-.	
	**CTX(ng/ml)**			
CTX(ng/ml) at baseline	n	9	15	0,404
	Median	0,8	0,7	
	Q1-Q3	0.6-1.2	0.2-0.9	
CTX(ng/ml) at 2 Months	n	5	12	0,874
	Median	0,3	0,4	
	Q1-Q3	0.2-0.5	0.2-0.6	
CTX(ng/ml) at 4 Months	n	3	8	0,759
	Median	0,2	0,3	
	Q1-Q3	0.2-0.7	0.2-0.4	
CTX(ng/ml) at 6 Months	n	3	6	0,519
	Median	0,4	0,2	
	Q1-Q3	0.1-0.7	0.2-0.3	
CTX(ng/ml) at 8 Months	n	3	5	>0.999
	Median	0,3	0,1	
	Q1-Q3	0.0-0.5	0.1-0.2	
CTX(ng/ml) at 10 Months	n	3	4	0,596
	Median	0,3	0,2	
	Q1-Q3	0.1-0.4	0.1-0.2	
CTX(ng/ml) at 12 Months	n	2	0	
	Median	0,3	.	
	Q1-Q3	0.2-0.4	.-.	
	**TRACP-5B (U/L)**			
TRACP-5B (U/L) at baseline	n	9	15	0,283
	Median	3,6	3,3	
	Q1-Q3	1.9-4.8	1.5-3.7	
TRACP-5B (U/L) at 2 Months	n	5	12	0,874
	Median	1,9	1,9	
	Q1-Q3	1.7-2.0	0.7-2.2	
TRACP-5B (U/L) at 4 Months	n	3	8	0,61

	Median	1,9	1,2	
	Q1-Q3	1.0-2.0	0.7-1.7	
TRACP-5B (U/L) at 6 Months	n	3	6	0,519
	Median	1,7	1,2	
	Q1-Q3	1.1-15.9	1.0-1.9	
TRACP-5B (U/L) at 8 Months	n	3	5	>0.999
	Median	0,9	1	
	Q1-Q3	0.8-2.2	1.0-1.0	
TRACP-5B (U/L) at 10 Months	n	2	3	0,773
	Median	1,4	0,9	
	Q1-Q3	0.9-2.0	0.8-0.9	
TRACP-5B (U/L) at 12 Months	n	2	0	
	Median	1,3	.	
	Q1-Q3	0.9-1.8	.-.	
	**RANKL (pmol/L)**			
RANKL (pmol/L) at baseline	n	9	15	0,551
	Median	0,3	0,2	
	Q1-Q3	0.2-0.4	0.1-0.4	
RANKL (pmol/L) at 2 Months	n	5	12	0,958
	Median	0,2	0,2	
	Q1-Q3	0.1-0.2	0.1-0.2	
RANKL (pmol/L) at 4 Months	n	3	8	0,358
	Median	0,1	0,1	
	Q1-Q3	0.1-0.2	0.1-0.1	
RANKL (pmol/L) at 6 Months	n	3	6	0,093
	Median	0,2	0,1	
	Q1-Q3	0.1-0.2	0.1-0.1	
RANKL (pmol/L) at 8 Months	n	3	5	0,766
	Median	0,1	0,1	
	Q1-Q3	0.1-0.2	0.0-0.1	
RANKL (pmol/L) at 10 Months	n	3	4	0,596
	Median	0,1	0,1	
	Q1-Q3	0.1-0.3	0.0-0.1	
RANKL (pmol/L) at 12 Months	n	2	0	
	Median	0,1	.	
	Q1-Q3	0.1-0.2	.-.	
	**RANKL/OPG ratio**			
RANKL/OPG ratio at baseline	n	9	15	0,633
	Median	0,1	0,1	
	Q1-Q3	0.0-0.1	0.0-0.1	
RANKL/OPG ratio at 2 Months	n	5	12	0,792
	Median	0	0	
	Q1-Q3	0.0-0.1	0.0-0.0	
RANKL/OPG ratio at 4 Months	n	3	8	0,475
	Median	0	0	
	Q1-Q3	0.0-0.0	0.0-0.0	
RANKL/OPG ratio at 6 Months	n	3	6	0,245
	Median	0	0	
	Q1-Q3	0.0-0.0	0.0-0.0	
RANKL/OPG ratio at 8 Months	n	3	5	>0.999

	Median	0	0	
	Q1-Q3	0.0-0.0	0.0-0.0	
RANKL/OPG ratio at 10 Months	n	3	4	0,377
	Median	0	0	
	Q1-Q3	0.0-0.1	0.0-0.0	
RANKL/OPG ratio at 12 Months	n	2	0	
	Median	0	.	
	Q1-Q3	0.0-0.0	.-.	
	**SOST (pmol/L)**			
SOST (pmol/L) at baseline	n	9	15	0,107
	Median	64,1	40,2	
	Q1-Q3	44.8-66.3	37.7-62.9	
SOST (pmol/L) at 2 Months	n	5	12	0,712
	Median	38,2	36,7	
	Q1-Q3	34.0-40.1	25.0-45.0	
SOST (pmol/L) at 4 Months	n	3	8	0,083
	Median	45,8	31,2	
	Q1-Q3	43.3-77.1	23.9-35.0	
SOST (pmol/L) at 6 Months	n	3	6	0,245
	Median	48,1	25,6	
	Q1-Q3	31.8-149.6	22.9-63.1	
SOST (pmol/L) at 8 Months	n	3	5	0,766
	Median	29,9	26,2	
	Q1-Q3	23.5-63.5	21.2-44.3	
SOST (pmol/L) at 10 Months	n	3	4	0,596
	Median	36,9	30,4	
	Q1-Q3	21.1-82.1	19.8-52.6	
SOST (pmol/L) at 12 Months	n	2	0	
	Median	27,8	.	
	Q1-Q3	20.0-35.7	.-.	
	**Dkk1 (pmol/L)**			
Dkk1 (pmol/L) at baseline	n	9	15	0,591
	Median	35,5	43	
	Q1-Q3	25.1-52.4	31.4-75.9	
Dkk1 (pmol/L) at 2 Months	n	5	12	0,712
	Median	55,3	34,1	
	Q1-Q3	24.4-58.4	27.8-69.8	
Dkk1 (pmol/L) at 4 Months	n	3	8	0,358
	Median	57,5	25,3	
	Q1-Q3	33.7-58.4	17.2-54.7	
Dkk1 (pmol/L) at 6 Months	n	3	6	0,699
	Median	39,7	35,5	
	Q1-Q3	21.2-54.8	32.0-49.2	
Dkk1 (pmol/L) at 8 Months	n	3	5	0,766
	Median	28,2	29,7	
	Q1-Q3	19.2-31.0	23.8-34.4	
Dkk1 (pmol/L) at 10 Months	n	3	4	0,377
	Median	16,3	29,8	
	Q1-Q3	9.1-26.1	18.5-34.9	
Dkk1 (pmol/L) at 12 Months	n	2	0	

	Median	14,4	.	
	Q1-Q3	8.4-20.4	.-.	
	**activin-A (pg/ml)**			
activin-A (pg/ml) at baseline	n	9	15	0,858
	Median	667,9	636,1	
	Q1-Q3	548.3-902.7	492.3-913.5	
activin-A (pg/ml) at 2 Months	n	5	12	0,874
	Median	447,5	469,8	
	Q1-Q3	321.0-517.7	374.3-550.3	
activin-A (pg/ml) at 4 Months	n	3	8	0,358
	Median	468,3	384	
	Q1-Q3	418.7-580.9	279.0-506.1	
activin-A (pg/ml) at 6 Months	n	3	6	0,897
	Median	366,9	383,5	
	Q1-Q3	300.0-695.8	372.6-504.5	
activin-A (pg/ml) at 8 Months	n	3	5	0,766
	Median	418,4	365,7	
	Q1-Q3	213.5-439.0	319.0-537.9	
activin-A (pg/ml) at 10 Months	n	3	4	0,596
	Median	362,8	320,7	
	Q1-Q3	280.5-467.5	242.4-390.1	
activin-A (pg/ml) at 12 Months	n	2	0	
	Median	287,5	.	
	Q1-Q3	256.8-318.2	.-.	
	**CCL3 (ng/ml)**			
CCL3 (ng/ml) at baseline	n	9	15	0,512
	Median	76,6	80,3	
	Q1-Q3	72.4-94.8	22.8-88.5	
CCL3 (ng/ml) at 2 Months	n	5	12	0,429
	Median	83,2	68,9	
	Q1-Q3	44.0-102.7	42.3-82.9	
CCL3 (ng/ml) at 4 Months	n	3	8	0,083
	Median	34,3	68,8	
	Q1-Q3	32.8-68.0	62.3-77.5	
CCL3 (ng/ml) at 6 Months	n	3	6	0,156
	Median	54	66,6	
	Q1-Q3	6.3-67.4	61.3-73.1	
CCL3 (ng/ml) at 8 Months	n	3	5	0,233
	Median	54,3	63,5	
	Q1-Q3	8.7-61.1	55.1-66.9	
CCL3 (ng/ml) at 10 Months	n	3	4	>0.999
	Median	14,2	53,3	
	Q1-Q3	9.1-60.8	27.0-57.0	
CCL3 (ng/ml) at 12 Months	n	2	0	
	Median	34,1	.	
	Q1-Q3	3.9-64.3	.-.	

[a] Mann-Whitney U-test

Table S16. Biomarker values at each timepoint, by SREs occurrence during study

		SREs during study		
		Yes	No	
		n (%)	n (%)	p-value[a]
	**bALP (µg/L)**			
bALP (µg/L) at baseline	n	6	18	0,868
	Median	10,7	10,9	
	Q1-Q3	10.0-11.7	8.9-11.7	
bALP (µg/L) at 2 Months	n	5	12	0,102
	Median	15,4	10,9	
	Q1-Q3	12.8-17.5	7.9-12.6	
bALP (µg/L) at 4 Months	n	3	8	0,919
	Median	10,8	11,7	
	Q1-Q3	8.9-28.2	10.2-13.5	
bALP (µg/L) at 6 Months	n	3	6	0,245
	Median	7,1	13,7	
	Q1-Q3	6.8-14.8	11.6-14.8	
bALP (µg/L) at 8 Months	n	3	5	0,371
	Median	6,8	16,9	
	Q1-Q3	5.6-16.9	15.0-18.0	
bALP (µg/L) at 10 Months	n	3	4	0,377
	Median	9,8	16,6	
	Q1-Q3	5.9-15.8	11.0-19.0	
bALP (µg/L) at 12 Months	n	1	1	
	Median	19,7	14,5	
	Q1-Q3	19.7-19.7	14.5-14.5	
	**Osteocalcin (ng/ml)**			
Osteocalcin (ng/ml) at baseline	n	6	18	0,764
	Median	7,2	9,5	
	Q1-Q3	3.9-11.9	6.4-11.2	
Osteocalcin (ng/ml) at 2 Months	n	5	12	0,429
	Median	9,4	11,4	
	Q1-Q3	5.8-10.9	9.1-14.5	
Osteocalcin (ng/ml) at 4 Months	n	3	8	0,919
	Median	12,4	14,7	
	Q1-Q3	9.9-17.7	9.9-19.3	
Osteocalcin (ng/ml) at 6 Months	n	3	6	0,156
	Median	11,1	17,5	

	Q1-Q3	10.8-12.8	13.9-25.4	
Osteocalcin (ng/ml) at 8 Months	n	3	5	0,136
	Median	8,5	17,8	
	Q1-Q3	5.6-14.9	16.9-29.8	
Osteocalcin (ng/ml) at 10 Months	n	3	4	0,377
	Median	8,9	18,7	
	Q1-Q3	3.8-16.8	10.2-23.9	
Osteocalcin (ng/ml) at 12 Months	n	1	1	
	Median	13,3	20,8	
	Q1-Q3	13.3-13.3	20.8-20.8	
	**PINP (pg/ml)**			
PINP (pg/ml) at baseline	n	6	18	0,134
	Median	662,6	477,8	
	Q1-Q3	547.4-781.6	253.1-733.1	
PINP (pg/ml) at 2 Months	n	5	12	0,317
	Median	226,3	467,8	
	Q1-Q3	139.5-710.7	281.4-777.4	
PINP (pg/ml) at 4 Months	n	3	8	0,475
	Median	411,6	502,2	
	Q1-Q3	278.5-777.5	449.4-1084	
PINP (pg/ml) at 6 Months	n	3	6	0,699
	Median	442,8	614,1	
	Q1-Q3	397.8-698.7	419.7-1694	
PINP (pg/ml) at 8 Months	n	3	5	>0.999
	Median	765,5	1004	
	Q1-Q3	381.1-3505	541.1-1988	
PINP (pg/ml) at 10 Months	n	3	4	>0.999
	Median	652	910,1	
	Q1-Q3	335.8-3650	503.3-1914	
PINP (pg/ml) at 12 Months	n	1	1	
	Median	701,3	1284	
	Q1-Q3	701.3-701.3	1284-1284	
	**CTX(ng/ml)**			
CTX(ng/ml) at baseline	n	6	18	0,92
	Median	0,8	0,7	
	Q1-Q3	0.2-1.3	0.3-0.9	
CTX(ng/ml) at 2 Months	n	5	12	0,46

	Median	0,2	0,4	
	Q1-Q3	0.1-0.6	0.3-0.6	
CTX(ng/ml) at 4 Months	n	3	8	0,919
	Median	0,3	0,3	
	Q1-Q3	0.2-0.7	0.2-0.4	
CTX(ng/ml) at 6 Months	n	3	6	0,897
	Median	0,2	0,2	
	Q1-Q3	0.1-0.7	0.2-0.4	
CTX(ng/ml) at 8 Months	n	3	5	0,371
	Median	0,1	0,2	
	Q1-Q3	0.0-0.5	0.1-0.3	
CTX(ng/ml) at 10 Months	n	3	4	0,596
	Median	0,1	0,2	
	Q1-Q3	0.1-0.4	0.2-0.3	
CTX(ng/ml) at 12 Months	n	1	1	
	Median	0,4	0,2	
	Q1-Q3	0.4-0.4	0.2-0.2	
	**TRACP-5B (U/L)**			
TRACP-5B (U/L) at baseline	n	6	18	0,301
	Median	3,7	2,7	
	Q1-Q3	2.7-4.3	1.6-3.9	
TRACP-5B (U/L) at 2 Months	n	5	12	0,635
	Median	1,8	1,9	
	Q1-Q3	1.0-1.9	1.2-2.2	
TRACP-5B (U/L) at 4 Months	n	3	8	0,61
	Median	1	1,2	
	Q1-Q3	0.7-2.0	1.0-2.0	
TRACP-5B (U/L) at 6 Months	n	3	6	0,699
	Median	1,1	1,5	
	Q1-Q3	0.9-15.9	1.2-1.9	
TRACP-5B (U/L) at 8 Months	n	3	5	0,371
	Median	1,1	1	
	Q1-Q3	0.8-2.2	0.9-1.0	
TRACP-5B (U/L) at 10 Months	n	2	3	>0.999
	Median	1,4	0,9	
	Q1-Q3	0.8-2.0	0.9-0.9	
TRACP-5B (U/L) at 12 Months	n	1	1	

	Median	1,8	0,9	
	Q1-Q3	1.8-1.8	0.9-0.9	
	**RANKL (pmol/L)**			
RANKL (pmol/L) at baseline	n	6	18	0,194
	Median	0,5	0,2	
	Q1-Q3	0.2-0.5	0.1-0.4	
RANKL (pmol/L) at 2 Months	n	5	12	0,493
	Median	0,1	0,2	
	Q1-Q3	0.1-0.2	0.2-0.2	
RANKL (pmol/L) at 4 Months	n	3	8	0,475
	Median	0,1	0,1	
	Q1-Q3	0.1-0.2	0.1-0.1	
RANKL (pmol/L) at 6 Months	n	3	6	0,053
	Median	0,2	0,1	
	Q1-Q3	0.1-0.2	0.1-0.1	
RANKL (pmol/L) at 8 Months	n	3	5	0,233
	Median	0,1	0,1	
	Q1-Q3	0.1-0.2	0.0-0.1	
RANKL (pmol/L) at 10 Months	n	3	4	0,216
	Median	0,2	0	
	Q1-Q3	0.1-0.3	0.0-0.1	
RANKL (pmol/L) at 12 Months	n	1	1	
	Median	0,2	0,1	
	Q1-Q3	0.2-0.2	0.1-0.1	
	**RANKL/OPG ratio**			
RANKL/OPG ratio at baseline	n	6	18	0,301
	Median	0,1	0,1	
	Q1-Q3	0.0-0.1	0.0-0.1	
RANKL/OPG ratio at 2 Months	n	5	12	0,562
	Median	0	0	
	Q1-Q3	0.0-0.0	0.0-0.0	
RANKL/OPG ratio at 4 Months	n	3	8	0,358
	Median	0	0	
	Q1-Q3	0.0-0.0	0.0-0.0	
RANKL/OPG ratio at 6 Months	n	3	6	0,093
	Median	0	0	
	Q1-Q3	0.0-0.0	0.0-0.0	

RANKL/OPG ratio at 8 Months	n	3	5	0,371
	Median	0	0	
	Q1-Q3	0.0-0.0	0.0-0.0	
RANKL/OPG ratio at 10 Months	n	3	4	0,052
	Median	0	0	
	Q1-Q3	0.0-0.1	0.0-0.0	
RANKL/OPG ratio at 12 Months	n	1	1	
	Median	0	0	
	Q1-Q3	0.0-0.0	0.0-0.0	
	**SOST (pmol/L)**			
SOST (pmol/L) at baseline	n	6	18	0,243
	Median	64,4	43,3	
	Q1-Q3	39.4-66.3	37.7-62.9	
SOST (pmol/L) at 2 Months	n	5	12	0,188
	Median	40,7	35,1	
	Q1-Q3	40.1-49.7	28.7-40.0	
SOST (pmol/L) at 4 Months	n	3	8	0,358
	Median	45,8	33	
	Q1-Q3	29.7-77.1	23.9-40.0	
SOST (pmol/L) at 6 Months	n	3	6	0,053
	Median	92,9	25,6	
	Q1-Q3	48.1-149.6	22.9-31.8	
SOST (pmol/L) at 8 Months	n	3	5	0,074
	Median	63,5	23,5	
	Q1-Q3	29.9-113.2	21.2-26.2	
SOST (pmol/L) at 10 Months	n	3	4	0,216
	Median	40,5	20,7	
	Q1-Q3	36.9-82.1	19.8-42.9	
SOST (pmol/L) at 12 Months	n	1	1	
	Median	35,7	20	
	Q1-Q3	35.7-35.7	20.0-20.0	
	**Dkk1 (pmol/L)**			
Dkk1 (pmol/L) at baseline	n	6	18	0,077
	Median	61,8	37,7	
	Q1-Q3	43.0-80.8	21.2-52.4	
Dkk1 (pmol/L) at 2 Months	n	5	12	0,225
	Median	58,4	30,2	

	Q1-Q3	55.3-67.5	24.2-52.3	
Dkk1 (pmol/L) at 4 Months	n	3	8	0,919
	Median	33,7	37,2	
	Q1-Q3	18.5-57.5	19.3-60.7	
Dkk1 (pmol/L) at 6 Months	n	3	6	0,245
	Median	39,7	33	
	Q1-Q3	37.0-54.8	21.2-49.2	
Dkk1 (pmol/L) at 8 Months	n	3	5	>0.999
	Median	28,2	29,7	
	Q1-Q3	23.8-31.0	19.2-34.4	
Dkk1 (pmol/L) at 10 Months	n	3	4	0,216
	Median	16,3	29,8	
	Q1-Q3	8.4-26.1	18.9-34.9	
Dkk1 (pmol/L) at 12 Months	n	1	1	
	Median	20,4	8,4	
	Q1-Q3	20.4-20.4	8.4-8.4	
	**activin-A (pg/ml)**			
activin-A (pg/ml) at baseline	n	6	18	0,714
	Median	586,5	665,2	
	Q1-Q3	421.1-921.2	524.9-902.7	
activin-A (pg/ml) at 2 Months	n	5	12	0,317
	Median	562,2	454,8	
	Q1-Q3	390.4-786.7	339.6-503.1	
activin-A (pg/ml) at 4 Months	n	3	8	0,262
	Median	468,3	384	
	Q1-Q3	418.7-904.7	279.0-506.1	
activin-A (pg/ml) at 6 Months	n	3	6	0,245
	Median	593,8	375,7	
	Q1-Q3	366.9-695.8	300.0-388.3	
activin-A (pg/ml) at 8 Months	n	3	5	0,136
	Median	439	319	
	Q1-Q3	418.4-982.7	231.9-365.7	
activin-A (pg/ml) at 10 Months	n	3	4	0,377
	Median	422,7	320,7	
	Q1-Q3	280.5-467.5	242.4-360.2	
activin-A (pg/ml) at 12 Months	n	1	1	
	Median	318,2	256,8	

	Q1-Q3	318.2-318.2	256.8-256.8	
	**CCL3 (ng/ml)**			
CCL3 (ng/ml) at baseline	n	6	18	0,194
	Median	71,7	80,8	
	Q1-Q3	62.2-79.0	61.3-98.9	
CCL3 (ng/ml) at 2 Months	n	5	12	0,874
	Median	61,2	70,8	
	Q1-Q3	45.7-102.7	41.5-86.3	
CCL3 (ng/ml) at 4 Months	n	3	8	0,185
	Median	47	68,8	
	Q1-Q3	34.3-68.0	62.3-77.5	
CCL3 (ng/ml) at 6 Months	n	3	6	**0,028**
	Median	54	69,3	
	Q1-Q3	6.3-61.2	62.1-73.1	
CCL3 (ng/ml) at 8 Months	n	3	5	0,136
	Median	21,1	63,5	
	Q1-Q3	8.7-61.1	55.1-66.9	
CCL3 (ng/ml) at 10 Months	n	3	4	0,052
	Median	9,1	57	
	Q1-Q3	3.3-14.2	53.3-59.3	
CCL3 (ng/ml) at 12 Months	n	1	1	
	Median	3,9	64,3	
	Q1-Q3	3.9-3.9	64.3-64.3	

[a] Mann-Whitney U-test

Table S17. Biomarker values at each timepoint in patients with and without disease progression

		PD		
		Yes	No	
		n (%)	n (%)	p-value[a]
	**bALP (µg/L)**			
bALP (µg/L) at baseline	n	12	12	0,403
	Median	10,6	11,3	
	Q1-Q3	8.3-11.6	10.2-11.7	
bALP (µg/L) at 2 Months	n	10	7	0,733
	Median	11,1	12,9	
	Q1-Q3	9.1-12.8	7.8-17.5	
bALP (µg/L) at 4 Months	n	6	5	0,927
	Median	11,4	11,9	
	Q1-Q3	10.8-12.9	9.1-14.1	
bALP (µg/L) at 6 Months	n	4	5	>0.999
	Median	13,7	11,6	
	Q1-Q3	10.3-14.3	8.1-14.8	
bALP (µg/L) at 8 Months	n	4	4	>0.999
	Median	16	11,8	
	Q1-Q3	10.9-16.9	4.7-18.8	
bALP (µg/L) at 10 Months	n	4	3	0,596
	Median	15,4	7	
	Q1-Q3	12.4-16.9	5.9-20.0	
bALP (µg/L) at 12 Months	n	2	0	
	Median	17,1	.	
	Q1-Q3	14.5-19.7	.-.	
	**Osteocalcin (ng/ml)**			
Osteocalcin (ng/ml) at baseline	n	12	12	0,795
	Median	8,3	9,5	
	Q1-Q3	5.5-11.6	4.7-10.8	
Osteocalcin (ng/ml) at 2 Months	n	10	7	0,157
	Median	12,5	9,4	
	Q1-Q3	9.4-14.9	2.0-12.2	
Osteocalcin (ng/ml) at 4 Months	n	6	5	0,648
	Median	12,4	17,2	
	Q1-Q3	8.6-17.7	11.2-19.2	
Osteocalcin (ng/ml) at 6 Months	n	4	5	>0.999
	Median	14,5	13,9	
	Q1-Q3	11.8-20.8	11.1-18.9	
Osteocalcin (ng/ml) at 8 Months	n	4	4	>0.999
	Median	16,4	12,7	
	Q1-Q3	10.3-23.8	7.3-28.5	
Osteocalcin (ng/ml) at 10 Months	n	4	3	0,377
	Median	17,2	3,8	
	Q1-Q3	12.8-23.0	2.8-19.7	
Osteocalcin (ng/ml) at 12 Months	n	2	0	
	Median	17,1	.	
	Q1-Q3	13.3-20.8	.-.	
	**PINP (pg/ml)**			
PINP (pg/ml) at baseline	n	12	12	0,544

	Median	507,6	554,9	
	Q1-Q3	254.0-702.4	304.0-761.8	
PINP (pg/ml) at 2 Months	n	10	7	0,526
	Median	467,8	214,8	
	Q1-Q3	333.2-710.7	139.5-1048	
PINP (pg/ml) at 4 Months	n	6	5	0,927
	Median	486,8	490,2	
	Q1-Q3	439.2-601.4	411.6-1567	
PINP (pg/ml) at 6 Months	n	4	5	0,903
	Median	570,7	439,3	
	Q1-Q3	431.2-743.8	397.8-1694	
PINP (pg/ml) at 8 Months	n	4	4	0,665
	Median	884,9	1185	
	Q1-Q3	653.3-2255	290.0-2072	
PINP (pg/ml) at 10 Months	n	4	3	0,377
	Median	956,6	447,6	
	Q1-Q3	605.5-2456	335.8-2567	
PINP (pg/ml) at 12 Months	n	2	0	
	Median	992,5	.	
	Q1-Q3	701.3-1284	.-.	
	**CTX(ng/ml)**			
CTX(ng/ml) at baseline	n	12	12	0,751
	Median	0,7	0,7	
	Q1-Q3	0.4-0.9	0.2-0.9	
CTX(ng/ml) at 2 Months	n	10	7	0,097
	Median	0,5	0,1	
	Q1-Q3	0.3-0.7	0.1-0.6	
CTX(ng/ml) at 4 Months	n	6	5	0,315
	Median	0,4	0,3	
	Q1-Q3	0.2-0.7	0.2-0.3	
CTX(ng/ml) at 6 Months	n	4	5	0,178
	Median	0,5	0,2	
	Q1-Q3	0.3-0.7	0.2-0.2	
CTX(ng/ml) at 8 Months	n	4	4	0,312
	Median	0,4	0,1	
	Q1-Q3	0.2-0.5	0.1-0.1	
CTX(ng/ml) at 10 Months	n	4	3	0,377
	Median	0,3	0,1	
	Q1-Q3	0.2-0.4	0.1-0.2	
CTX(ng/ml) at 12 Months	n	2	0	
	Median	0,3	.	
	Q1-Q3	0.2-0.4	.-.	
	**TRACP-5B (U/L)**			
TRACP-5B (U/L) at baseline	n	12	12	0,977
	Median	3,6	3	
	Q1-Q3	1.5-4.0	1.9-4.3	
TRACP-5B (U/L) at 2 Months	n	10	7	0,884
	Median	1,9	1,8	
	Q1-Q3	1.0-2.0	0.7-3.1	
TRACP-5B (U/L) at 4 Months	n	6	5	0,784

	Median	1,6	1,2	
	Q1-Q3	1.0-2.0	0.8-1.3	
TRACP-5B (U/L) at 6 Months	n	4	5	0,713
	Median	1,4	1,3	
	Q1-Q3	1.1-8.8	1.0-1.9	
TRACP-5B (U/L) at 8 Months	n	4	4	0,885
	Median	1	1	
	Q1-Q3	0.9-1.6	0.8-1.1	
TRACP-5B (U/L) at 10 Months	n	3	2	0,386
	Median	0,9	0,8	
	Q1-Q3	0.9-2.0	0.8-0.9	
TRACP-5B (U/L) at 12 Months	n	2	0	
	Median	1,3	.	
	Q1-Q3	0.9-1.8	.-.	
	**RANKL (pmol/L)**			
RANKL (pmol/L) at baseline	n	12	12	0,166
	Median	0,3	0,2	
	Q1-Q3	0.2-0.5	0.1-0.4	
RANKL (pmol/L) at 2 Months	n	10	7	0,223
	Median	0,2	0,1	
	Q1-Q3	0.2-0.2	0.1-0.2	
RANKL (pmol/L) at 4 Months	n	6	5	>0.999
	Median	0,1	0,1	
	Q1-Q3	0.1-0.2	0.1-0.1	
RANKL (pmol/L) at 6 Months	n	4	5	0,540
	Median	0,1	0,1	
	Q1-Q3	0.1-0.2	0.1-0.1	
RANKL (pmol/L) at 8 Months	n	4	4	0,885
	Median	0,1	0,1	
	Q1-Q3	0.1-0.1	0.1-0.1	
RANKL (pmol/L) at 10 Months	n	4	3	0,860
	Median	0,1	0,1	
	Q1-Q3	0.0-0.2	0.0-0.2	
RANKL (pmol/L) at 12 Months	n	2	0	
	Median	0,1	.	
	Q1-Q3	0.1-0.2	.-.	
	**RANKL/OPG ratio**			
RANKL/OPG ratio at baseline	n	12	12	0,237
	Median	0,1	0,1	
	Q1-Q3	0.0-0.1	0.0-0.1	
RANKL/OPG ratio at 2 Months	n	10	7	0,526
	Median	0	0	
	Q1-Q3	0.0-0.0	0.0-0.0	
RANKL/OPG ratio at 4 Months	n	6	5	0,927
	Median	0	0	
	Q1-Q3	0.0-0.0	0.0-0.0	
RANKL/OPG ratio at 6 Months	n	4	5	0,903
	Median	0	0	
	Q1-Q3	0.0-0.0	0.0-0.0	
RANKL/OPG ratio at 8 Months	n	4	4	0,665

	Median	0	0	
	Q1-Q3	0.0-0.0	0.0-0.0	
RANKL/OPG ratio at 10 Months	n	4	3	>0.999
	Median	0	0	
	Q1-Q3	0.0-0.0	0.0-0.0	
RANKL/OPG ratio at 12 Months	n	2	0	
	Median	0	.	
	Q1-Q3	0.0-0.0	.-.	
	**SOST (pmol/L)**			
SOST (pmol/L) at baseline	n	12	12	0,194
	Median	58,7	40	
	Q1-Q3	40.8-66.0	35.4-64.4	
SOST (pmol/L) at 2 Months	n	10	7	0,884
	Median	37,7	36,1	
	Q1-Q3	29.4-41.7	27.9-85.1	
SOST (pmol/L) at 4 Months	n	6	5	0,784
	Median	40	32,8	
	Q1-Q3	25.0-45.8	29.7-33.2	
SOST (pmol/L) at 6 Months	n	4	5	0,903
	Median	40	25,8	
	Q1-Q3	26.7-98.9	25.5-63.1	
SOST (pmol/L) at 8 Months	n	4	4	0,665
	Median	26,7	35,2	
	Q1-Q3	22.0-46.7	23.7-78.7	
SOST (pmol/L) at 10 Months	n	4	3	0,860
	Median	29	40,5	
	Q1-Q3	20.2-59.5	20.2-64.7	
SOST (pmol/L) at 12 Months	n	2	0	
	Median	27,8	.	
	Q1-Q3	20.0-35.7	.-.	
	**Dkk1 (pmol/L)**			
Dkk1 (pmol/L) at baseline	n	12	12	0,931
	Median	41,6	42,4	
	Q1-Q3	33.4-62.6	23.1-65.6	
Dkk1 (pmol/L) at 2 Months	n	10	7	0,961
	Median	43,3	36,9	
	Q1-Q3	24.4-62.5	26.9-67.5	
Dkk1 (pmol/L) at 4 Months	n	6	5	0,784
	Median	45,6	27,8	
	Q1-Q3	22.8-58.4	18.5-46.5	
Dkk1 (pmol/L) at 6 Months	n	4	5	>0.999
	Median	35,8	37	
	Q1-Q3	26.6-47.3	34.0-49.2	
Dkk1 (pmol/L) at 8 Months	n	4	4	0,885
	Median	29,6	26,8	
	Q1-Q3	23.7-32.7	14.9-40.5	
Dkk1 (pmol/L) at 10 Months	n	4	3	0,860
	Median	21,2	28,7	
	Q1-Q3	12.7-28.5	8.4-38.7	
Dkk1 (pmol/L) at 12 Months	n	2	0	

	Median	14,4	.	
	Q1-Q3	8.4-20.4	.-.	
**activin-A (pg/ml)**				
activin-A (pg/ml) at baseline	n	12	12	0,583
	Median	652	609,6	
	Q1-Q3	551.2-846.0	466.1-903.5	
activin-A (pg/ml) at 2 Months	n	10	7	0,071
	Median	503,1	390,4	
	Q1-Q3	447.5-627.9	315.5-462.2	
activin-A (pg/ml) at 4 Months	n	6	5	>0.999
	Median	443,5	412,5	
	Q1-Q3	355.4-492.5	334.5-519.6	
activin-A (pg/ml) at 6 Months	n	4	5	0,903
	Median	372,8	388,3	
	Q1-Q3	333.4-537.3	372.6-504.5	
activin-A (pg/ml) at 8 Months	n	4	4	0,312
	Median	325,2	451,8	
	Q1-Q3	222.7-428.7	342.3-760.3	
activin-A (pg/ml) at 10 Months	n	4	3	0,860
	Median	323,3	357,5	
	Q1-Q3	282.1-415.1	201.0-422.7	
activin-A (pg/ml) at 12 Months	n	2	0	
	Median	287,5	.	
	Q1-Q3	256.8-318.2	.-.	
**CCL3 (ng/ml)**				
CCL3 (ng/ml) at baseline	n	12	12	0,157
	Median	71,7	80,8	
	Q1-Q3	16.5-85.2	74.6-96.8	
CCL3 (ng/ml) at 2 Months	n	10	7	0,354
	Median	79,7	61,2	
	Q1-Q3	39.0-95.5	44.0-71.2	
CCL3 (ng/ml) at 4 Months	n	6	5	0,121
	Median	62,3	69,6	
	Q1-Q3	34.3-68.0	68.0-82.9	
CCL3 (ng/ml) at 6 Months	n	4	5	0,540
	Median	60,7	62,1	
	Q1-Q3	30.1-70.3	61.3-71.1	
CCL3 (ng/ml) at 8 Months	n	4	4	0,885
	Median	57,7	59,3	
	Q1-Q3	31.5-75.0	38.1-65.2	
CCL3 (ng/ml) at 10 Months	n	4	3	>0.999
	Median	32,4	56	
	Q1-Q3	11.6-55.7	3.3-57.9	
CCL3 (ng/ml) at 12 Months	n	2	0	
	Median	34,1	.	
	Q1-Q3	3.9-64.3	.-.	

[a] Mann-Whitney U-test
